# Mapping behavior change techniques and health data combinations in virtual agents for chronic condition management: A systematic scoping review

**DOI:** 10.1371/journal.pdig.0001604

**Published:** 2026-07-28

**Authors:** Martha S. Kreuzberg, Sandra Van Mellaert, Lean L. Kramer, Monique Tabak, Saskia Kelders

**Affiliations:** 1 Department of Health, Psychology and Technology, Faculty of Behavioral Management & Social Sciences, University of Twente, Enschede, the Netherlands; 2 Department of Biomedical Signals and Systems, Faculty of Electrical Engineering, Mathematics, and Computer Science, University of Twente, Enschede, the Netherlands; Yale University, UNITED STATES OF AMERICA

## Abstract

Chronic conditions such as cardiovascular disease, diabetes, and cancer require sustained lifestyle changes and self-management, yet traditional care models often provide limited support for long-term behavior change. Digital health technologies, particularly virtual agents, computer-generated characters simulating human-like interactions through verbal and nonverbal cues, offer new ways to provide personalized, scalable, and continuous support. However, the ways in which distinct components within such digital health technologies, including those used in chronic care interventions, are chosen and combined remain underreported. We conducted a systematic scoping review to map how behavior change techniques (BCTs), health data types, and delivery channels are rationalized, combined, and applied in virtual agent-delivered interventions for chronic condition management. The review followed established scoping review frameworks and adhered to PRISMA-ScR reporting guidelines. A search was performed across PubMed, Scopus, PsycInfo, WebofScience, and IEEE Xplore in September 2024. Twenty-one studies met the inclusion criteria. We examined the rationales reported by authors for intervention design, categorized as theory-driven, practice-driven, empirically-driven, mixed, or not explicitly stated. Few studies explained why they selected specific techniques or how health data and delivery channels were intended to interact. Across studies, BCTs were identified but often not explicitly labelled. The most common agent-delivered techniques were self-monitoring, feedback, instruction on how to perform a behavior, and prompts and cues. These techniques were typically supported by subjective self-reports (e.g., symptoms, behaviors), objective data (e.g., step counts, blood pressure), adherence data (e.g., activity completion) and user preference data (e.g., preferred timing of reminders). Delivery channels comprised smartphone or tablet apps. This review provides the first systematic map of how BCT-health data-delivery channel combinations are applied in virtual agent interventions for chronic condition management. It highlights foundational design patterns and reporting gaps, emphasizing the need for transparent, theory-informed reporting to guide future development of adaptive, evidence-based digital health tools.

## Author summary

Virtual agent interventions are increasingly developed to support people with chronic conditions. These interventions can provide ongoing support by reminding patients about healthy -behaviors, giving feedback on their progress, and helping them manage symptoms at home. However, little is known about how these interventions are designed and how different components are combined. In this scoping review, we examined how virtual agent-delivered interventions for chronic care report their design choices, which strategies to change behavior they use, what kinds of health data they rely on, and through which channels these are delivered. Twenty-one studies met our inclusion criteria. We found that most studies gave only general reasons for their intervention design, without clearly explaining why particular components were chosen. These were typically linked to self-reported symptoms or health-device collected data and delivered through smartphone or tablet apps. These findings point to a need for clearer reporting of design choices in future interventions.

## Introduction

Chronic conditions, are leading causes of morbidity and mortality worldwide. In this review, the focus is on virtual agent-delivered interventions targeting physical chronic conditions, such as cardiovascular diseases, diabetes, cancer, and chronic pain. Since they require continuous health management, they pose significant socioeconomic challenges to healthcare systems [1]. Effective management often depends on patients’ ability to commit to sustained lifestyle changes, such as an improved diet, medication adherence, increased physical activity, sufficient sleep, and engaging in regular health monitoring [2]. Yet, traditional approaches like periodic in-person visits to healthcare providers can fall short in supporting patients’ ongoing efforts, as they provide limited opportunities for long-term, personalized feedback and coaching [3], which is essential for sustaining behavior change [4]. Digital health technologies, particularly virtual agents, offer novel opportunities for real-time monitoring and tailored feedback, potentially making chronic condition management more scalable, personalized, and accessible [5].

Virtual agents are computer-generated characters designed to simulate human-like interactions through verbal and nonverbal cues [6]. In lifestyle-based interventions, virtual agents commonly take the form of conversational agents (CAs), which are text- or voice-based dialogue systems, or embodied conversational agents (ECAs), which are animated, visually represented agents capable of simulating face-to-face interaction. In health contexts, such agents are often designed to function as virtual coaches, providing structured guidance and support for behavior change. They are used to support a range of health behaviors such as dietary intake, physical activity, stress, sleep, and dementia care [7–10]. Virtual agent-delivered interventions can help manage health behaviors by providing personalized feedback and reminders, often using data from health-tracking devices including physiological indicators like heart rate and glucose levels. Prior research shows that virtual agents can support dietary counseling, promote screening and self-management in conditions like cancer, atrial fibrillation, and type 2 diabetes, and encourage physical activity among people with cardiovascular conditions [11,12]. While increasingly used in healthcare, the long-term efficacy of diverse types virtual agents in chronic condition management remains under investigation.

By offering scalable and continuous interaction, virtual agents can address common limitations in traditional care such as limited contact time and delayed feedback [11]. These agents range in complexity and adaptability [13]. Rule-based systems operate through predefined “if-then” logic to process input and generate responses, offering transparency and predictability [14], while artificial intelligence (AI)-driven agents can leverage machine learning and natural language processing to generate adaptive and personalized responses [15]. Rule-based agents are often favored in health care research settings due to their reliability and predictable responses. However, their reliance on predefined logic limits flexibility when handling unstructured interactions with patients [16]. Meanwhile, AI-driven agents can integrate real-time patient data for tailored interventions but face challenges in interpretability, consistency, and bias [17,18].

This shift from rule-based to AI-driven approaches highlights personalization as a central promise, but also a challenge, of virtual agents. Personalization has been shown to improve engagement and health outcomes in digital health interventions [19]. In a review on cancer-focused digital health interventions, Hwang and Jiang [19] identified that personalization was achieved through data-driven approaches in the majority of studies (such as using wearables or patient-reported outcomes), though gaps remain, particularly in understanding how different personalization strategies work individually and in combination. Still, prior reviews have highlighted emerging AI assistants and chatbots as promising technologies for advancing personalization delivery [20].

Building on this, this review conceptualizes personalization in virtual agent-delivered chronic care interventions as the integration of health data inputs. This can involve information such as medical history, symptoms, biometric indicators, treatment records, and lifestyle factors, either directly provided by the patient or inferred from their input [21]. For the purpose of this review, a distinction is made between different types of health data (see **Table 1** in the Methods for a detailed overview). Within this distinction, objective health data refers to quantifiable data collected through sensors and devices, such as step counts, heart rate, glucose levels and sleeping patterns. For people with metabolic syndrome for example, common measures include blood pressure and high-density lipoprotein cholesterol [22]. In contrast, subjective health data comprises self-reported data from individuals regarding their health status, perceived symptoms, or behaviors, including dietary intake, pain levels, and mood [23], providing insights into participants’ personal perceptions and experiences. Other forms of health data, such as contextual or environmental health data [24,25], user preference data [26], and adherence data [27], can further inform how virtual agents tailor support. To operationalize these types of data, virtual agents often rely on behavior change techniques (BCTs), which provide structured strategies for influencing behavior.

**Table 1:** Health data types.

Type of Data	Description	Examples
**Objective Health Data**	Quantifiable data collected through sensors and devices	Step counts, heart rate, glucose levels, sleep patterns, blood pressure, HDL-cholesterol [22]
**Subjective Health Data**	Self-reported data regarding a person’s health status, perceived symptoms, or behaviors.	Dietary intake, pain levels, mood [23]
**Contextual Data**	Data about the user’s behavioral & situational context; such as the activity the person is doing and where they are	User location, activity classification, time of day, purpose of action [24,25]
**Environmental Data**	Objective measurements of external conditions that may affect a person’s health	Atmospheric conditions, pollution levels, weather [40]
**User Preference Data**	Information about an individual choices or priorities related to their engagement with a digital health intervention	User selections on communication needs, styles or desires for autonomy or assistance [26]
**Adherence Data**	Tracking how consistently patients follow prescribed treatment regimens	Medication adherence, treatment consistency [27]

BCTs are the smallest active components of an intervention, designed to influence behavior by targeting its underlying mechanisms in a clearly defined, observable, and replicable way, such as through feedback, self-monitoring, or reinforcement [28]. In virtual agent-delivered interventions, these techniques are often embedded within interactions and delivered through specific delivery channels such as smartphone or tablet apps, web-based platforms, or a combination of them [29]. Each of these channels can enable different delivery mechanisms, such as push notifications or dashboards, that shape how the BCT is implemented. The integration of BCTs with collected health data and how these combinations are delivered by the virtual agent through the selected delivery channels, can significantly influence how effectively an intervention promotes behaviour change [30]. However, most studies do not clearly describe how BCTs, health data, and delivery channels are combined [30], which limits our understanding of how behavior change mechanisms are activated in practice and how virtual agent-delivered interventions actually achieve their effects.

In addition, the theoretical and practical rationales underlying these design choices are often insufficiently articulated. Reviews of conversational agents in chronic healthcare have found that a substantial proportion of studies are not explicitly informed by behavior change theory [29,31]. At the same time, evidence from other domains suggests that stronger theoretical grounding may enhance intervention effectiveness [32]. For example, a meta-analysis of digital eating disorder interventions found that interventions with higher theory coding scheme scores demonstrated significantly greater improvements in outcomes compared to less theory-informed interventions [33]. Together, these findings highlight the importance of examining how design decisions in virtual agent-delivered chronic care interventions are justified. However, limited prior work has systematically categorized how such rationales are articulated in virtual agent-delivered interventions, indicating a need for exploratory mapping approaches.

**Fig 1:**
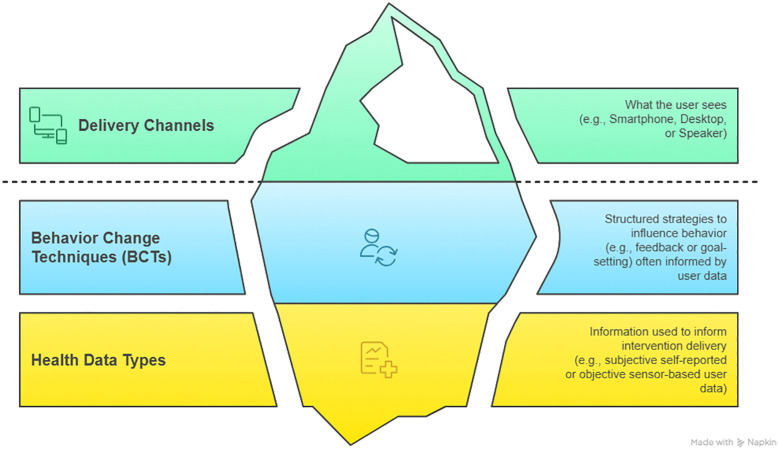
Conceptual model of virtual agent-delivered intervention architecture.

In this systematic scoping review, virtual agent-delivered interventions are conceptualized as systems composed of three interacting components: health data inputs, BCTs, and delivery channels. Fig 1 illustrates how virtual-agent delivered interventions can be conceptualized as the integration of these three interacting components. Thus, this systematic scoping review aims to shed light on the intervention architectures underlying virtual agent-delivered chronic care interventions and to explore their patterns by addressing the following questions:

1. What theoretical or practice-based rationales do authors provide for the use and combination of BCTs, health data types, and delivery channels in virtual agent-delivered interventions for chronic condition management?
2. What BCTs are used in combination with specific types of health data and delivery channels in virtual agent-delivered interventions for chronic condition management?
3. How prevalent are different combinations of BCTs, health data types and delivery channels in virtual agent-delivered interventions for chronic conditions and in what contexts are they applied (e.g., study population, intervention aim, setting)?

## Materials and methods

We chose to conduct a systematic scoping review following the framework steps described by Arksey and O’Malley [34], with additional guidance from the Joanna Briggs Institute (JBI) for conducting systematic scoping reviews. The reporting aligns with the PRISMA extension for Scoping Reviews (PRISMA-ScR) [35] (see S1 Checklist). This scoping review did not include a formal study quality assessment, as its objective was to map the scope of interventions rather than evaluate their effectiveness. For methodological transparency and reproducibility purposes, the study protocol is registered in the Open Science Framework (OSF) under the link https://osf.io/6tved/ (see S1 Text).

### Search strategy

A literature search was conducted during September 2024 using PubMed, Scopus, PsycINFO, Web of Science and IEEE Xplore. These particular databases were chosen, as they ensure broad coverage of high-impact research across diverse fields such as psychology and behavioral sciences, engineering and technology-focused studies and health and medical informatics. Search queries were adapted per database using the commonly recommended PCC structure (population, concept, context) [36] of key concepts and Boolean operators: Virtual agents (e.g., “chatbot”, “virtual assistant”, “conversational agent”, embodied agent”); Healthcare and digital interventions (e.g., “mHealth”, “telehealth”, “behavior change intervention”); Chronic conditions (e.g., “diabetes”, “heart disease”, “cancer”, “COPD”), next to broader terms such as “chronic disease” and “chronic condition” to ensure coverage across a wide range of chronic conditions.

The final selection of keywords was informed by prior reviews in the field of conversational agents in healthcare [12,29,37] and consultation of an information specialist and disciplinary experts. The reference lists of included studies were also screened to identify additional relevant publications. A complete overview of search terms for each concept can be found in S2 Text.

### Eligibility criteria

Studies were selected according to predefined inclusion criteria (see Box 1), based on the key concepts of the search string. No date restrictions were applied, meaning studies were considered from the earliest records available in each database up to the search date (10^th^ September 2024). Only English-language, peer-reviewed studies available in full text were included. Given the focus on empirically evaluated virtual agent–delivered interventions with longitudinal or real-world testing, gray literature was excluded to ensure sufficient methodological detail and transparency for the structured extraction of intervention components.

**Box 1. Eligibility Criteria**

**Study design & source type**

- Empirical studies with primary data from randomized controlled trials (RCTs), quasi-experimental, longitudinal, observational, and qualitative designs.
- Peer-reviewed journal articles and conference papers (no preprints, protocols editorials, or reviews).

**Population**

- Adults (≥ 18 years) diagnosed with a chronic condition were eligible, defined as a long-term health problem that requires ongoing medical care or limits daily activities. This allowed for the inclusion of hypertension, obesity, arterial fibrillation, dementia, and cancer in remission when ongoing surveillance or management was required.
- Mental health conditions were excluded as a primary intervention target.

**Intervention type**

- Must involve a virtual agent as a core component of the intervention.
- The virtual agent must exhibit conversational interactivity, responding to user input (whether rule-based or AI-driven)
- The intervention must:
  - Target behavior/lifestyle changes and/or self-management in chronic conditions
  - Incorporate health data (subjective or objective, e.g., self-reported symptoms, wearable data, medical records)
  - must incorporate behavior change techniques (BCTs), either explicitly reported or identifiable based on the intervention description using the BCT Taxonomy v1

**Outcomes assessed**

- Studies must report on at least one of the following outcomes:
  - Behavior change outcomes (e.g., physical activity, dietary habits, medication adherence).
  - Health outcomes (e.g., weight loss, blood pressure, HbA1c levels).
  - User experience outcomes (e.g., usability, acceptability, engagement, patient-reported feedback).

**Context**

- Real-world intervention settings, including clinical, community-based, home-based digital health environments.

### Study selection

Search results were imported into Covidence, where duplicates were automatically removed and a two-stage screening process was conducted. Firstly, the title and abstract screening was done by three independent reviewers, who assessed relevance based on inclusion criteria. In this step, the second reviewer (SV) screened 20% of the abstracts independently of the first author (MK), to identify relevant articles against the inclusion criteria. The inter-rater agreement (Cohen’s Kappa = 0.87) was evaluated after 10% to assess consistency and discrepancies were resolved through discussion or third-reviewer arbitration. To support consistency during this stage, clarification notes were documented regarding the inclusion of conditions such as Alzheimer’s, Parkinson’s, chronic pain, and obesity, and the exclusion of chronic mental illness, general undiagnosed but overweight populations, and Q&A-based chatbots with unclear intervention context. In the second step, namely the full-text screening process, eligibility was confirmed based on inclusion criteria. During the second part, the first author (MK) thoroughly screened the full text versions of the articles, ensuring they met the full text inclusion criteria. Articles meeting said inclusion criteria were moved to data extraction in Covidence.

### Data extraction

The data extraction template was informed by the PRISMA-ScR and JBI guidelines for scoping reviews [36,38], incorporating structured elements from the Behavior Change Taxonomy v1 [28]. While our data extraction partially aligned with the TIDIeR checklist [39] to ensure detailed mapping of intervention characteristics and elements, some items were not systematically captured (e.g., intervention modifications during the study, planned fidelity strategies, and fidelity of delivery), as they fell outside the scope of our scoping review objectives.

Box 2 provides an overview of the main extraction categories used, grouped by thematic area. A full version of the data extraction template is available in S1 Table.

**Box 2. Data extraction items**

- **Study Information**: Study title, ID, authors, publication year, country and journal
- **Study Characteristics**: Study aim, design (e.g., RCT, observational, mixed-methods), blinding, control group presence
- **Population Characteristics**: Target population (chronic condition type), sample size, mean age, inclusion/exclusion criteria, recruitment methods.
- **Intervention Details**: Intervention name, duration, frequency, focus (e.g., behavior change, medication adherence, self-management) and delivery channels
- **Virtual Agent Characteristics**:
  - Agent type (Conversational Agent, Embodied Conversational agent, Virtual Coach, Avatar)
  - System type (rule-based, AI-driven, hybrid models)
  - Role within intervention (sole medium or blended care)
- **Behavior Change Techniques (BCTs)**:
  - Categorized based on the Behavior Change Technique Taxonomy v1 (e.g., including goal-setting, self-monitoring, feedback, social support)
  - Where BCTs were not labelled in the text, they were inferred from intervention descriptions using standardized definitions.
- **Health Data Integration**:
  - Types of data input (e.g., physiological, self-reported, environmental), data collection tools (e.g., wearables, apps), and data output (e.g., visual feedback, recommendations).
- **Delivery Channels**:
  - E.g., smartphone apps, web platforms, text messaging (SMS), voice assistants, emails.
- **Study Outcomes**:
  - Primary outcomes (e.g., behavior change, health outcomes, medication adherence)
  - Outcome measurement methods (e.g., self-reported data, physiological assessments, clinician evaluations)
- **Study Evaluation Metrics**:
  - User satisfaction, adherence rates, engagement metrics, technical usability, retention/dropout rates
  - Measurement tools (e.g., System Usability Scale (SUS), surveys, interviews, focus groups)

BCT identification and coding were performed by the first author, who has a background and experience with behavior change intervention design using the BCT Taxonomy and the CeHRes Roadmap. Coding decisions were reviewed and discussed with a third co-author (SK), with expertise in eHealth intervention design to ensure consistency and validity. Any uncertainties in classification were resolved through discussion and consensus. The main extraction categories (e.g., BCTs, health data, delivery channels) were defined a priori based on the study objectives and existing frameworks, while subcategories were refined iteratively during the extraction process (e.g., differentiation between health data input and output).

To support the categorization of health data types in the included studies, we also used a typology based on common patterns in the literature and observed intervention characteristics. These categories are summarized in Table 1.

### Data synthesis

Data synthesis followed the same methodological frameworks (Arksey and O’Malley, JBI, PRISMA-ScR) and combined conducting a descriptive and qualitative content analysis of the extracted data. In line with the review objective, said analysis aimed to map how BCTs, health data types, and delivery channels are rationalized, combined, and applied in virtual agent-delivered interventions for chronic condition management.

Descriptive statistics (counts and percentages) were calculated using Microsoft Excel based on the extracted variables. These statistics summarize the distribution of study designs, target populations, types of chronic conditions, intervention settings and virtual agent system types.

A qualitative content analysis was conducted to examine how authors justified intervention design choices, following the approach described by Elo and Kyngäs [41]. Descriptions of the rationales for intervention design choices reported in the included studies were reviewed and coded by the first reviewer. Codes capturing different types of justification were iteratively grouped into broader categories through comparison across studies. To ensure consistency in interpretation, each study’s rationale was categorized using a structured coding scheme developed inductively from the data. Here, “rationale” refers to explanations provided by study authors for selecting intervention components, or broader design approaches (e.g., theoretical grounding or prior empirical evidence). Five rationale categories were identified: *theory-driven* (e.g., intervention design grounded in behavioral theories such as Self-Determination Theory or COM-B), *practice-driven* (e.g., design decisions informed by usability considerations, patient feedback, or clinical implementation needs), *empirically-driven* (based on findings from prior empirical studies without explicit theoretical grounding), *mixed* (combining multiple of the former categories), and *not explicitly stated*.

Intervention components were also coded according to whether they were delivered directly by the virtual agent or by other elements of the broader digital intervention system. In some studies – particularly older or hybrid intervention designs – the reporting of the system architecture was limited, which made this distinction less explicit. The found combinations were then charted to identify frequently occurring patterns across intervention contexts (e.g., diabetes vs. cancer management).

## Results

### Search results

**Fig 2:**
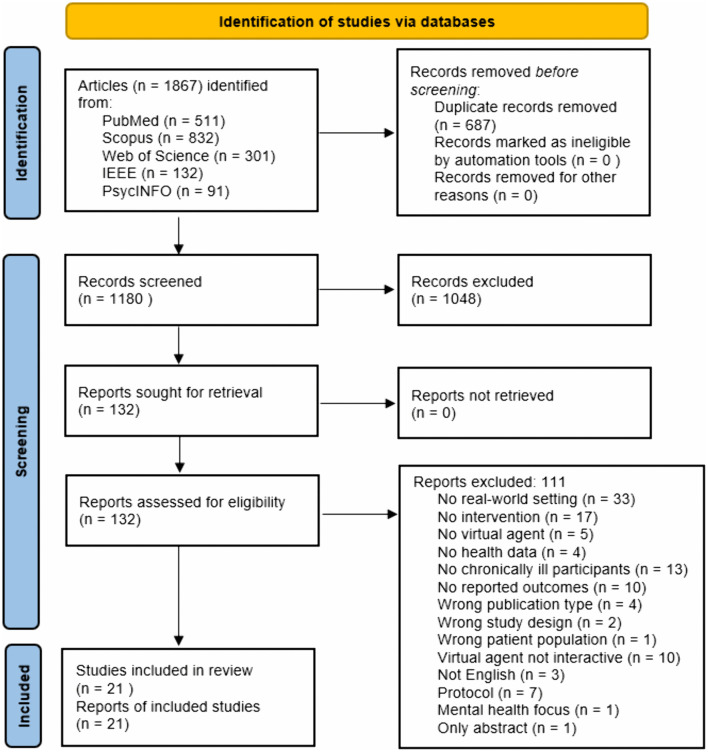
PRISMA flowchart outlining the article selection process (adapted from: https://guides.lib.unc.edu/prisma).

As can be seen in Fig 2, a total of 1867 records were identified through database searches. Of these, 687 duplicates were automatically removed using Covidence’s deduplication tool. The remaining 1180 records were screened based on title and abstract. After initial screening, 1048 records were excluded for not meeting the inclusion criteria. The full texts of 132 reports were then assessed for eligibility, leading to the exclusion of 111 articles for reasons such as lack of real-world setting, intervention, virtual agent or health data, or insufficient reporting of relevant elements. Ultimately, 21 studies met all inclusion criteria and were included in the final scoping review.

### Study characteristics

An overview of study characteristics is presented in Table 2. Among the included studies, the majority comprised of randomized controlled trials (n = 12, 57.1%), followed by observational studies (n = 6, 28.6%) and mixed-methods designs (n = 2, 9.5%). One study (4.8%) used a pilot usability approach. Study populations primarily focused on cancer (n = 6, 28.6%), diabetes (n = 5, 23.8%), and cardiovascular or heart-related conditions (n = 7, 33.3%). Among the cardiometabolic interventions, 2 tackled multi-morbidity [42,43]. All studies were conducted in home-based settings, with 2 studies (9.5%) additionally incorporating components delivered in clinical or community contexts.

**Table 2:** Study characteristics.

Study	Study Country	Study Design	Intervention Focus/Aims	Context (Setting & Target Population, Sample size)	Virtual Agent/ System Type	Main findings/outcomes
Albino de Queiroz et al., (2023) [44]	Brazil	Quasi-experimental, prospective non-randomized clinical	Monitor and improve engagement of Colorectal cancer patient in active treatment	Home-based between chemo sessions, 30 adults with colorectal cancer	CA, with AI & IoT (NLP and NLU), intent matching	More accurate self-reporting of symptoms & adverse effects during active treatment phase, high usability ratings
Baptista et al. (2020) [45]	Australia	RCT, mixed-methods	Deliver self-management education & support people with T2D	Home-based, 66 adults with T2D	ECA, rule-based	ECA found acceptable/friendly, but no direct behavioral changes
Chaix et al. (2019) [46]	France	Prospective, observational	Evaluate chatbots role in breast cancer patients medication adherence & support	Home-based, 958 adults (breast cancer patients in remission)	CA, rule-based & limited AI adaptation	Average medication adherence compliance improvement by 20% and high patient satisfaction
Echeazarra et al. (2021) [47]	Spain	RCT, 2-arm	Help hypertension patients self-monitor their blood pressure & improve self-management	Home & clinical setting, 88 adults with hypertension	CA, rule-based	Significant BP measurement knowledge & skills gain in chatbot group compared to control, high usability/ satisfaction rates among participants
Gomaa et al. (2023) [48]	United States	Mixed-methods, two-phase pilot	Evaluate Feasibility, Usability & Patient Acceptance of a hybrid text-messaging and chatbot system for real-rime self-management support of gastrointestinal cancer patients undergoing chemo	Home-based, 27 adults with gastro-intestinal cancer	CA, rule-based	Improved patient activation scores, reduction in chemo symptom severity & distress, higher self-management confidence, high satisfaction with the intervention & positive ease of use
Gong et al. (2020) [49]	Australia	RCT, 2-arm	Evaluate adoption, use & effectiveness of the My Diabetes Coach Program, an app-based ECA for diabetes self-management over 12 months in a home-setting	Home-based, 57 adults with T2D	ECA, rule-based	Improvement in health-related quality of life at 6 and 12 months, anxiety improved at 6 months but not at 12, participants rated ECA as helpful, competent & trustworthy, though maintaining engagement over 12 months was challenging
Gómez et al. (2008) [50]	Spain, Germany & Switzerland (EU project)	RCT & mixed-method evaluation	Develop & evaluate Feasibility, Clinical utility & potential cost-benefit of INCA system (PDA based telemedical artificial pancreas), integrating continuous glucose monitoring, insulin pump control and remote clinician support	Home-based, 24 adults with T1D	PDA, rule-based with predictive control elements	Significant improvements in HbA1c and Fructosamine compared to standard care (in trial 1, not 2), improved patient confidence in adjusting insulin doses and greater satisfaction when remote feedback available
Hauser-Ulrich et al. (2020) [51]	Switzerland	Pilot RCT	Evaluate feasibility & acceptance of a fully automated text-based healthcare chatbot, using cognitive behavioral therapy for chronic pain self-management & assess intervention effectiveness	Home-based, 61 adults with chronic pain	CA, rule-based	Significant reduction in pain intensity & significant increase in well-being, positive user-feedback on chatbot empathy & support
Huang et al. (2023) [52]	Taiwan	Retrospective cohort	Evaluate whether a chatbot-based collection of patient-reported symptoms during chemotherapy treatment, with automated alerts to clinicians for severe or worsening symptoms, decreases emergency department use and reduces unscheduled hospitalizations	Home-based, 63 adults with gynaecologic malignancies in chemotherapy	CA, rule-based	Patients using chatbot had lower adjusted incidence rate ratios for emergency department visits and unscheduled hospitalizations compared to usual-care patients, high user satisfaction
Krishnakumar et al. (2021) [53]	India	Longitudinal observational, real-world cohort analysis	Assess real-world effectiveness of the Wellthy CARE digital therapeutic platform on improving glycemic control and self-management in South Asian adults with T2D	Home-based, 102 adults diagnosed with T2D	CA, hybrid (rule-based & generative AI)	Significant decrease in HbA1c levels, fasting blood glucose and postprandial blood glucose levels after 16 weeks. Higher participation levels led to improved glycemic control
Magnani et al. (2017) [54]	United States	Observational feasibility pilot	Determine feasibility and acceptability of a relational agent (RA) coupled with a mobile ECG device in promoting anticoagulation adherence, arterial fibrillation symptom monitoring, and arterial fibrillation self-management	Home-based rural community, 31 adults diagnosed with arterial fibrillation	ECA, rule-based	Significant improvements in medication adherence and improvements in health-related quality of life, high patient satisfaction and engagement with ECA
Qiu et al. (2021) [55]	United States	Observational pilot	Test the system design of the smart speaker Nurse Amie, aimed at improving the quality of life of women with metastatic breast cancer, using the data from a preliminary study to discuss its feasibility, acceptability and future directions	Home-based, 6 women diagnosed with metastatic breast cancer	Smart speaker CA, rule-based	Reported increase in participant symptom awareness, self-management knowledge & intention to exercise. Some decline in engagement after first week of usage
Roca et al. (2021) [56]	Spain	Observational Pilot	Validate the effectiveness of a healthcare virtual assistant, integrated within messaging platforms, with the aim of improving medication adherence in patients with comorbid T2D and depressive disorder	Home-based, 13 patients diagnosed with T2D & depressive disorder + system evaluation of 5 involved nurses	CA, rule-based	Improvements in HbA1c and medication possession ratio, reduced appointment frequency. High reported patient satisfaction & ease of use
Sakane et al. (2023) [57]	Japan	RCT	Determine the efficacy of an SHG mobile health app (KENPO-app) in facilitating weight loss in Japanese adults with obesity and hypertension	Home-based, 74 adults diagnosed with obesity & hypertension	CA, rule-based + limited AI adaptation	95% retention; significantly greater adherence to self-weighing, pedometer use, and BP monitoring; greater weight and BMI reduction at 3 months; improved healthy behaviors (e.g., step count, eating slowly); personality traits associated with weight loss
Schläpfer et al. (2024) [58]	Austria, Switzerland & Germany	RCT	Examine the engagement at both a micro and macro level with a newly developed relaxation and mindfulness app to reduce distress in people with cancer (CanRelax App 2.0) in a fully automated RCT over 10 weeks	Home-based + clinical-setting, 210 adults diagnosed with cancer	CA, rule-based	62% retention at 10 weeks; 72% of relaxation exercises and 714 coaching sessions completed in-app; all feedback (52%) was positive. App use not linked to baseline traits. 28% of relaxation exercises were performed outside the app, and self-efficacy remained high. Participants increased their weekly relaxation goals, suggesting positive behavioral engagement beyond app use
Schlieter et al. (2024) [42]	Germany, Italy, Spain, Romania	Pilot RCT	Develop and evaluate a virtual coaching (VC) system for supporting older patients’ home rehabilitation	Home-based, 80 adults diagnosed with either neurological profiles (stroke, parkinsons) or cardiological profiles (heart failure, ischemic heart disease)	ECA, rule-based & limited AI (machine learning)	Improvement in QoL across all pathologies; positive adherence rates to prescribed rehabilitation programmes; high usability and engagement metrics.
Shamekhi et al. (2017) [59]	United States	RCT	Design, develop, and evaluate a conversational agent “Gabby” that assists patients with stress and chronic pain in conjunction with the weekly group visits	Home-based, 154 adults diagnosed with chronic pain & depression	ECA, rule-based	Significant improvements in stress management behaviors compared to controls, high satisfaction & perceived helpfulness of ECA
Ter Stal et al. (2021) [43]	Netherlands	Mixed-methods exploratory	Explore how patients perceive the design of an ECA within an eHealth self-management intervention over time, specifically regarding agent characteristics and interaction	Home-based, 11 adults diagnosed with both chronic obstructive pulmonary disease and chronic heart failure	ECA, rule-based	Patient perceptions of agent characteristics did not change over time, reliability perceived as limited due to perceived non-personalization of content. Likeliness to follow agent advice significantly decreased after 3 weeks
Sweidan et al. (2023) [60]	Jordan	Pilot usability	Develop and evaluate an innovative Alzheimer’s care system (ACS) smartphone application providing support for patients, caregivers, and doctors, especially targeting Arabic-speaking regions	Home-based, 213 adults (patients with alzheimer’s, caregivers, healthcare providers, health-care workers)	CA, rule-based + limited AI-adaptation	Generally positive usability, satisfaction and acceptance ratings.
Wonggom et al. (2020) [61]	Australia	RCT, multi-centred	Evaluate the effectiveness of an avatar education app to improve HF patient knowledge and self-care behaviors	Home-based, 36 adults diagnosed with heart failure	ECA + avatar, rule-based	At 90 days, intervention group had significantly higher heart failure knowledge, no difference in healthcare use or self-care behavior. 91.3% satisfaction with ECA among patients
Zisis et al. (2021) [62]	Australia	RCT	Determine whether an avatar-based heart failure app could improve health outcomes in heart failure, by enhancing heart failure knowledge and improving patient quality of life and self-care behavior	Home-based (post-discharge), 166 adults admitted with acute decompensated heart failure with high risk for readmission	Avatar-based CA	Improvements in self-care behaviors in enrolled patients; no differences in heart failure knowledge or quality of life between groups; high drop-out rate due to age & low engagement due to digital literacy barriers

**Abbreviations**: CA = conversational agent; ECA = embodied conversational agent; AI = artificial intelligence; IoT = Internet of Things; NLP = natural language processing; NLU = natural language understanding; PDA = personal digital assistant; T1D = type 1 diabetes; T2D = type 2 diabetes; RCT = randomized controlled trial.

Regarding the type of virtual agents used, the majority were rule-based conversational agents or rule-based embodied conversational agents (ECAs), found in 17 studies (81%). Hybrid models incorporating AI-driven adaptation or generative AI elements were represented in 5 studies (24%), mostly in combination with rule-based systems. No interventions used fully autonomous generative AI systems. Among the hybrid systems, AI capabilities were generally limited to intent recognition or personalized response selection. For example, Krishnakumar et al. [53] included an AI-based decision support system that offered real-time, data-driven feedback. Albino de Queiroz et al. [44] combined rule-based logic with natural language processing (NLP) and IoT (Internet of Things) integration to support symptom and lifestyle monitoring. Other studies such as Chaix et al. [46], Schlieter et al. [63], and Sweidan et al. [60], employed NLP (natural language processing) or intent-based architectures (e.g., Dialogflow) to classify user input and deliver tailored pre-scripted responses. These systems illustrated early-stage AI integration but still rely on predefined intents or decision trees, rather than fully generative or self-learning models. Notably, all studies incorporating AI features were published from 2019 onwards, highlighting the relatively recent shift toward integrating adaptive technologies into virtual agent-delivered interventions.

### Rationale for intervention design & elements

While research question 1 aimed to examine the rationale authors provided for the use or combinations of BCTs, health data, and delivery channels, most studies framed their justification at the level of the overall intervention design and purpose, which typically included a virtual agent as one component of a broader digital intervention. These rationales were typically stated in broad terms (e.g., improving adherence), rather than offering explicit reasoning for individual components. Authors often described why they created a particular intervention but rarely articulated why individual BCTs or technical delivery components were chosen or how they were theoretically or practically linked. As such, Table 3 presents the rationale category per study based on the overarching intervention design approach (*theory-driven, practice-driven, empirically-driven, not explicitly stated or mixed*), rather than specific justifications for particular BCTs, health data types, or delivery channels. Although some studies described aspects of the intervention development process (e.g., “functionalities iteratively developed with nurses”), these descriptions generally did not constitute explicit rationales for individual components. The distribution shows that n = 4 studies (19.0%) were theory-driven, n = 2 (9.5%) practice-driven, n = 3 (14.3%) empirically-driven, n = 8 (38.1%) reported mixed rationales, and n = 4 (19.0%) did not explicitly state a rationale.

**Table 3:** Intervention Design Rationales per study.

Study ID	Rationale (short summary)	Category
Baptista et al. [45]	Human-like engagement; draws on theories of therapeutic alliances from digital health (Bickmore, 2005)	Theory-driven
Gong et al. [49]	Design embedded in applied behavior change theories (including the transtheoretical model, social cognitive theory, gamification & BCTs)	Theory-driven
Krishnakumar et al. [53]	Based on AADE7 self-care behavior framework, a digital persuasion model & a gamified approach	Theory-driven
Schläpfer [58]	Based on behavior change theory in the context of stress & self-regulation (health action process approach, self-determination theory, BCTs & gamification) & clinical practice guidelines	Theory-driven
Huang et al. [52]	Review of health & hospital instruction materials; interviews with cancer managers, nurses & physicians	Practice-driven
Roca et al. [56]	Functionalities developed iteratively with nurses	Practice-driven
Hauser-Ulrich et al. [51]	Based on cognitive behavioral therapy & social support principles, built with open-source tech	Empirically-driven
Ter Stal et al. [43]	Designed to enhance trust & motivation in relational agent; agent characteristics based on previous literature	Empirically-driven
Zisis et al. [62]	Based on positive pilot study results on knowledge & self-care behaviors	Empirically-driven
Echeazarra et al. [47]	Based on previous general research on chatbots; chatbots chosen to reduce Health care professional workload	Empirically driven/ not explicitly stated
Gomaa et al. [48]	Based on qualitative patient interviews & identified needs; literature review	Practice & empirically-driven
Gómez et al. [50]	Based on user-centered/model-based design (MASAIK-M Framework); interface usability & decision-making support	Practice & Empirically-driven
Schlieter et al. [42]	Based on both literature review on requirements for artifact design (including the application of BCTs) & participatory design process (including clinicians, health-care staff & patients)	Practice & Empirically-driven
Wonggom et al. [61]	Participatory design process (patients, family members, clinical experts & IT specialists); Content entirely based on national heart foundation guidelines	Practice & Empirically-driven
Magnani et al. [54]	Based on literature & patient-domain review; qualitative interviews with arterial fibrillation patients	Practice & Theory-driven
Sakane et al. [57]	Developed by multidisciplinary team & focus groups, based on behavior change techniques and goal-setting theory	Practice & Theory-driven
Shamekhi et al. [59]	Based on principles of mindfulness-based stress reduction & adult learning; integrated monthly input of patient-advisory group	Practice & Theory-driven
Albino de Queiroz et al. [44]	Based on prior digital intervention research and scalability & engagement properties of AI & NLP	Not explicitly stated
Chaix et al. [46]	Mostly descriptive & exploratory	Not explicitly stated
Qiu et al. [55]	Algorithm for personalized content strategy described	Not explicitly stated
Sweidan et al. [60]	Tailored to older users’ needs & usability; design optimized for intuitiveness	Not explicitly stated

**Abbreviations:** AI = artificial intelligence; NLP = natural language processing

Only a small subset of studies (n = 3, 14.3%) discussed interconnections between BCTs, health data, and delivery channels [42,57,58]. Even among these, the reported links were often partial, focusing primarily on connections between BCTs and delivery channels, or between health data and delivery channels, rather than all three components simultaneously. An example is Schläpfer et al. [58], who explicitly reported each of their 39 BCTs applied in the intervention, and showed how each BCT was integrated within the intervention. Though the used delivery channels and health data could be inferred from the combined description of “app integration”, it was possible to deduce only one of these components per BCT, rather than both together. Still, while the overall intervention was grounded in theory (see Table 3), the rationale for selecting or combining specific BCTs, health data types, or delivery channels was not explicitly discussed.

Use of virtual agent-delivered BCT, health data & delivery channel combinations

While design rationales were usually framed at the intervention level, the reporting of specific intervention components was less consistent. Only n = 3 (14.3%) explicitly reported their use of BCTs, whereas the majority of studies incorporated BCTs without explicitly acknowledging them in their methodology or direct reference to standardized BCT terminology. Specifically, BCTs were inferred from the intervention descriptions in n = 18 (85.7%), typically through descriptions of functions (e.g., an intervention sending reminders to the patient without labelling it as a “Prompts/Cues” BCT). We synthesized the intervention elements (BCTs, health data & delivery channels) reported or inferred across all n = 21 (100%) included studies (see Table 4). The table highlights which components were delivered directly by the virtual agent in bold, as opposed to other parts/ modules of the digital system or intervention. Examples of how intervention features were mapped to BCTTv1 categories are provided in S2 Table.

**Table 4:** Study-specific details on BCTs, health data, and delivery channels*.

Study	Health Data Type (Input)	Health Data Type (Output)	BCTs Used	Delivery Channel	Delivery Mechanisms
Albino de Queiroz et al. [44]	Objective (steps & distance via smart tracker), subjective (diet, symptoms & adverse treatment effects), adherence data (time of use)	**Personalized feedback** on physical activity, **actionable recommendations** (based on symptom severity)	**Self-monitoring, feedback, prompts/cues**	Smartphone app, **chatbot via Facebook Messenger**	**Individualized messages based on self-report & wearable data, reminders to engage & report symptoms/ activities**
Baptista et al. [45]	Subjective (self-reported diabetes self-management practices), objective (EHR incl. glycated hemoglobin A1c & average glucose levels last 2–3 months from GP)	**Health feedback/ status** (on diet & exercise), **actionable recommendations**	**Self-monitoring, feedback, instruction on how to perform a behavior**, **social reward, prompts/cues, goal-setting**	**Smartphone app**	**Conversational interactions, reminders/push notifications**
Chaix et al. [46]	Subjective (self-reported medication adherence), user preference data (preferred timing of reminders), adherence data	**Health feedback & actionable recommendations** (medication reminders, educational messages on treatments, diet, exercise & Quality of Life)	**Self-monitoring, instruction on how to perform a behavior, prompts/cues**	**Smartphone app**	**Educational messages, instructions & reminders on medication intake & side-effect management**
Echeazarra et al. [47]	Objective (blood pressure), adherence data (whether BP was measured)	**Health feedback/status** (messages, visual graphs), **actionable recommendations** (videos with instructions)	**Self-monitoring, feedback, instruction on how to perform a behavior, prompts/cues**	BP Tensiometer & **Smartphone app (Telegram or Whatsapp)**	**Interface to view visual charts, chatbot feedback incl. graphs, messages & videos, reminders to measure blood pressure**
Gomaa et al. [48]	Subjective (self-reported symptom severity), objective (e.g., clinical baseline characteristics like diagnosis, stage, treatment regimen from medical records), adherence data (e.g., responses to symptom checks)	**Health feedback/status** (e.g., symptom severity feedback); **actionable recommendations** (personalized self-care recommendations, educational information about symptom management)	**Self-monitoring, feedback, instruction on how to perform a behavior,** prompts/cues	**Smartphone app with integrated chatbot** and separate text-messaging (SMS)	Interactive text messages offering text back keywords, **separate chatbot toxicity assessment with real-time feedback**
Gong et al. [49]	Objective data (blood glucose via Bluetooth meter), subjective data (self-reported behavior, knowledge through module quizzes & interactions), Adherence data (e.g., number of completed & total duration of chats, glucose level uploads and discussion forum posts)	**Health feedback/status** (progress summaries, glucose level feedback); **actionable recommendations** (tailored coaching messages, behavioral suggestions)	**Self-monitoring, feedback, instruction on how to perform a behavior,** social support, **problem-solving, information on health consequences**	**Smartphone app**; web-based discussion board forum; e-mail	**Conversational feedback, education, tips, short quizzes & counselling on chosen topic;** Web-based forum posts for discussions with participants; mail follow-up reminders
Gómez et al. [50]	Objective data (continuous glucose readings, insulin pump data); subjective data (self-reported dietary intake & events, symptoms, mood, behavior); adherence data (e.g., insulin pump usage data, timestamps, and duration logged by system.)	**Health feedback/status** (insulin therapy recommendations); **visual data** (e.g., glucose & insulin trend graphs, logbook view); **actionable recommendations** (e.g., therapy adjustments from clinicians, bolus recommendations from algorithm)	**Self-monitoring, feedback**	**Smart assistant (PDA) with wireless communication;** integrated telemedicine central server via GPRS for data synchronization & clinician updates; text-messaging (SMS) & e-mail notifications for remote advice;	**Smart assistant real-time feedback, visual summaries of glucose & insulin data, behavioral prompts via PDA interface, graphical visualization of glucose profiles/insulin dosages;** physician-to-patient recommendations via GPRS transmitted via PDA
Hauser-Ulrich et al. [51]	Subjective data (self-reported pain intensity to measure pain-related impairment later, mood), user preference data (chosen modules at baseline (3 from dysfunctional cognitions & behavior modules, 3 from coping strategies modules)	**Health feedback/status** (daily feedback & encouragement messages), **actionable recommendations** (behavioral coaching, coping strategies, relaxation exercises)	**Self-monitoring, feedback, instruction on how to perform a behavior, prompts/cues, reduce negative emotions**	**Smartphone app**	**Scripted chat interface with predefined answer options, embedded multimedia content (video, audio, PDFs), and daily notifications/reminders**
Huang et al. [52]	Subjective data (self-reported symptoms)	**Health feedback/status** (immediate symptom feedback); **actionable recommendations** (symptom relief suggestions if symptoms were mild)	**Self-monitoring, feedback, instruction on how to perform a behavior, prompts/cues,**	**Smartphone app (Facebook Messenger)**	**Notifications & symptom checks via structured questions**
Krishnakumar et al. [53]	Objective data (self-reported blood glucose meter data & weight)	**Health feedback/status** (real-time feedback on glucose, weight and activity); **visual data** (trend reports); **actionable recommendations** (tailored advice and behavior reinforcement through chatbot interactions)	**Self-monitoring, feedback**, **instruction on how to perform a behavior,** social support, **prompts/Cues**, **Problem-solving**, **reward (outcome)**	**Smartphone app**	**Real-time conversational feedback on user-logged data, educational/behavioral/ motivational messaging,** optional messaging and voice calls with diabetes instructors
Magnani et al. [54]	Objective data (ECG heart rhythm & rate, BMI, clinical covariates form EHR, medication regime); subjective data (self-reported symptoms), adherence data (relational agent date/time of use, duration, specific domains of content accessed)	Health feedback/status (tailored symptom feedback); actionable recommendations (specific recommendations on symptom management, medication adherence & contacting HCP based on symptom reports and Kardia data)	**Self-monitoring**, **feedback, instruction on how to perform a behavior,** goal-setting (behavioral)	**Smartphone app**	Daily selection of topics by users via touchscreen, **content delivered in conversations with agent (symptom reporting/selection via touchscreen, instructional & educational support)**
Qiu et al. [55]	Objective data (Step counts reported by participant but collected via pedometers); Subjective Data (Self-reported pain, sleep, distress, fatigue)	**Health feedback/status** (daily tailored intervention suggestions based on symptom/activity data), visual data (illustrations of pain scales & symptom trends), **actionable recommendations** (tailored suggestions for physical activity, relaxation exercises, nutrition tips, symptom self-management)	Self-monitoring, feedback, shaping knowledge (Instruction on how to perform a behavior), **prompts/Cues**	Amazon Echo Show (**smart speaker** with screen), Touch-Screen interaction for menu navigation	**Daily structured conversational prompts via smart speaker**, videos for exercise instruction, meditation audio, educational videos for coping strategies
Roca et al. [56]	Objective data (HbA1c lab results, medication possession ratio at baseline and after 9 months), Subjective data (Patient self-reported medication adherence, depressive symptoms) adherence data (System logs capturing reminder responses, frequency of use, functionality assessed)	**Health feedback/status** (Weekly adherence summaries), **actionable recommendations** (medication reminders, appointment notifications)	**Self-monitoring, feedback, prompts/cues**	**Smartphone app (Signal)**	**Push notifications & reminders, manual logging via chatbot interface, chatbot message summaries**
Sakane et al. [57]	*Input:* Objective data (weight via Bluetooth scale, steps via pedometer, blood pressure via BP monitor, health check-up data), Subjective data (behavioral agenda target like habits or steps, goal-setting data, self-reported diet, exercise and lifestyle habits, personality traits), adherence data (app use data including frequency of self-weighing, pedometer use, BP measurements & quiz completion)	**Health feedback/status** (chatbot feedback on weight trends, step counts, BP trends), **actionable recommendations** (specific health guidance tailored to patient assessments), **engagement reinforcement** (chatbot delivered quiz on health and behavior)	**Self-monitoring, feedback, instruction on how to perform a behavior,** goal-setting	**Smartphone app**	**Chatbot-delivered health-guidance, feedback & quiz**
Schläpfer et al. [58]	Subjective data (Self-reported distress levels, self-efficacy scores, reported relaxation practices), adherence data (app use logs, exercise completion, coaching sessions)	Health Feedback/Status (through dashboard, **personalized coaching messages**, tailored weekly messages)	39 BCTs incorporated; key BCTs delivered by chatbot: **goal-setting (behavioral & outcome), problem-solving, action planning, review behavioral goals, discrepancy between current behavior and goal, positive reframing, normalize difficulty, emphasize autonomy**	**Smartphone app**	**Personalized weekly coaching messages,** relaxation exercises, tailored reminder notifications, educational video clips, personalized in-app letters
Schlieter et al. [42]	Objective data (step counts, heart rate, motion tracking via sensors), Subjective data (self-reported progress & health status)	Health feedback/status (Personalized feedback on performance & adherence), visual data (display of progress & trends), actionable recommendations (adaptive suggestions for exercise & learning content)	**Self-monitoring, feedback, instruction on how to perform a behavior, prompts/cues**	**Tablet-app**	**Interactive avatar, gamified interface, scheduled prompts, adaptive coaching pathways**
Shamekhi et al. [59]	Subjective data (self-reported mood, physical comfort, goals), adherence data (login frequency, practice progress), user preference data (content and goals review history)	**Health feedback/status** (adjusted session content based on mood, comfort, prior review, or login time), **actionable recommendations** (meditation, yoga, education)	**Self-monitoring, goal-setting (behavior), instruction on how to perform a behavior, adding objects to the environment**	**Tablet-app**	**Scripted dialogue, multimedia content (video, audio, visuals)**
Ter Stal et al. [43]	Objective data (weight from smart scale, physical activity from Fitbit, inhalation use & technique), subjective data (daily symptom diary), adherence data (in-app log data)	**Health feedback/status** (symptom diary results), **actionable recommendations** (self-management advice)	Self-monitoring, **feedback, prompts/cues,** adding objects to the environment, Instruction on how to perform a behavior, Goal-setting (behavior)	**Tablet-app**	**System-triggered & user-triggered visual dialogues,** dashboard with log data/ goals, overview & access to videos/ informational material
Sweidan et al. [60]	Subjective data (self-reported completion of daily tasks, medication intake), Adherence data (logged medication intake, app use frequency & task completion logs), contextual data (self-reported location sharing for emergency situations)	Health feedback/ status (configurable notifications such as medication reminders, completion of daily tasks, **provision of information about disease**)	Self-monitoring, prompts/cues, **instruction on how to perform a behavior**, social support	**Smartphone app**	Multiple app modules (test & games, daily activities, chat with doctor/caregiver, personal patient archive, **about Alzheimer knowledge chatbot**)
Wonggom et al. [61]	Subjective data (self-reported quiz knowledge & self-care behavior assessment)	**Health feedback/ status** (Quiz scores reflecting knowledge levels), **actionable recommendations** (educational content related to disease & self-care behaviors based on quiz responses)	**Instruction on how to perform a behavior, prompts/cues**	**Tablet-app**	Structured heart failure self-care education content through scripted videos and animations
Zisis et al. [62]	Objective data (daily weight), subjective data (self-reported answers to HF knowledge, self-care behaviour, quality of life, anxiety, depression, and cognition questionnaire)	**Actionable recommendations** (feedback of when to contact clinician based on weight gain), **general HF education**	**Self-monitoring, prompts/cues, instruction on how to perform a behavior**	**Smartphone app**	**Manual data entry via to-do tasks, prompts for tasks like weighing**

* Virtual agent delivered intervention elements are displayed in bold font

interpretation of the cross-study patterns described below, a synthesis summary of virtual agent-delivered intervention features is provided in Table 5.

**Table 5:** Synthesis summary of virtual agent-delivered intervention features.

BCT	Health data	Virtual Agent Operationalization	Delivery Channel & Mechanism
Self-monitoring	Subjective data (symptoms, mood, behaviors); objective data (steps, weight, blood pressure)	Conversational check-ins or structured questions, asking users to log the data	Smartphone or tablet apps (chat interface)
Feedback on behavior	Subjective data (symptoms, mood, behaviors); objective data (steps, weight, blood pressure)	Chatbot-generated summaries or responses based on trends of the data	Smartphone or Tablet apps (push notifications or weekly updates)
Instruction on how to perform a behavior	Subjective data (user-entered symptoms, mood, behaviors or questions)	Educational content, tips, or action plans	Smartphone or Tablet apps (chat interface, scripted educational messages, multimedia audio or video clips)
Prompts/cues	Subjective data (symptom and mood logs, skipped entries); objective data (step count thresholds or inactivity): adherence or user preference data (completion of scheduled actions, preferred reminder timing)	Reminders or conversational check-ins based on prior data entries to log behavior or perform tasks	Smartphone or tablet apps or smart speakers (push notifications)

All interventions implemented at least one BCT via the virtual agent. **Self-monitoring** was one of the most frequent, used in n = 16 studies (76.2%). Agents supported users in logging subjective data inputs, such as symptom check-ins, mood and behavior, but also objective data inputs such as step count, weight, or blood pressure. These inputs were typically collected through conversational check-ins or structured questions. Delivery channels included mobile or tablet apps with embedded chat interfaces. In some studies, wearable-linked data was integrated into the agent’s responses, although data visualization occurred outside the agent interface. Those interventions that did not implement **self-monitoring** via the agent itself, delivered it via overview components like dashboards, where users could access their data. Next to this, **feedback on behavior**, which was used in n = 14 studies (66.7%), was often provided in response to self-monitored data. Feedback included chatbot-generated summaries, or responses based on trends in subjective or objective health data. Delivery mechanisms involved notifications, or weekly updates.

**Instruction on how to perform a behavior** was present in n = 15 studies (71.4%). Agents offered educational content, tips, or action plans to help users adopt or maintain healthy behaviors (e.g., diet, exercise, symptom relief). Health data used to tailor these instructions mostly included subjective user-entered concerns, questions or symptom reports. This was typically delivered via mobile and tablet apps, with the agent presenting instructions through conversational modules, scripted educational messages, and in some cases, multimedia such as videos or audio clips.

Additionally, **prompts and cues** were used in n = 13 studies (61.9%), typically to remind users to complete self-monitoring tasks or engage in healthy behaviors. Agents initiated push notifications, reminders, or daily check-in messages based on prior data entries. Prompts were linked to both subjective data (e.g., symptom and mood logs, skipped entries) and objective data (e.g., step count thresholds or inactivity), but also adherence or user preference data input (e.g., completion of scheduled actions, preferred timing of reminders). Delivery was commonly facilitated through mobile or tablet apps and messaging platforms such as Telegram and Facebook Messenger, and once also per smart speaker in Qiu et al. [55], suggesting a flexible use of this technique across different data modalities and delivery infrastructures. Prompts and cues played a central role in sustaining engagement and encouraging consistent user interaction.

Other techniques, such as **goal-setting, social support**, **reward**, and **problem-solving**, were delivered by agents in only n = 6 studies (28.6%), usually in systems capable of more adaptive or personalized conversation. These still included rule-based systems as in Schläpfer et al. [58], but also AI-enhanced or hybrid systems like those used in Krishnakumar et al. [53]. Agents helped users formulate or review goals, receive social encouragement, earn symbolic rewards and navigate barriers, within conversational dialogue delivered mostly via smartphone apps. No consistent pattern emerged in the use of health data tied to these BCTs.

While some delivery channels such as smartphone based messaging platforms (e.g., Telegram or Facebook Messenger) or smart speakers (e.g., Amazon Echo Show) supported agent delivery directly, most interventions relied on dedicated smartphone or tablet apps where the virtual agent was embedded as one component within a broader intervention system. The identified agent-delivered combinations set the foundation for the subsequent analysis of their frequency across conditions and context (Objective 3).

### Patterns & contexts of virtual agent-delivered BCTs, health data types & delivery channel combinations

Building on the agent-delivered BCT, health data, and delivery channel combinations identified in Objective 2 (see Tables 4 and 5), this section explores the contexts in which these combinations were applied, including chronic condition focus, intervention aim and setting. Because all interventions implemented multiple BCTs simultaneously, the clusters described below are not mutually exclusive, and individual studies may appear in more than one cluster. The operationalization of these combinations, including the types of health data used and delivery mechanisms, is summarized in Table 6.

**Table 6:** Patterns & contexts of BCT-Health data-delivery channel combinations.

BCTs	Health Data	Delivery Channel	Delivery Mechanism	Common Contexts	Prevalence	Studies
Self-monitoring + Feedback	Objective (e.g., step count, blood pressure), Subjective (e.g., symptoms, behavior), adherence (e.g., number of completed chats, glucose level uploads)	Smartphone/tablet apps	Chat dialogue, dashboards, visual summaries	Home-based, focus on self-management, symptom tracking, or adherence (diabetes, cancer, hypertension, chronic pain)	n=13 (61.9%)	[42,44,45,47–54,56,57]
Self-monitoring + Feedback + Instruction on how to perform a behavior	Objective (e.g., blood pressure, weight), Subjective (e.g., diet, habits, symptoms), adherence data	Smartphone/ Tablet apps	Chat dialogue, educational modules, multimedia (video/audio/ quizzes)	Home-based, focus on self-management, symptom tracking or adherence, rehabilitation (diabetes, cancer, hypertension, chronic pain)	n=10 (47.6%)	[42,45,47–49,51–54,57]
Self-monitoring + Feedback + Prompts/Cues	Objective (e.g., blood pressure, activity), Subjective (e.g., mood, symptoms), adherence data, user preference data	Smartphone/Tablet apps	Chat dialogue, Push notifications, reminders, daily-check in messages	Home-based, focus on self-management, symptom tracking or adherence (diabetes, cancer, hypertension, chronic pain)	n=8 (38.1%)	[42,44,45,47,51–53,56]
Instruction on how to perform a behavior	Subjective (self-reported knowledge, educational progress)	Smartphone/Tablet apps	Scripted avatar-based videos, chatbot-based lessons	Home-based support for Alzheimer’s or heart failure patients, focus on caregiver/patient education & self-care	n=2 (9.5%)	[60,61]

A closer look at the health data suggests tentative condition-specific patterns across the interventions. Cancer or chronic pain studies (n = 8, 38.1%) relied more heavily on subjective user-centred data (e.g., symptoms, side-effects, mood), with objective data (e.g., step counts) adherence data (e.g., exercise completion) and/or user preference data (e.g., preferred timing of reminders) as additional inputs layered on top. By contrast, cardiovascular and metabolic studies more often combined subjective data on self-management behaviors and objective physiological measures (e.g., blood pressure, weight, glucose etc.), supplemented with adherence data (e.g., measurement completion). Beyond this distinction, no consistent pattern emerged linking specific BCT-data-delivery combinations to particular conditions or system types.

## Discussion

This systematic scoping review mapped how BCTs, health data types, and delivery channels were rationalized, combined, and applied in virtual agent-delivered interventions for chronic condition management across 21 studies. While the analysis of design rationales provided contextual insight into how authors justified intervention design choices, the structured extraction of BCTs, health data, and delivery channels allowed a complementary mapping of how these components were operationalized within the interventions.

The findings highlight several patterns and gaps that have implications for both research and design of digital health interventions in the chronic care context: First, the rationale behind choosing and combining BCTS, health data, and digital delivery channels was rarely explained (see Table 2). Second, BCTs self-monitoring and feedback emerged as central to virtual agent-delivered interventions, combined with either instruction on how to perform a behavior or prompts/cues (see Tables 4 and 5). Prominent types of health data used in conjunction were both user-centered subjective and objective data, next to adherence and user-preference data logs, commonly delivered via smartphone or tablet apps, through conversational dialogue, notification systems, or pre-scripted intervention modules. Third, in a subset of studies, these elements appeared to be more tightly integrated, suggesting a shift towards more adaptive and cohesive interventions.

Previous reviews have examined conversational agents in healthcare and their use of behavior change techniques. For example, Martinengo et al. [29] reviewed conversational agent interventions across a broad range of health domains and identified commonly used BCTs, while Jiang et al. [12] focused on the development and evaluation characteristics of ECAs for chronic diseases. In contrast, the present review focuses specifically on virtual agent interventions for physical chronic conditions and extends prior work by systematically examining how health data inputs are combined with BCTs and delivery channels within intervention architectures.

### Current status

#### Lack of design rationales.

Most design rationale justifications focused on the overall intervention purpose (e.g., behavior change) without offering specific reasoning for individual components or how they were intended to interact. Our categorization confirmed that while some interventions were shaped by participatory design, prior research, or referenced behavioral theories, they still lacked transparent or structured justification for their design choices at the level of individual components. This observation aligns with earlier review findings of Martinengo et al. [29], who noted that only 26% of the conversational agent delivered interventions in health care settings they extracted were informed by a behavior change theory. However, in our review, we found that even when theories are mentioned, they were not typically operationalized in a detailed or component-level manner. This lack of transparent rationales resonates with broader observations in digital health design. Voorheis et al. [64] reported that digital health design often features fragmented and inconsistent application of behavioral frameworks in real-world development processes, as design leaders use them less as prescriptive solutions but rather as flexible reference points shaped by factors such as team expertise, intervention complexity, and ease of access to behavioral science knowledge. Our finding that many interventions fell into a single rationale category (e.g., empirically or practice-driven) may reflect this reality, where narrow disciplinary focus drives decisions, even though digital health interventions benefit from integrating diverse forms of knowledge and expertise across domains.

An important methodological similarity is that most BCTs in both this and prior reviews were not explicitly labeled using standardized taxonomies like the BCCT v1. Like Martinengo et al. [29],we inferred many BCTs from intervention descriptions, highlighting a continued challenge for transparent and reproducible reporting in this field.

#### Consistent patterns in applied intervention components.

Across studies, a consistent set of BCTs emerged as central to agent-delivered interventions: self-monitoring, feedback, instruction on how to perform a behavior, and prompts and cues. This pattern suggests interventions to commonly be built around a “monitor-act-reinforce” logic wherein users provide data, receive timely feedback or guidance, and are reminded to sustain behaviors over time. Notably, other techniques (e.g., problem-solving) appeared less often, but tended to be layered on top of these foundational BCTs in more adaptive interventions. Our findings extend those of Martinengo et al. [29], who also identified a core set of 2–6 BCTs in conversational agent interventions targeting chronic disorder management and lifestyle change. By documenting how other (also partly more complex) techniques are appearing in layered combinations within adaptive systems, our review highlights an emerging shift towards richer and more cohesive intervention strategies. This shift is also meaningful in light of evidence from Eaton et al. [30] who observed that featuring a greater number and diversity of BCTs tend to show greater benefit. However, our findings suggest that not only the number of BCTs, but also the way they are combined and embedded within system interactions may matter. In many of the reviewed studies, BCTs such as feedback, instruction on how to perform a behavior and prompts/cues were integrated within a single interaction flow, indicating that these elements may operate synergistically rather than as isolated components.

Beyond the prevalence of specific techniques, our review also revealed how intervention components were increasingly integrated. Across studies, feedback and Instruction on how to perform a behavior often served dual roles: not only as BCTs but also as delivery mechanisms for presenting health data output to users. This blurring of behavioral and functional boundaries reflects the growing sophistication of virtual agent systems, where behavior change strategies like BCTs and system functionalities are not siloed, but embedded within a cohesive interaction flow. This raises questions about whether BCTs should be conceptualized as discrete intervention components or as integrated interaction processes within digital systems, where multiple behavioral strategies may be experienced simultaneously by users. For example, in Krishnakumar et al. [53], AI-driven personalization enabled agents to combine self-monitoring with real-time conversational feedback on user-logged data, and tailored educational and motivational messaging using advanced BCTs like problem-solving within a single mobile interface. Schlieter et al. [42] illustrated how an ECA could integrate feedback, instruction on how to perform a behavior, and adaptive coaching into a unified interactive environment, while Schläpfer et al. [58] demonstrated that even a rule-based system can incorporate complex techniques such as problem-solving. Together, these examples demonstrate a tentative trend linking AI-enhancement to greater BCT complexity, but also show that advanced techniques can be achieved within structured, rule-based designs. Some tentative condition-specific patterns also emerged (e.g., cancer and pain studies relied more heavily on subjective data, whereas cardiovascular and metabolic studies combined subjective and objective measures), though no consistent evidence-based design strategies could be established from this. This suggests that beyond improving reporting, the field should move toward systematically examining how combinations of BCTs, health data, and delivery channels function across different chronic care contexts. Such work would help inform more transparent and evidence-based virtual agent intervention design, while still allowing room for contextual adaptation and design innovation. Rather than identifying fixed design “recipes”, this may support the development of mechanism-level insights that can be adapted and refined to specific target groups and settings.

#### Broader developments and future directions in virtual agent design.

These findings mirror broader developments in the field of digital health, where increasing attention is given to personalization and adaptivity. Uetova et al. [26] emphasized the increasing variability in personalization and the use of medical records in conversational agents, highlighting the need for agents to dynamically adapt to evolving patient needs. Craig et al. [24] similarly noted that many digital behavior change interventions are designed as integrated systems - combining data collection, AI-driven decision-making, and specific BCTs - yet the lack of transparent reporting practices makes it difficult to determine which components of combinations drive outcomes. As generative AI becomes more widely used, these distinctions between BCTs, data inputs, and delivery modes may further dissolve, paving the way for more fluid, responsive interaction models. This makes it even more important to study how these combinations function together, since their roles may overlap in practice, but still drive different outcomes. Encouragingly, recent protocols such as the CARE-ON trial by Goevarts et al. [65], illustrate efforts to strengthen transparency and theory-informed planning, although results are still pending. To support such developments, future work should adopt standardized taxonomies like the BCTTv1 and provide clear links between intervention components. At the same time, future research may benefit from approaches that examine how behavioral strategies interact with system functionalities and health data flows within digital health systems, rather than treating individual techniques as isolated intervention elements. Developing visual or modular mapping frameworks may further support cross-study comparison and methodological clarity. The recently proposed AI Impact Communications Model (AI-ICM) by Weingott and Parkinson [66], offers a conceptual foundation for aligning AI functionalities with behavioral communication theory. This model outlines how AI features can be mapped to communication constructs like “message framing”, offering a roadmap for the design of theoretically informed and replicable AI-driven behavior change tools. The findings of this review support the relevance of such approaches and underscore the importance of explicitly linking adaptive system functions to behavioral theory in future virtual agent interventions.

### Strengths and limitations

To our knowledge, this review is the first to systematically map the use of behavior change techniques, health data, and delivery channels in virtual agent-delivered interventions specifically targeting chronic condition management. By using a structured data extraction process grounded in the BCT taxonomy and scoping review guidelines (e.g., PRISMA-ScR), the review offers a comprehensive and reproducible synthesis. Second, the analysis not only identifies the presence of these components but also highlights patterns in how they are combined – offering insights into emerging design trends such as BCT layering and AI-enhanced personalization. Third, the review contributes to methodological transparency by explicitly addressing gaps in rationale reporting and proposing how future studies could describe how intervention elements interact. Together, these strengths enhance the review’s relevance for both researchers and digital health developers aiming to build more theoretically grounded and adaptable virtual agent interventions.

Nevertheless, several limitations should be acknowledged. Due to inconsistent reporting across studies, much of the coding for BCTs and delivery mechanisms relied on interpretive synthesis. In particular, BCTs in the category shaping knowledge were frequently inferred and coded as “instruction on how to perform a behavior”, given the absence of a distinct BCT for general health or condition-specific information provision. Because many studies did not explicitly label BCTs, coding relied on mapping intervention descriptions to BCTTv1 definitions, which may have influenced the frequency with which certain techniques were identified. Additionally, it was not always possible to distinguish which components were delivered by the agent versus the broader system, especially in older or hybrid designs. The included studies also varied widely in target conditions, intervention complexity, and reporting style, which limited direct compatibility. Because the number of studies per chronic condition was limited, the patterns identified in this review should be interpreted cautiously. Finally, the search was restricted to peer-reviewed publications and did not include gray literature, which may have excluded developments reported in industry reports or non-academic sources. Taken together, these challenges reflect a broader lack of reporting standards in digital health trials and highlight the need for more transparent and detailed intervention descriptions.

## Conclusion

This review demonstrates that while virtual agent interventions for chronic care increasingly incorporate behavior change techniques, health data, and digital delivery channels, there remains a notable gap in how these elements are rationalized and integrated. To our knowledge, we provide the first systematic map of BCT-health data-delivery channel combinations in virtual agents delivered chronic care interventions, identifying patterns in their design. Foundational BCTs like self-monitoring, feedback, instruction on how to perform a behavior and prompts and cues were most commonly observed across virtual agent-delivered interventions, while more complex techniques and adaptive architectures appeared in a subset of studies, particularly in more adaptive or AI-enhanced systems. To support transparency and reproducibility in future research, we recommend that intervention studies explicitly report BCTs using standardized taxonomies such as the BCTTv1, clearly describe how health data are used to tailor virtual agent interactions, and provide structured descriptions of how BCTs, health data inputs, and delivery channels are integrated within the virtual agent architecture. Such reporting would facilitate comparison across studies and support the development of more evidence-informed design strategies for different chronic conditions.

## Supporting information

S1 Checklist PRISMA checklist. (DOCX)

S1 Text Registered study protocol. (DOCX)

S2 Text Search string. (DOCX)

S1 Table Data extraction template. (DOCX)

S2 Table BCT coding evidence. (DOCX)

## References

[1] World Health Organization. Noncommunicable diseases [Internet]. 2024 [cited 2025 Sep 4]. Available from: https://www.who.int/news-room/fact-sheets/detail/noncommunicable-diseases

[2] Debon R, Coleone JD, Bellei EA, De Marchi ACB. Mobile health applications for chronic diseases: A systematic review of features for lifestyle improvement. Diabetes Metab Syndr. 2019;13(4):2507–12. doi: 10.1016/j.dsx.2019.07.016 31405669

[3] Braillard O, Slama-Chaudhry A, Joly C, Perone N, Beran D. The impact of chronic disease management on primary care doctors in Switzerland: a qualitative study. BMC Fam Pract. 2018;19(1):159. doi: 10.1186/s12875-018-0833-3 30205832 PMC6134721

[4] Mair JL, Salamanca-Sanabria A, Augsburger M, Frese BF, Abend S, Jakob R, et al. Effective Behavior Change Techniques in Digital Health Interventions for the Prevention or Management of Noncommunicable Diseases: An Umbrella Review. Ann Behav Med. 2023;57(10):817–35. doi: 10.1093/abm/kaad041 37625030 PMC10498822

[5] Viderman D, Seri E, Aubakirova M, Abdildin Y, Badenes R, Bilotta F. Remote monitoring of chronic critically ill patients after hospital discharge: a systematic review. J Clin Med. 2022;11(4). doi: 10.3390/jcm11041010 PMC8879658 35207287

[6] Philip P, Dupuy L, Morin CM, de Sevin E, Bioulac S, Taillard J. Smartphone-based virtual agents to help individuals with sleep concerns during COVID-19 confinement: feasibility study. J Med Internet Res. 2020;22(12). doi: 10.2196/24268 33264099 PMC7752183

[7] Li Y, Liang S, Zhu B, Liu X, Li J, Chen D, et al. Feasibility and effectiveness of artificial intelligence-driven conversational agents in healthcare interventions: A systematic review of randomized controlled trials. Int J Nurs Stud. 2023;143:104494. doi: 10.1016/j.ijnurstu.2023.104494 37146391

[8] Kramer L, Ter Stal S, Mulder B, De Vet E, Van Velsen L. Developing embodied conversational agents for coaching people in a healthy lifestyle: scoping review. J Med Internet Res. 2020;22(2):e14058. doi: 10.2196/14058 PMC7055763 32022693

[9] Rampioni M, Stara V, Felici E, Rossi L, Paolini S. Embodied Conversational Agents for Patients With Dementia: Thematic Literature Analysis. JMIR Mhealth Uhealth. 2021;9(7):e25381. doi: 10.2196/25381 34269686 PMC8325086

[10] Maher CA, Davis CR, Curtis RG, Short CE, Murphy KJ. A Physical Activity and Diet Program Delivered by Artificially Intelligent Virtual Health Coach: Proof-of-Concept Study. JMIR Mhealth Uhealth. 2020;8(7):e17558. doi: 10.2196/17558 32673246 PMC7382010

[11] Lyzwinski LN, Elgendi M, Menon C. Conversational Agents and Avatars for Cardiometabolic Risk Factors and Lifestyle-Related Behaviors: Scoping Review. JMIR Mhealth Uhealth. 2023;11:e39649. doi: 10.2196/39649 37227765 PMC10251225

[12] Jiang Z, Huang X, Wang Z, Liu Y, Huang L, Luo X. Embodied Conversational Agents for Chronic Diseases: Scoping Review. J Med Internet Res. 2024;26:e47134. doi: 10.2196/47134 38194260 PMC10806449

[13] Sheldon R, HPC, & BB. What is a virtual agent? TechTarget [Internet]. 2024 [cited 2024 Aug 1]. Available from: https://www.techtarget.com/searchcustomerexperience/definition/virtual-agent

[14] Friedman RH, Frank AD. Use of conditional rule structure to automate clinical decision support: a comparison of artificial intelligence and deterministic programming techniques. Comput Biomed Res. 1983;16(4):378–94. doi: 10.1016/0010-4809(83)90061-7 6352165

[15] Bindra S, Jain R. Artificial intelligence in medical science: a review. Ir J Med Sci. 2024;193(3):1419–29. doi: 10.1007/s11845-023-03570-9 37952245

[16] Martinengo L, Lin X, Jabir AI, Kowatsch T, Atun R, Car J, et al. Conversational Agents in Health Care: Expert Interviews to Inform the Definition, Classification, and Conceptual Framework. J Med Internet Res. 2023;25:e50767. doi: 10.2196/50767 37910153 PMC10652195

[17] Ennab M, Mcheick H. Enhancing interpretability and accuracy of AI models in healthcare: a comprehensive review on challenges and future directions. Front Robot AI. 2024;11:1444763. doi: 10.3389/frobt.2024.1444763 39677978 PMC11638409

[18] Alkhanbouli R, Matar Abdulla Almadhaani H, Alhosani F, Simsekler MCE. The role of explainable artificial intelligence in disease prediction: a systematic literature review and future research directions. BMC Med Inform Decis Mak. 2025;25(1):110. doi: 10.1186/s12911-025-02944-6 40038704 PMC11877768

[19] Hwang M, Jiang Y. Personalization in digital health interventions for older adults with cancer: A scoping review. J Geriatr Oncol. 2023;14(8):101652. doi: 10.1016/j.jgo.2023.101652 37866009

[20] Kankanhalli A, Xia Q, Zhao X. Understanding Personalization for Health Behavior Change Applications: A Review and Future Directions. THCI. 2021;:316–49. doi: 10.17705/1thci.00152

[21] Paper W, Shapiro M, Johnston D, Wald J, Mon D. Patient-Generated Health Data. RTI International; 2012.

[22] Montoliu T, Hidalgo V, Salvador A. Importance of Personality for Objective and Subjective-Physical Health in Older Men and Women. Int J Environ Res Public Health. 2020;17(23):8809. doi: 10.3390/ijerph17238809 33260870 PMC7729813

[23] Viljanen A, Salminen M, Irjala K, Heikkilä E, Isoaho R, Kivelä S-L, et al. Subjective and objective health predicting mortality and institutionalization: an 18-year population-based follow-up study among community-dwelling Finnish older adults. BMC Geriatr. 2021;21(1):358. doi: 10.1186/s12877-021-02311-w 34112108 PMC8193868

[24] Thomas Craig KJ, Morgan LC, Chen C-H, Michie S, Fusco N, Snowdon JL, et al. Systematic review of context-aware digital behavior change interventions to improve health. Transl Behav Med. 2021;11(5):1037–48. doi: 10.1093/tbm/ibaa099 33085767 PMC8158169

[25] Taylor KS, Umeukeje EM, Santos SR, McNabb KC, Crews DC, Hladek MD. Context Matters: A Qualitative Synthesis of Adherence Literature for People on Hemodialysis. Kidney360. 2023;4(1):41–53. doi: 10.34067/KID.0005582022 36700903 PMC10101575

[26] Uetova E, Hederman L, Ross R, O’ Sullivan D. Exploring the characteristics of conversational agents in chronic disease management interventions: A scoping review. Digit Health. 2024;10:20552076241277692. doi: 10.1177/20552076241277693 PMC11526412 39484653

[27] Haberer JE, Garrison L, Tumuhairwe JB, Baijuka R, Tindimwebwa E, Tinkamanyire J, et al. Factors affecting the implementation of electronic antiretroviral therapy adherence monitoring and associated interventions for routine HIV care in Uganda: qualitative study. J Med Internet Res. 2020;22(9):e18038. doi: 10.2196/18038 32687473 PMC7516683

[28] Michie S, Richardson M, Johnston M, Abraham C, Francis J, Hardeman W, et al. The Behavior Change Technique Taxonomy (v1) of 93 Hierarchically Clustered Techniques: Building an International Consensus for the Reporting of Behavior Change Interventions. Ann Behav Med. 2013;46(1):81–95. doi: 10.1007/s12160-013-9486-6 23512568

[29] Martinengo L, Jabir AI, Goh WWT, Lo NYW, Ringo Ho MH, Kowatsch T, et al. Conversational agents in health care: scoping review of their behavior change techniques and underpinning theory. J Med Internet Res. 2022;24(10):e39243. doi: 10.2196/39243 36190749 PMC9577715

[30] Eaton CK, McWilliams E, Yablon D, Kesim I, Ge R, Mirus K, et al. Cross-Cutting mHealth Behavior Change Techniques to Support Treatment Adherence and Self-Management of Complex Medical Conditions: Systematic Review. JMIR Mhealth Uhealth. 2024;12:e49024. doi: 10.2196/49024 38717433 PMC11085043

[31] Salas-Groves E, Galyean S, Alcorn M, Childress A. Behavior Change Effectiveness Using Nutrition Apps in People With Chronic Diseases: Scoping Review. JMIR Mhealth Uhealth. 2023;11:e41235. doi: 10.2196/41235 36637888 PMC9883741

[32] Webb TL, Joseph J, Yardley L, Michie S. Using the internet to promote health behavior change: a systematic review and meta-analysis of the impact of theoretical basis, use of behavior change techniques, and mode of delivery on efficacy. J Med Internet Res. 2010;12(1):e4. doi: 10.2196/jmir.1376 20164043 PMC2836773

[33] Thomas PC, Curtis K, Potts HWW, Bark P, Perowne R, Rookes T, et al. Behavior Change Techniques Within Digital Interventions for the Treatment of Eating Disorders: Systematic Review and Meta-Analysis. JMIR Ment Health. 2024;11:e57577. doi: 10.2196/57577 39088817 PMC11327638

[34] Arksey H, O’Malley L. Scoping studies: towards a methodological framework. Int J Soc Res Methodol. 2005;8(1):19–32. doi: 10.1080/1364557032000119616

[35] Tricco AC, Lillie E, Zarin W, O’Brien K, Colquhoun H, Kastner M, et al. A scoping review on the conduct and reporting of scoping reviews. BMC Med Res Methodol. 2016;16:15. doi: 10.1186/s12874-016-0116-4 26857112 PMC4746911

[36] Peters MDJ, Marnie C, Tricco AC, Pollock D, Munn Z, Alexander L, et al. Updated methodological guidance for the conduct of scoping reviews. JBI Evid Synth. 2020;18(10):2119–26. doi: 10.11124/JBIES-20-00167 33038124

[37] Tudor Car L, Dhinagaran DA, Kyaw BM, Kowatsch T, Joty S, Theng Y-L, et al. Conversational Agents in Health Care: Scoping Review and Conceptual Analysis. J Med Internet Res. 2020;22(8):e17158. doi: 10.2196/17158 32763886 PMC7442948

[38] Tricco AC, Lillie E, Zarin W, O’Brien KK, Colquhoun H, Levac D, et al. PRISMA Extension for Scoping Reviews (PRISMA-ScR): Checklist and Explanation. Ann Intern Med. 2018;169(7):467–73. doi: 10.7326/M18-0850 30178033

[39] Hoffmann TC, Glasziou PP, Boutron I, Milne R, Perera R, Moher D. Better reporting of interventions: Template for intervention description and replication (TIDieR) checklist and guide. BMJ (Online). 2014;348:g1687. doi: 10.1136/bmj.g1687 24609605

[40] Workman A, Johnston FH, Campbell SL, Williamson GJ, Lucani C, Bowman DMJS. Evaluating user preferences, comprehension, and trust in apps for environmental health hazards: qualitative case study. JMIR Form Res. 2022;6(12):e38471. doi: 10.2196/38471 PMC9816954 36548030

[41] Elo S, Kyngäs H. The qualitative content analysis process. J Adv Nurs. 2008;62(1):107–15. doi: 10.1111/j.1365-2648.2007.04569.x 18352969

[42] Schlieter H, Gand K, Weimann TG, Sandner E, Kreiner K, Thoma S, et al. Designing virtual coaching solutions: Design and evaluation of a digital health intervention for rehabilitation. Bus Inform Syst Eng. 2024;66(3):377–400. doi: 10.1007/s12599-024-00871-w

[43] Ter Stal S, Sloots J, Ramlal A, Op den Akker H, Lenferink A, Tabak M. An Embodied Conversational Agent in an eHealth Self-management Intervention for Chronic Obstructive Pulmonary Disease and Chronic Heart Failure: Exploratory Study in a Real-life Setting. JMIR Hum Factors. 2021;8(4):e24110. doi: 10.2196/24110 34734824 PMC8603169

[44] Albino de Queiroz D, Silva Passarello R, Veloso de Moura Fé V, Rossini A, Folchini da Silveira E, Aparecida Isquierdo Fonseca de Queiroz E, et al. A wearable chatbot-based model for monitoring colorectal cancer patients in the active phase of treatment. Healthc Anal. 2023;4:100257. doi: 10.1016/j.health.2023.100257

[45] Baptista S, Wadley G, Bird D, Oldenburg B, Speight J, My Diabetes Coach Research Group. Acceptability of an Embodied Conversational Agent for Type 2 Diabetes Self-Management Education and Support via a Smartphone App: Mixed Methods Study. JMIR Mhealth Uhealth. 2020;8(7):e17038. doi: 10.2196/17038 32706734 PMC7407258

[46] Chaix B, Bibault J-E, Pienkowski A, Delamon G, Guillemassé A, Nectoux P, et al. When Chatbots Meet Patients: One-Year Prospective Study of Conversations Between Patients With Breast Cancer and a Chatbot. JMIR Cancer. 2019;5(1):e12856. doi: 10.2196/12856 31045505 PMC6521209

[47] Echeazarra L, Pereira J, Saracho R. TensioBot: a Chatbot Assistant for Self-Managed in-House Blood Pressure Checking. J Med Syst. 2021;45(4):54. doi: 10.1007/s10916-021-01730-x 33723721

[48] Gomaa S, Posey J, Bashir B, Mallick AB, Vanderklok E, Schnoll M. Feasibility of a text messaging–integrated and chatbot-interfaced self-management program for symptom control in patients with gastrointestinal cancer undergoing chemotherapy: Pilot mixed methods study. JMIR Form Res. 2023;7:e46128. doi: 10.2196/46128 PMC10674151 37948108

[49] Gong E, Baptista S, Russell A, Scuffham P, Riddell M, Speight J, et al. My diabetes coach, a mobile app based interactive conversational agent to support type 2 diabetes self-management: randomized effectiveness-implementation trial. J Med Internet Res. 2020;22(11):e20322. doi: 10.2196/20322 33151154 PMC7677021

[50] Gómez EJ, Hernando Pérez ME, Vering T, Rigla Cros M, Bott O, García-Sáez G, et al. The INCA system: a further step towards a telemedical artificial pancreas. IEEE Trans Inf Technol Biomed. 2008;12(4):470–9. doi: 10.1109/TITB.2007.902162 18632327

[51] Hauser-Ulrich S, Künzli H, Meier-Peterhans D, Kowatsch T. A Smartphone-Based Health Care Chatbot to Promote Self-Management of Chronic Pain (SELMA): Pilot Randomized Controlled Trial. JMIR Mhealth Uhealth. 2020;8(4):e15806. doi: 10.2196/15806 32242820 PMC7165314

[52] Huang M-Y, Weng C-S, Kuo H-L, Su Y-C. Using a chatbot to reduce emergency department visits and unscheduled hospitalizations among patients with gynecologic malignancies during chemotherapy: A retrospective cohort study. Heliyon. 2023;9(5):e15798. doi: 10.1016/j.heliyon.2023.e15798 37206031 PMC10189172

[53] Krishnakumar A, Verma R, Chawla R, Sosale A, Saboo B, Joshi S, et al. Evaluating Glycemic Control in Patients of South Asian Origin With Type 2 Diabetes Using a Digital Therapeutic Platform: Analysis of Real-World Data. J Med Internet Res. 2021;23(3):e17908. doi: 10.2196/17908 33764306 PMC8074838

[54] Magnani JW, Schlusser CL, Kimani E, Rollman BL, Paasche-Orlow MK, Bickmore TW. The Atrial Fibrillation Health Literacy Information Technology System: Pilot Assessment. JMIR Cardio. 2017;1(2):e7. doi: 10.2196/cardio.8543 29473644 PMC5818980

[55] Qiu L, Kanski B, Doerksen S, Winkels R, Schmitz KH, Abdullah S. Nurse AMIE: Using smart speakers to provide supportive care intervention for women with metastatic breast cancer. In: Conference on Human Factors in Computing Systems - Proceedings. Association for Computing Machinery; 2021.

[56] Roca S, Lozano ML, García J, Alesanco Á. Validation of a Virtual Assistant for Improving Medication Adherence in Patients with Comorbid Type 2 Diabetes Mellitus and Depressive Disorder. Int J Environ Res Public Health. 2021;18(22):12056. doi: 10.3390/ijerph182212056 34831811 PMC8620667

[57] Sakane N, Suganuma A, Domichi M, Sukino S, Abe K, Fujisaki A, et al. The Effect of a mHealth App (KENPO-app) for Specific Health Guidance on Weight Changes in Adults With Obesity and Hypertension: Pilot Randomized Controlled Trial. JMIR Mhealth Uhealth. 2023;11:e43236. doi: 10.2196/43236 37043287 PMC10134028

[58] Schläpfer S, Schneider F, Santhanam P, Eicher M, Kowatsch T, Witt CM, et al. Engagement With a Relaxation and Mindfulness Mobile App Among People With Cancer: Exploratory Analysis of Use Data and Self-Reports From a Randomized Controlled Trial. JMIR Cancer. 2024;10:e52386. doi: 10.2196/52386 38819907 PMC11179041

[59] Shamekhi A, Bickmore T, Lestoquoy A, Gardiner P. Augmenting group medical visits with conversational agents for stress management behavior change. In: De Vries P, Oinas-Kukkonen H, Siemons L, Beerlage-de Jong N, van Gemert-Pijnen L, editors. Persuasive technology: Development and implementation of personalized technologies to change attitudes and behaviors. Springer International Publishing; 2017. p. 55–67.

[60] Sweidan SZ, Bouanane N, Darabkh KA. ACS: an innovative Alzheimer’s care system. Univ Access Inf Soc. 2023;23(4):1811–42. doi: 10.1007/s10209-023-01004-y

[61] Wonggom P, Nolan P, Clark RA, Barry T, Burdeniuk C, Nesbitt K, et al. Effectiveness of an avatar educational application for improving heart failure patients’ knowledge and self-care behaviors: A pragmatic randomized controlled trial. J Adv Nurs. 2020;76(9):2401–15. doi: 10.1111/jan.14414 32395836

[62] Zisis G, Carrington MJ, Oldenburg B, Whitmore K, Lay M, Huynh Q, et al. An m-Health intervention to improve education, self-management, and outcomes in patients admitted for acute decompensated heart failure: barriers to effective implementation. Eur Heart J Digit Health. 2021;2(4):649–57. doi: 10.1093/ehjdh/ztab085 36713108 PMC9707948

[63] Schlieter H, Gand K, Weimann TG, Sandner E, Kreiner K, Thoma S, et al. Designing Virtual Coaching Solutions: Design and Evaluation of a Digital Health Intervention for Rehabilitation. Bus Inform Syst Eng. 2024;66(3):377–400. doi: 10.1007/s12599-024-00871-w

[64] Voorheis P, Bhuiya AR, Kuluski K, Pham Q, Petch J. Making Sense of Theories, Models, and Frameworks in Digital Health Behavior Change Design: Qualitative Descriptive Study. J Med Internet Res. 2023;25:e45095. doi: 10.2196/45095 36920442 PMC10131681

[65] Goevaerts WF, Tenbült-van Limpt NCCW, Kop WJ, Birk MV, Liu Y, Brouwers RWM, et al. Adherence to a lifestyle monitoring system in patients with heart disease: protocol for the care-on prospective observational trial. BMC Cardiovasc Disord. 2023;23(1):196. doi: 10.1186/s12872-023-03222-x 37069506 PMC10111807

[66] Weingott S, Parkinson J. The application of artificial intelligence in health communication development: A scoping review. Health Mark Q. 2024;42(1):67–109. doi: 10.1080/07359683.2024.2422206 39556410

