# Supplementary material for: Mapping behavior change techniques and health data combinations in virtual agents for chronic condition management: A systematic scoping review
Source: PLOS Digit Health. 2026 Jul 28;5(7):e0001604. doi: 10.1371/journal.pdig.0001604 (PMC13411939; doi:10.1371/journal.pdig.0001604)
Supplement: S1 Text — (DOCX) [file pdig.0001604.s002.docx]

### Systematic Scoping Review Protocol – Chronic Disease Focus

### Title: Mapping Behavior Change Techniques and Health Data Combinations in virtual agents for Chronic Disease Management: A Systematic Scoping Review

### Background

### Introduction to Chronic Conditions & Importance of Behavior Change

### Chronic conditions, involving metabolic conditions like cardiovascular diseases and diabetes, are leading causes of morbidity and mortality worldwide. Since they require continuous health management and monitoring, they pose significant socioeconomic challenges to healthcare systems (World Health Organization, 2023). Effective management of chronic conditions often requires significant lifestyle changes, such as an improved diet, medication adherence, increased physical activity, sufficient sleep, and regular health monitoring (Debon et al., 2019). However, traditional health monitoring methods, such as periodic in-person visits to healthcare providers, can be limited in their ability to provide continuous, personalized feedback and coaching, which is crucial for sustaining long-term behavior changes. This is where digital health technologies come into play. They offer novel opportunities for real-time monitoring and personalized feedback, which can both support and enhance behavior change interventions, making chronic condition management more efficient and accessible (Viderman et al., 2022). This review aims to investigate how virtual agents, using combinations of behavior change techniques (BCTs), health data, and delivery channels, can effectively support chronic condition management.

### Digital Health-tracking devices Digital health-tracking devices such as wearables, have shown great potential in supporting the management of chronic conditions in healthcare (Mattison et al., 2022; Sun & Rau, 2015). Though according to the (Rijksinstituut voor Volksgezondheid en Milieu (RIVM)., 2023), wearables are not yet fully integrated into routine clinical care, their use is becoming more widespread, especially in self-care contexts. These health tracking devices enable the continuous collection and monitoring of (real-time) health and behavioral data, such as physical activity, heart rate, glucose levels, dietary intake, sleep, and sedentary behavior, thereby providing a unique opportunity for dynamic health management through personalized feedback reports, designed to support behavior change. A more detailed exploration of these types of health data used in digital health interventions is provided later in the review. ￼ (Griffin & Chung, n.d.) examined digital health tracking among patients with chronic conditions using data from the 2018 Health Information National Trends Survey (HINTS). They found that between 17.4 % and 37.6 % of participants reported sharing health information with health professionals through digital means, such as email and online medical records. While the study also identified some disparities in access to digital tools, particularly among patients with chronic conditions, it underscored the potential for digital technologies to enhance patient-centered care. Further, wearable devices for activity tracking have shown promise in post-surgery recovery in cardiac patients, pulmonary rehabilitation, and activity counseling in diabetic patients (Amin et al., 2021; Jo et al., 2019). These examples illustrate how digital health technologies and tracking devices can play a crucial role in improving the management and recovery process for individuals with chronic conditions.

Virtual Agents in Digital Health Interventions

Virtual agents are computer-generated characters designed to simulate human-like interactions through verbal and nonverbal cues (Philip et al., 2020). These digital entities are designed to assist users by providing information, monitoring conditions and delivering interventions. Virtual agents can take on several forms, ranging from those driven by simple rule-based algorithms to more advanced ones powered by artificial intelligence such as natural language processing and machine learning (Sheldon, 2024).

Prominent types in lifestyle-based interventions include conversational agents, embodied conversational agents, virtual coaches, and avatars. Conversational agents (CAs) can use simple rule-based algorithms or more advanced AI to mimic human interaction and have been employed in interventions for diet, exercise, stress management, and sleep(Li et al., 2023). Embodied conversational agents (ECAs) are similar but feature a visually animal or human-like virtual character. They have been effective in improving communication and engagement in interventions for elderly people with dementia by providing verbal and non-verbal cues, and in general lifestyle interventions aimed at increasing physical activity, where some studies reported measurable behavior changes, such as increased physical activity levels, though more research is needed to confirm sustained effects (Kramer et al., 2020; Rampioni et al., 2021). Virtual coaches are animated computer characters, simulating face-to-face counseling, and are used in interventions targeting health behaviors (Maher et al., 2020). Avatars, as computer-generated characters, are primarily used in telecare interventions for monitoring and interacting with patients regarding their health behaviors (Maher et al., 2020).

Overall, while all virtual agent types share some setup similarities, CAs and ECAs are more often used in health behavior interventions – structured approaches designed to modify behavior through techniques like reinforcement and self-monitoring - whereas virtual coaches, and avatars are more commonly found in broader telecare settings, which use remote technologies to deliver a range of healthcare services, including behavioral interventions, medical consultations and monitoring. Virtual agent-delivered interventions can help manage a range of health behaviors, including physical activity, dietary intake, heart rate, glucose levels, sleep and sedentary behaviour by providing personalized feedback and reminders through text messages, voice interactions, or push notifications, often using data from health-tracking devices. For instance, studies have shown that virtual agents can effectively facilitate physical activity counselling and dietary intake among people with cardiovascular diseases and promote screening and self-management of chronic diseases such as cancers, atrial fibrillation, and type 2 diabetes (Jiang et al., 2024; Lyzwinski et al., 2023). This review will focus specifically on conversational agents, embodied conversational agents, virtual coaches, and avatars, investigating how they integrate behavior change techniques and leverage health data to support lifestyle modifications.

Health Data in Digital Health Interventions

The “health data” used in digital health interventions, particularly those used or delivered by virtual agents, refers to any information regarding a person’s characteristics, health status, behaviors (such as physical activity or medication adherence), or lifestyle factors (such as diet, sleep patterns, and daily routines). This data can be used to monitor, assess and improve health outcomes to tailor the content the virtual agent provides as well as how the content is provided in an engaging manner. It can be either objective, collected through devices like wearables, or subjective, based on self-reported experiences. Table 1 below outlines the key types of health data, along with examples and important factors that influence how these data are utilized in virtual agent interventions.

**Table 1**

*Health Data Types and Their Use in Virtual Agent delivered Interventions*

| **Type of Data** | **Description** | **Examples** | **Considerations** |
| --- | --- | --- | --- |
| *Objective Health Data* | Quantifiable data collected through sensors and devices  Provides precise & continuous measurements  Used for personalized feedback & intervention adjustments | Step counts, heart rate, glucose levels, sleep patterns, blood pressure, HDL-cholesterol (Montoliu et al., 2020) | *Measurement Frequency*: Continuous or interval-based;  *Data Type:* Real-time vs. Logged data; *Approach:* Tailored Intervention adjustments based on multiple data collection points (Sieverink et al., 2017) |
| *Subjective Health Data* | Self-reported data regarding a person’s health status, symptoms, or behaviors  Provides personal insights into experiences and health behavior perceptions | Dietary intake, pain levels, mood (Viljanen et al., 2021) | *Measurement frequency:* Self-reported based on personal perceptions and experiences; provides additional behavioral insight, beyond objective measures (Viljanen et al., 2021) |
| *Contextual Data* | Data about the user’s environment or activity that can influence health outcomes and intervention personalization | User location, activity level (Craig et al., 2021; Taylor et al., 2023) | Can improve personalization and adherence to interventions (Craig et al., 2021; Taylor et al., 2023) |
| *Environmental Data* | Real-time data on external factors that may affect a person’s health | Atmospheric conditions, pollution levels (Workman et al., 2022) | Can influence patient health outcomes and provide context for tailoring interventions (Workman et al., 2022) |
| *User Preferences* | Individual choices or priorities in health care that guide the customization of interventions (Wang, 2023) | Preferred intervention methods | Helps empower patients and improve adherence to lifestyle changes (Wang, 2023) |
| *Adherence Data* | Tracking how consistently patients follow prescribed treatment regimens | Medication adherence, treatment consistency (Haberer et al., 2020) | Critical for designing effective interventions for chronic condition management (Haberer et al., 2020) |

On the one hand, *objective health data* refers to quantifiable data collected through sensors and devices, such as step counts, heart rate, glucose levels and sleeping patterns. For people with metabolic syndrome for example, common measures include blood pressure and high-density lipoprotein cholesterol (Montoliu et al., 2020). This data can be collected in real time or logged for later analysis. It provides both precise and continuous measurements, inherent for personalized feedback and intervention adjustments. On the other hand, *subjective Health data* comprises self-reported data from individuals regarding their health status, perceived symptoms, or behaviors, including dietary intake, pain levels, or mood (Viljanen et al., 2021). This type of data captures personal perceptions and experiences, providing insights into how participants feel about their health behaviors within an intervention.

Factors that also should be considered in health data, is the frequency and types of measurement, as well as the approach to dynamic tailoring. While continuous data collection allows for detailed and ongoing health metrics records, interval-based data collection, which focuses on specific times or events, can be more efficient for capturing certain events depending on the purpose of the data processing. There is also a crucial distinction between real-time data collection and logged data for later analysis purposes. Real-time data allows for immediate intervention adjustments, while logged data, depending on the timing of its review, can inform both short-term strategies (such as daily adjustments to a step goal) and provide historical insights that can inform longer-term strategies (Sieverink et al., 2017). Interventions, that then dynamically tailor their feedback based on multiple measurement moments over time can better adapt to the participants developing health status or behavior, thus improving intervention effectiveness (Araújo-Soares et al., 2019; Wang, 2023).

Beyond direct health metrics, incorporating context data, environmental data, user preferences and adherence data can provide a more comprehensive understanding of an individual's situation. Context data can include user input, activity, or location of which tracking can improve intervention personalization and adherence (Craig et al., 2021; Taylor et al., 2023). Environmental data refers to real-time data on external factors such as atmospheric conditions and pollution levels that can affect a person’s health, which can also influence patient health outcomes (Workman et al., 2022).User preferences are the individual choices or priorities of patients regarding their health care and incorporating them has been shown to empower patients and improve adherence to lifestyle modifications (Wang, 2023). Adherence data involves tracking monitoring how consistently patients follow their prescribed treatment regimens, which can be crucial for designing effective interventions, particularly for chronic conditions (Haberer et al., 2020)

Combining both objective and subjective health data can offer a comprehensive overview of a person’s health, for more effective tailoring within interventions (Jo et al., 2019). Virtual agents can leverage this integrated data to deliver more personalized and timely feedback. For this, behavior change techniques (BCTs) are commonly used.

Delivery channels of BCTs in virtual agents
How a BCT is delivered in an intervention is crucial for assessing not only the interventions effectiveness but also the effectiveness of the BCT itself. This review focuses specifically on interventions delivered through virtual agents, leveraging health data to support behavior change. These virtual agents typically operate through diverse delivery channels, which recent reviews have divided into smartphone apps, web-based platforms, desktop applications, messaging apps, tablets or even multiple of these delivery channels (Martinengo et al., 2022). Each of these delivery channels provide different means of engaging the user and delivering feedback. For instance, mobile apps can send push notifications for immediate feedback, while online portals can offer comprehensive data analysis and personalized health reports. The integration of BCTs with collected health data from digital health tracking devices such as wearables and how these combinations are delivered by the virtual agent through the mentioned delivery channels can significantly influence how effective an intervention leads to behaviour change (Eaton et al., 2024). Thus, understanding the delivery channels of virtual agents used in conjunction with BCTs and health data is essential for identifying how these combinations work to promote lifestyle change.

Need for Research on how to combine BCTs & Health Data & delivery channels within virtual agents

Despite evidence suggesting that digital interventions incorporating more BCTs tend to be more effective, the ideal number, type and combination of BCTs remain unclear (Eaton et al., 2024). Recent systematic reviews have identified specific BCTs that may be broadly applicable across diverse chronic diseases, supporting the development of disease-agnostic tools for chronic disease management (Eaton et al., 2024). Still, the optimal combinations of BCTs and health data for promoting adherence and self-management in virtual agent delivered interventions have yet to be determined. This gap exists partly because interventions such as those delivered by virtual agents utilizing BCTs are often insufficiently described in terms of how they leverage health data (Eaton et al., 2024). Next to this, it is also challenging to understand the theoretical or practice-based rationales underlying the selection and implementation of specific BCTs to encourage lifestyle change (Prestwich et al., 2014; Sediva et al., 2022). There is an increasing demand for virtual agents integrating both data-driven and theory-linked approaches, as this integration allows them to more effectively address complex and dynamic behavior changes (Singh et al., 2024).

Health interventions comprising digital elements, often also have multiple interacting components, including various BCTs and delivery modes, as mentioned above (Moller et al., 2017). Due to the adaptive and flexible nature of digital platforms such as virtual agents, participants may experience variability in their exposure to BCTs, which can complicate the assessment of intervention effectiveness (Yardley et al., 2016). (Eaton et al., 2024) also found some groups of BCTs overused, while other effective ones were underrepresented. Therefore, identifying which BCTs contribute to observed effects and understanding their mechanisms of action within complex virtual agent delivered interventions remains a significant challenge.

1.1. Objectives

This systematic scoping review aims to:

1. **Identify and catalog** the behavior change techniques (BCTs) used in combination with various types of health and behavioral data and delivery channels in interventions where a virtual agent plays a significant role in targeting lifestyle changes among people with chronic conditions.
2. **Examine the prevalence** of different BCT, health data and delivery channel combinations across different types of chronic conditions and intervention settings.
3. **Explore the theoretical and practice-based rationale** authors provide for integrating specific behavior change techniques (BCTs) and health data in virtual agent-delivered interventions aimed at promoting health behavior change.

2.0 Methodology

The systematic scoping Review will follow the steps described by (Arksey & O’Malley, 2005), with additional guidance from the Joanna Briggs Institute (JBI) for conducting systematic scoping reviews.

2.1 Identifying the Research Question

The main research question addressed within this scoping review is:

What combinations of Behavior Change Techniques (BCTs), health data and delivery channels are currently used in interventions aimed at behavior and lifestyle changes where virtual agents are used in managing chronic conditions and how do these combinations operate and vary across different contexts in terms of their prevalence and theoretical and practical underpinnings?

To break it down, the following sub-questions are addressed:

1.0 (**Identification & Cataloguing)**: What specific Behavior Change Techniques (BCTs) are used in conjunction with which types of health data and with which delivery channels in interventions involving virtual agents for people with chronic conditions?

1.1 (**Prevalence & Context of combinations**): How prevalent are different combinations of BCTs, health data types and delivery channels in virtual agent-delivered interventions for chronic conditions and in what contexts are these combinations applied (e.g., study population, intervention aim, setting, type of health data, BCTs used, virtual agent delivery mode and rationale behind design choices)?

1.2 (**Theoretical and practical underpinnings**): According to the literature, what rationale - whether theoretical or practice-based - do authors provide for the reported combinations of behavior change techniques (BCTs) and health data types in virtual agent interventions for managing chronic conditions?

2.2. Identifying Relevant studies

- **Databases**: PubMed, Scopus, PsycINFO, Web of Science, IEEE Xplore

1. **Scopus**: Chosen due to being a comprehensive, multi-disciplinary abstract & citation database, with a broad coverage of medical informatics research (relevant for me due to its health and technology focus)
2. **PubMed**: Specializes in biomedical and life sciences literature, making it a valuable resource for evidence-based studies/clinical applications relevant to health and behavior interventions
3. **PsycINFO**: Provides extensive coverage of psychology and behavioral sciences, which is ideal for (my) research focusing on behavior change techniques in chronic disease management
4. **IEEE Explore**: Strong database in the field of engineering and technology, including digital health interventions and wearable technology, thus offering technical insights essential for informatics and virtual agent design
5. **Web of Science**: Includes high-impact journals across various disciplines, and hence useful for me to track foundational and cross-disciplinary studies in medical informatics, health data, and digital interventions

- **Search Terms:**

**Search string (adapted per database)**

| ("Conversational agent*" OR "Conversational system*" OR "Virtual agent*" OR "Relational agent*" OR "Digital agent*" OR "Digital assistant*" OR "Chatbot*" OR "Dialogue system*" OR "Dialog system*" OR "Assistance technology" OR "Virtual assistant*" OR "AI agent*" OR "Embodied agent*" OR "Embodied conversational agent" OR "Social agent*" OR "Virtual coach" OR "Virtual counselor*" OR "Virtual counsellor*" OR "Virtual health counselor*" OR "Virtual health counsellor*" OR "Virtual health agent*" OR "Virtual health coach" OR "Virtual human" OR "Virtual patient advocate*" OR "Avatar*")  AND ("Healthcare" OR "Health care" OR "Digital health*" OR "mHealth" OR "m-Health" OR "eHealth" OR "e-health" OR "Tele-medicine" OR "Telemedicine" OR "Tele-health" OR "Telehealth" OR "Health technology" OR "Self-management" OR "Self management" OR "Behavior change intervention" OR "Behaviour change intervention" OR "Digital intervention" OR "Internet intervention" OR "Health informatics")  AND ("Chronic disease*" OR "Chronic condition*" OR "Chronic illness*" OR "Noncommunicable disease*" OR "Non-communicable disease*" OR "Diabetes" OR "Heart disease" OR "Hypertension" OR "Metabolic Syndrome" OR "COPD" OR "Cancer" OR "Parkinson's disease" OR "Alzheimer's disease" OR "Obesity" OR "Chronic Pain") |
| --- |

- **Date range**: None
- **Language:** English
- **Source Type:** Peer-reviewed/ accepted journal articles, conference papers, case study, observational study
- References extracted from the mentioned databases will be combined & imported into Covidence (Duplicates will be automatically removed)
- Selection of eligible studies: screening process with second reviewer (title, abstract and full-text screening)

2.3. Selection of eligible studies

**Screening Process:** Titles & Abstracts will be screened independently by 2 reviewers in Covidence to identify relevant articles against specific inclusion criteria, followed by full-text screening to confirm the inclusion 
 
**Disagreement Resolution:** Discrepancies will be resolved through discussion (e.g. 2^nd^ Reviewer will screen 20 % of the abstracts and after 10 % we will shortly discuss, based on the inter-rater agreement)

2.3.1 Inclusion criteria (Full text)

- **Study Design and Source Type:**
  - This review includes studies that test the intervention with the target group of adults with a diagnosed chronic condition. Accepted designs include:
    - Randomized Controlled Trials (RCTs)
    - Quasi-experimental studies
    - Longitudinal studies
    - Observational studies
    - Qualitative studies
    - Etc.
  - Peer-reviewed journal articles or conference papers only; no non-peer reviewed materials/ articles
- **Intervention Type**:
  - The study must involve a peer-reviewed virtual agent-delivered intervention targeting lifestyle/ behavior changes in the context of chronic condition care/ management.
    - Studies where the virtual agent is a central, but not sole component of the intervention are also included
    - Virtual agents included in the study must exhibit conversational interactivity, defined as working with user inputs and providing outputs based on those inputs in an interactive manner (even if they are simple rule-based agents)
    - The intervention must mention the use of health data (subjective or objective) to inform the intervention
    - The study should incorporate the use of Behavior Change Techniques (BCTs) or at least reference to an underlying behavioral theory
- **Population:**
  - The study must involve adults (18 years or older) with a diagnosed chronic condition (e.g., diabetes, cardiovascular disease, COPD, cancer, obesity, hypertension, long covid etc.)
- **Health Data Use:**
  - The intervention must use health data (subjective or objective) as input, such as tracked data from wearables, self-reported symptoms or behavioral tracking (e.g., diet, physical activity, medication adherence)
- **Outcomes**:
  - The study should report on one or more of the following outcomes:
    - **Behavior change outcomes** (e.g., increased physical activity, improved diet, medication adherence)
    - **Health outcomes** (e.g., improved clinical markers like weight loss, blood pressure etc.)
    - **Usability/ Acceptability/ satisfaction** with the intervention (e.g., participant feedback, qualitative assessment)
- **Context**:
  - The intervention should take place in clinical, community, home-based, or other real-world settings

2.4. Charting the data

To support the above-mentioned objectives of this review, Table 2 will catalog specific study details per included paper, and based on this, table 3 will provide an overview of common BCT and health data combinations, highlighting their application contexts and examples of use from table 1.

Understanding how BCTs and health data are integrated is crucial for optimizing virtual agent-delivered interventions in chronic care. Virtual agents provide continuous, personalized interactions that adapt to the user's health status and behaviors, making them particularly valuable in chronic disease management. This review will provide valuable insights into the design of more effective, evidence-based digital health interventions, informing future research on the most effective BCTs and health data combinations for chronic disease management, next to future research directions based on identified gaps.

**Table 2**

*Specifics of Each Study, Aligning With Objective 1*

| Study  (Title/Author/Year) | Intervention Aims | Context (Setting/ Target Population) | Type of Data (Also: used as Input/utput or both) | BCTs Used | Mode of delivery in agent | Theoretical/ Practical design rationale |
| --- | --- | --- | --- | --- | --- | --- |
| Example (study 1, authors) | Improve PA behaviour in cardiovascular patients | Home-based, adults with cardiovascular disease | (Physical activity) Input: Step counts, heart rate  Output: Personalized feedback | Goal-setting, self-monitoring | Mobile App with virtual coach; JITAI, offered via spoken text messages | Grounded in Self-determination theory (SDT); tailored to user stages of change |

**Table 2**

*Summary of Common Combinations and Contexts (Objectives 2/3/4)*

| BCTs | Type of Health Data | Common Contexts | Examples of Use |
| --- | --- | --- | --- |
| Example: Goal setting | Step counts, dietary intake | Physical activity, dietary changes | Study 1, Study 2 |

2.5. Collating, summarizing & reporting results

- **Descriptive Summary** of study characteristics, including types of BCTs real-time data and delivery channels used
  - Based on (Arksey & O’Malley, 2005), highlighting the importance of providing a descriptive summary of study characteristics, including the types of studies included, their aims and key findings
- **Thematic Analysis** to identify common themes regarding the theoretical & practice-based rationales of the BCT, real-time data and delivery channel combinations
  - Based on the framework of (Braun & Clarke, 2006), comprising the identification, analysis and reporting of patterns (themes) within data
  - Helps in understanding the common mechanisms and underpinnings of BCTs, real-time data and delivery channel combinations
- **Frequency Analysis** to analyse prevalence of specific combinations & contexts in which those are applied
- **Contextual analysis** to assess variations in combinations and outcomes across different contexts (types of illnesses/ intervention settings etc.)

3.0 Discussion

I will provide a summary of findings of the common combinations of BCTs, real-time health data and delivery channel combinations and their underlying mechanisms. Next, I will discuss implications for practice, future research directions into identifying the most appropriate BCTs and acknowledge any strengths & limitations of this scoping review.

**References**

Amin, T., Mobbs, R. J., Mostafa, N., Sy, L. W., & Choy, W. J. (2021). Wearable devices for patient monitoring in the early postoperative period: A literature review. In *mHealth* (Vol. 7). AME Publishing Company. https://doi.org/10.21037/mhealth-20-131

Araújo-Soares, V., Hankonen, N., Presseau, J., Rodrigues, A., & Sniehotta, F. F. (2019). Developing Behavior Change Interventions for Self-Management in Chronic Illness: An Integrative Overview. In *European Psychologist* (Vol. 24, Issue 1, pp. 7–25). Hogrefe Publishing GmbH. https://doi.org/10.1027/1016-9040/a000330

Arksey, H., & O’Malley, L. (2005). Scoping studies: Towards a methodological framework. *International Journal of Social Research Methodology: Theory and Practice*, *8*(1), 19–32. https://doi.org/10.1080/1364557032000119616

Braun, V., & Clarke, V. (2006). Using thematic analysis in psychology. *Qualitative Research in Psychology*, *3*(2), 77–101. https://doi.org/10.1191/1478088706qp063oa

Craig, K. J. T., Morgan, L. C., Chen, C. H., Michie, S., Fusco, N., Snowdon, J. L., Scheufele, E., Gagliardi, T., & Sill, S. (2021). Systematic review of context-aware digital behavior change interventions to improve health. In *Translational Behavioral Medicine* (Vol. 11, Issue 5, pp. 1037–1048). Oxford University Press. https://doi.org/10.1093/tbm/ibaa099

Debon, R., Coleone, J. D., Bellei, E. A., & De Marchi, A. C. B. (2019). Mobile health applications for chronic diseases: A systematic review of features for lifestyle improvement. In *Diabetes and Metabolic Syndrome: Clinical Research and Reviews* (Vol. 13, Issue 4, pp. 2507–2512). Elsevier Ltd. https://doi.org/10.1016/j.dsx.2019.07.016

Duff, O. M., Walsh, D. M. J., Furlong, B. A., O’Connor, N. E., Moran, K. A., & Woods, C. B. (2017). Behavior change techniques in physical activity eHealth interventions for people with cardiovascular disease: Systematic review. In *Journal of Medical Internet Research* (Vol. 19, Issue 8). JMIR Publications Inc. https://doi.org/10.2196/jmir.7782

Eaton, C. K., McWilliams, E., Yablon, D., Kesim, I., Ge, R., Mirus, K., Sconiers, T., Donkoh, A., Lawrence, M., George, C., Morrison, M. L., Muther, E., Oates, G. R., Sathe, M., Sawicki, G. S., Snell, C., & Riekert, K. (2024). Cross-Cutting mHealth Behavior Change Techniques to Support Treatment Adherence and Self-Management of Complex Medical Conditions: Systematic Review. In *JMIR mHealth and uHealth* (Vol. 12). JMIR Publications Inc. https://doi.org/10.2196/49024

Griffin, A. C., & Chung, A. E. (n.d.). *Health Tracking and Information Sharing in the Patient-Centered Era: A Health Information National Trends Survey (HINTS) Study*.

Haberer, J. E., Garrison, L., Tumuhairwe, J. B., Baijuka, R., Tindimwebwa, E., Tinkamanyire, J., Burns, B. F., & Asiimwe, S. (2020). Factors affecting the implementation of electronic antiretroviral therapy adherence monitoring and associated interventions for routine HIV care in Uganda: Qualitative study. *Journal of Medical Internet Research*, *22*(9). https://doi.org/10.2196/18038

Jiang, Z., Huang, X., Wang, Z., Liu, Y., Huang, L., & Luo, X. (2024). Embodied Conversational Agents for Chronic Diseases: Scoping Review. In *Journal of Medical Internet Research* (Vol. 26, Issue 1). JMIR Publications Inc. https://doi.org/10.2196/47134

Jo, A., Coronel, B. D., Coakes, C. E., & Mainous, A. G. (2019). Is There a Benefit to Patients Using Wearable Devices Such as Fitbit or Health Apps on Mobiles? A Systematic Review. In *American Journal of Medicine* (Vol. 132, Issue 12, pp. 1394-1400.e1). Elsevier Inc. https://doi.org/10.1016/j.amjmed.2019.06.018

Kramer, L. L., Ter Stal, S., Mulder, B. C., De Vet, E., & Van Velsen, L. (2020). Developing embodied conversational agents for coaching people in a healthy lifestyle: Scoping review. In *Journal of Medical Internet Research* (Vol. 22, Issue 2). JMIR Publications Inc. https://doi.org/10.2196/14058

Li, Y., Liang, S., Zhu, B., Liu, X., Li, J., Chen, D., Qin, J., & Bressington, D. (2023). Feasibility and effectiveness of artificial intelligence-driven conversational agents in healthcare interventions: A systematic review of randomized controlled trials. In *International Journal of Nursing Studies* (Vol. 143). Elsevier Ltd. https://doi.org/10.1016/j.ijnurstu.2023.104494

Lyzwinski, L. N., Elgendi, M., & Menon, C. (2023). Conversational Agents and Avatars for Cardiometabolic Risk Factors and Lifestyle-Related Behaviors: Scoping Review. In *JMIR mHealth and uHealth* (Vol. 11). JMIR Publications Inc. https://doi.org/10.2196/39649

Maher, C. A., Davis, C. R., Curtis, R. G., Short, C. E., & Murphy, K. J. (2020). A physical activity and diet program delivered by artificially intelligent virtual health coach: Proof-of-concept study. *JMIR MHealth and UHealth*, *8*(7). https://doi.org/10.2196/17558

Martinengo, L., Jabir, A. I., Goh, W. W. T., Lo, N. Y. W., Ringo Ho, M. H., Kowatsch, T., Atun, R., Michie, S., & Car, L. T. (2022). Conversational Agents in Health Care: Scoping Review of Their Behavior Change Techniques and Underpinning Theory. In *Journal of Medical Internet Research* (Vol. 24, Issue 10). JMIR Publications Inc. https://doi.org/10.2196/39243

Mattison, G., Canfell, O., Forrester, D., Dobbins, C., Smith, D., Töyräs, J., & Sullivan, C. (2022). The Influence of Wearables on Health Care Outcomes in Chronic Disease: Systematic Review. In *Journal of Medical Internet Research* (Vol. 24, Issue 7). JMIR Publications Inc. https://doi.org/10.2196/36690

Michie, S., Richardson, M., Johnston, M., Abraham, C., Francis, J., Hardeman, W., Eccles, M. P., Cane, J., & Wood, C. E. (2013). The Behavior Change Technique Taxonomy (v1) of 93 Hierarchically Clustered Techniques: Building an International Consensus for the Reporting of Behavior Change Interventions. *Annals of Behavioral Medicine*, *46*(1), 81–95. https://doi.org/10.1007/s12160-013-9486-6

Michie, S., van Stralen, M. M., & West, R. (2011). The behaviour change wheel: A new method for characterising and designing behaviour change interventions. *Implementation Science*, *6*(1). https://doi.org/10.1186/1748-5908-6-42

Moller, A. C., Merchant, G., Conroy, D. E., West, R., Hekler, E., Kugler, K. C., & Michie, S. (2017). Applying and advancing behavior change theories and techniques in the context of a digital health revolution: proposals for more effectively realizing untapped potential. *Journal of Behavioral Medicine*, *40*(1), 85–98. https://doi.org/10.1007/s10865-016-9818-7

Montoliu, T., Hidalgo, V., & Salvador, A. (2020). Importance of personality for objective and subjective-physical health in older men and women. *International Journal of Environmental Research and Public Health*, *17*(23), 1–13. https://doi.org/10.3390/ijerph17238809

Munson, S. A., & Consolvo, S. (2012). Exploring goal-setting, rewards, self-monitoring, and sharing to motivate physical activity. *2012 6th International Conference on Pervasive Computing Technologies for Healthcare and Workshops, PervasiveHealth 2012*, 25–32. https://doi.org/10.4108/icst.pervasivehealth.2012.248691

Philip, P., Dupuy, L., Morin, C. M., de Sevin, E., Bioulac, S., Taillard, J., Serre, F., Auriacombe, M., & Micoulaud-Franchi, J. A. (2020). Smartphone-based virtual agents to help individuals with sleep concerns during COVID-19 confinement: Feasibility study. *Journal of Medical Internet Research*, *22*(12). https://doi.org/10.2196/24268

Prestwich, A., Sniehotta, F. F., Whittington, C., Dombrowski, S. U., Rogers, L., & Michie, S. (2014). Does theory influence the effectiveness of health behavior interventions? Meta-analysis. *Health Psychology*, *33*(5), 465–474. https://doi.org/10.1037/a0032853

Rampioni, M., Stara, V., Felici, E., Rossi, L., & Paolini, S. (2021). Embodied conversational agents for patients with dementia: Thematic literature analysis. In *JMIR mHealth and uHealth* (Vol. 9, Issue 7). JMIR Publications Inc. https://doi.org/10.2196/25381

Rijksinstituut voor Volksgezondheid en Milieu (RIVM). (2023). *2023 E-health Monitor 2023.  State of digital care.* From E-Healthmonitor 2023. Stand van Zaken Digitale  Zorg | RIVM.

Sediva, H., Cartwright, T., Robertson, C., & Deb, S. K. (2022). Behavior Change Techniques in Digital Health Interventions for Midlife Women: Systematic Review. In *JMIR mHealth and uHealth* (Vol. 10, Issue 11). JMIR Publications Inc. https://doi.org/10.2196/37234

Sheldon, R. , H.-P. C. , & B. B. (2024, June 1). *What is a virtual agent? TechTarget.* Https://Www.Techtarget.Com/Searchcustomerexperience/Definition/Virtual-Agent.

Sieverink, F., Kelders, S., Poel, M., & van Gemert-Pijnen, L. (2017). Opening the black box of electronic health: Collecting, analyzing, and interpreting log data. *JMIR Research Protocols*, *6*(8). https://doi.org/10.2196/resprot.6452

Singh, T., Truong, M., Roberts, K., & Myneni, S. (2024). Sequencing conversational turns in peer interactions: An integrated approach for evidence-based conversational agent for just-in-time nicotine cravings intervention. *Digital Health*, *10*. https://doi.org/10.1177/20552076241228430

Sun, N., & Rau, P. L. P. (2015). The acceptance of personal health devices among patients with chronic conditions. *International Journal of Medical Informatics*, *84*(4), 288–297. https://doi.org/10.1016/j.ijmedinf.2015.01.002

Taylor, K. S., Umeukeje, E. M., Santos, S. R., Mcnabb, K. C., Crews, D. C., & Hladek, M. D. (2023). Context Matters: A Qualitative Synthesis of Adherence Literature for People on Hemodialysis. *Kidney360*, *4*(1), 41–53. https://doi.org/10.34067/KID.0005582022

Viderman, D., Seri, E., Aubakirova, M., Abdildin, Y., Badenes, R., & Bilotta, F. (2022). Remote Monitoring of Chronic Critically Ill Patients after Hospital Discharge: A Systematic Review. In *Journal of Clinical Medicine* (Vol. 11, Issue 4). MDPI. https://doi.org/10.3390/jcm11041010

Viljanen, A., Salminen, M., Irjala, K., Heikkilä, E., Isoaho, R., Kivelä, S. L., Korhonen, P., Vahlberg, T., Viitanen, M., Wuorela, M., Löppönen, M., & Viikari, L. (2021). Subjective and objective health predicting mortality and institutionalization: an 18-year population-based follow-up study among community-dwelling Finnish older adults. *BMC Geriatrics*, *21*(1). https://doi.org/10.1186/s12877-021-02311-w

Wang, W. (2023). Towards Adaptive User Interfaces: A Model-Driven Approach for mHealth Applications Targeting Chronic Disease. *Proceedings of IEEE Symposium on Visual Languages and Human-Centric Computing, VL/HCC*, 296–298. https://doi.org/10.1109/VL-HCC57772.2023.00058

Workman, A., Johnston, F. H., Campbell, S. L., Williamson, G. J., Lucani, C., Bowman, D. M. J. S., Cooling, N., & Jones, P. J. (2022). Evaluating User Preferences, Comprehension, and Trust in Apps for Environmental Health Hazards: Qualitative Case Study. *JMIR Formative Research*, *6*(12). https://doi.org/10.2196/38471

World Health Organization. (2023, September 16). *Noncommunicable diseases. World Health Organization.* Https://Www.Who.Int/News-Room/Fact-    Sheets/Detail/Noncommunicable-Diseases.

Yardley, L., Spring, B. J., Riper, H., Morrison, L. G., Crane, D. H., Curtis, K., Merchant, G. C., Naughton, F., & Blandford, A. (2016). Understanding and Promoting Effective Engagement With Digital Behavior Change Interventions. *American Journal of Preventive Medicine*, *51*(5), 833–842. https://doi.org/10.1016/j.amepre.2016.06.015
