# Supplementary material for: Mapping behavior change techniques and health data combinations in virtual agents for chronic condition management: A systematic scoping review
Source: PLOS Digit Health. 2026 Jul 28;5(7):e0001604. doi: 10.1371/journal.pdig.0001604 (PMC13411939; doi:10.1371/journal.pdig.0001604)
Supplement: S2 Text — (DOCX) [file pdig.0001604.s003.docx]

# **Key Concepts and most relevant terms**

- Date of Search: 10-09-2024
- Language: English
- Keywords : See Table below, according to PCC structure (Population, Context, Concept)

| **Virtual agent** | **(digital) health** | **Intervention** | **Chronic condition** |
| --- | --- | --- | --- |
| Conversational agent, Conversational system, Virtual agent, Relational agent, Digital agent, Digital assistant, Chatbot, Dialogue system, Dialog system, Assistance technology, Virtual assistant, AI agent, Embodied agent, Embodied conversational agent, Social agent, Virtual coach, Virtual counselor, Virtual counsellor, Virtual health counselor, Virtual health counsellor, Virtual health agent, Virtual health coach, Virtual human, Virtual patient advocate, Avatar | Healthcare, Health care, Digital health, mHealth, m-Health, eHealth, e-health, Tele-medicine, Telemedicine, Tele-health, Telehealth, Health technology, Health informatics | Self-management, Self management, Behavior change intervention, Behaviour change intervention, Digital intervention, Internet intervention, | Chronic disease, Chronic condition, Chronic illness, Noncommunicable disease, Non-communicable disease, Diabetes, Heart disease, Hypertension, Metabolic Syndrome, COPD, Cancer, Parkinson's disease, Alzheimer's disease, Obesity, Chronic Pain |

# **Full Search Strategy**

| TITLE-ABS-KEY("Conversational agent*" OR "Conversational system*" OR "Virtual agent*" OR "Relational agent*" OR "Digital agent*" OR "Digital assistant*" OR "Chatbot*" OR "Dialogue system*" OR "Dialog system*" OR "Assistance technology" OR "Virtual assistant*" OR "AI agent*" OR "Embodied agent*" OR "Embodied conversational agent" OR "Social agent*" OR "Virtual coach" OR "Virtual counselor*" OR "Virtual counsellor*" OR "Virtual health counselor*" OR "Virtual health counsellor*" OR "Virtual health agent*" OR "Virtual health coach" OR "Virtual human" OR "Virtual patient advocate*" OR "Avatar*")    AND    TITLE-ABS-KEY("Healthcare" OR "Health care" OR "Digital health*" OR "mHealth" OR "m-Health" OR "eHealth" OR "e-health" OR "Tele-medicine" OR "Telemedicine" OR "Tele-health" OR "Telehealth" OR "Health technology" OR "Self-management" OR "Self management" OR "Behavior change intervention" OR "Behaviour change intervention" OR "Digital intervention" OR "Internet intervention" OR "Health informatics")    AND    TITLE-ABS-KEY("Chronic disease*" OR "Chronic condition*" OR "Chronic illness*" OR "Noncommunicable disease*" OR "Non-communicable disease*" OR "Diabetes" OR "Heart disease" OR "Hypertension" OR "Metabolic Syndrome" OR "COPD" OR "Cancer" OR "Parkinson's disease" OR "Alzheimer's disease" OR "Obesity" OR "Chronic Pain") |
| --- |
