# Supplementary material for: Mapping behavior change techniques and health data combinations in virtual agents for chronic condition management: A systematic scoping review
Source: PLOS Digit Health. 2026 Jul 28;5(7):e0001604. doi: 10.1371/journal.pdig.0001604 (PMC13411939; doi:10.1371/journal.pdig.0001604)
Supplement: S1 Table — (DOCX) [file pdig.0001604.s004.docx]

**S1 Data Extraction Template**

| **Category** | **Variable** | **Options (if applicable)** |
| --- | --- | --- |
| **Study Information** | Study ID/DOI |  |
|  | TItle |  |
|  | Authors |  |
|  | Authors contact |  |
|  | Publication Year |  |
|  | Journal Name |  |
|  | Study Country |  |
|  | Notes |  |
| **Study Characteristics** | Study Aim |  |
| - *Study Design* | Study Type | RCT, Quasi-experimental, Longitudinal, Observational, Case study, Mixed-methods, Other |
|  | Control Group | Yes, No, Not applicable |
|  | Blinding | Yes, No, Not reported |
| **Study Population** | Initial Sample Size |  |
|  | Actual Sample Size |  |
|  | Mean Age |  |
|  | Inclusion Criteria |  |
|  | Exclusion Criteria |  |
|  | Recruitment Method | Phone, Mail, Clinic patients, Voluntary, Other |
| **Intervention** | Intervention Duration |  |
|  | Intervention Frequency |  |
|  | Intervention Name |  |
|  | Project Name |  |
|  | Intervention Focus | Behavior change, Health Data collection, Medication Adherence, Lifestyle Modification, Self-management, Behavior Change & Mental Health, Other |
| - *Virtual Agent* | Virtual Agent Type | CA, ECA, Virtual Coach, Avatar, Other |
|  | System Type | Simple rule-based, Rule-based & limited AI adaption, Generative AI (LLMs), Hybrid (Rule-based & generative AI), Other |
|  | Virtual Agent Notes |  |
|  | Virtual Agent Role | Sole medium of intervention, Part of blended care, Other |
| - *BCTs* | BCT Type | Self-monitoring of behavior; Goal-setting (behavioral); Feedback on behavior; Instruction on how to perform a behavior; Education or information provision; Social support; Motivation/reinforcement; Prompts/cues (e.g., reminders); Self-regulation of behavior; Other |
|  | BCT reported or inferred | Explicitly reported, Inferred from text, Other |
| - *Health Data* | Health Data Input | Objective, Subjective, Contextual, Environmental, Adherence, Other |
|  | Input into System | Reported by participant, Reported by device, Both, Other |
|  | Health Data Output | Feedback/status, Visual data, Actionable recommendations, Other |
|  | Data Collection Tools | Wearables, Mobile apps, Surveys, Other |
| - *Delivery Channels* | Delivery Channel Type | Apps, Web platforms, SMS, Email, Mobile devices, Voice assistants, Other |
|  | Real-world Setting | Home, Clinical setting, Hospital discharge, Community-based, Other |
| - *Design Rationales* | Theoretical Rationales |  |
|  | Practical Rationales |  |
| **Study Outcomes** | Primary Outcome Types | Behavior change, Health outcomes, Medication adherence, Other |
|  | Outcome Measurements | Self-reports, Physiological, Clinician assessments, Other |
|  | Reported Outcomes |  |
| **Analysis** | Statistical Analysis | Descriptive, Inferential, Regression, Other |
| **Study Evaluation** | Evaluation Outcome Types | Satisfaction, Ease of use, Adherence rates, Engagement, Technical issues, Other |
|  | Evaluation Measurement | Surveys, Interviews/Focus groups, Retention/drop-out rates, Other |
|  | Evaluation Reported |  |
|  | Future Research Directions |  |
