# Supplementary material for: Mapping behavior change techniques and health data combinations in virtual agents for chronic condition management: A systematic scoping review
Source: PLOS Digit Health. 2026 Jul 28;5(7):e0001604. doi: 10.1371/journal.pdig.0001604 (PMC13411939; doi:10.1371/journal.pdig.0001604)
Supplement: S2 Table — (DOCX) [file pdig.0001604.s005.docx]

**S2 BCT coding evidence**

| **Study** | **BCT** | **Explicitly reported or Inferred from text** | **Evidence from text** |
| --- | --- | --- | --- |
| Albino de Quiroz et al., 2023 | **Self-monitoring of Behavior; Feedback on Behavior; Prompts/cues** | Inferred from text | Self-monitoring: "Patients self-reported perceived symptoms and adverse effects, practiced physical activity, and data about their diet"  Feedback on behavior: Patients received feedback on reported clinical condition or activity ("automated and individualized feedback according to the interaction").  Prompts/cues: "Participants received alerts encouraging the use and reminding them of the importance of using the SMT." |
| Baptista et al., 2020 | **Self-monitoring of Behavior; Goal-setting (behavioral); Feedback on Behavior; Instruction on how to perform a behavior; Social reward; Prompts/cues (e.g., reminders)** | Inferred from text | Self-monitoring: "Laura provided(…) support for blood glucose level monitoring, taking medication, physical activity, healthy eating, and foot care."  Feedback on behavior: "Laura provided (…) feedback for blood glucose level monitoring, taking medication, physical activity, healthy eating, and foot care.”  Instruction on how to perform a behavior: "Laura provided (…) education for blood glucose level monitoring, taking medication, physical activity, healthy eating, and foot care."  Social reward: "Laura provided (…) motivational support for blood glucose level monitoring, taking medication, physical activity, healthy eating, and foot care."  Prompts/cues: "(…) the were prompted to set up a regular time to complete weekly interactive sessions with Laura.”  Goal-setting: "you can set goals for yourself that I'll support you reaching" from YT video of Laura interaction |
| Chaix et al., 2019 | **Self-monitoring of Behavior; Instruction on how to perform a behavior; Prompts/cues** | Inferred from text | Self-monitoring of behavior: "The chatbot...send[s] reminders...with 3 possible choices: 'yes I took it,' 'no I didn’t take it,' or 'send me the message in 15 minutes.'"  Instruction on how to perform a behavior: "Vik informs about breast cancer and its epidemiology, treatments and their side effects, and the quality of life, with information about sport, fertility, sexuality, and diet.", "educational content explaining to patients how to take their medication properly, why they have this side effect and how they can avoid it".  Prompts/cues: "We implemented a medication reminder function...The chatbot will then send this person a reminder to take the medication with 3 possible answer choices." |
| Echeazarra et al., 2021 | **Self-monitoring of Behavior; Feedback on Behavior; Instruction on how to perform a behavior; Prompts/cues** | Inferred from Text | Self-monitoring & Feedback on behavior: "After the second set of measurements, TensioBot shows a line chart with the entire BP history."  Instruction on how to perform a behavior: "Once a day the bot also sends a message with tips related to good BP measurement practices and offers an option to display a helpful video on good BP measurement practices showing tips on how and  when to take it, how to adjust the tensiometer, how to adjust the body position."  Prompts/cues: " TensioBot asks  patients to measure their BP twice a day, usually once in  the morning and once in the evening (the alert times can be  edited at any time). When the user receives the alert, he/she  should proceed to use the tensiometer."; “the Bot offers a command to remind the patient of the scheduled time for the next medical appointment,” |
| Gomaa et al., 2023 | **Self-monitoring of Behavior; Feedback on Behavior; Instruction on how to perform a behavior**; Prompts/cues | Inferred from text | Self-monitoring: “A chatbot interface was seamlessly integrated into the intervention, using text messages to regularly monitor  participants’ symptoms. This feature allowed participants to  assess their symptoms at their convenience, providing them  with a user-friendly and accessible means of reporting their  experiences..”  Feedback on Behavior: "…integral aspect of the intervention was the provision of immediate feedback based on the symptom assessments collected via the chatbot. This feedback offered participants valuable guidance on self-care actions to be taken in response to their reported symptoms."  Prompts/Cues: " Participants received proactive and interactive text messages 3 times a week."  Instruction on how to perform a behavior: "…messages 3 times a week including chemotherapy-related knowledge, symptom management, self-care and lifestyle behavioral changes." |
| Gong et al,, 2020 | **Self-monitoring of** **Behavior; Feedback on Behavior; Instruction on how to perform a behavior;** Social support**; Problem-solving; Information on health consequences** | Inferred from text (though they did mention they “used BCTs”) | Self-Monitoring of Behavior: “MDC delivers monitoring (…) via an embodied conversational agent covering blood glucose monitoring, healthy eating, physical activity (…).”  Feedback on Behavior: “Each appointment module with Laura began with a review of progress with feedback…”.  Instruction on How to Perform a Behavior/ Information on health consequences: “Users received guidance on managing diabetes through education and counseling modules (e.g., healthy eating, MA).”  Social Support: “Online discussion board where program coordinator assisted participants (…).”  Problem-solving: "Modules incorporated tips on overcoming barriers". |
| Gomez et al., 2008 | **Self-monitoring of Behavior; Feedback on Behavior** | Inferred from text | Self-monitoring of behavior: continuous glucose monitoring, insulin logs.  Feedback on behavior: real-time glucose data and insulin trends visible, therapy recommendations of HPC via TMCS messages, or/ and direct integration into PDA. |
| Hauser-Ulrich et al.,, 2020 | **Self-monitoring of Behavior; Feedback on Behavior; Instruction on how to perform a behavior; Education or information provision; Prompts/cues; Reduce negative emotions** | Inferred from text | Self-monitoring of behavior: encouraged self-reflection on pain and coping behaviors, reinforcing self-management strategies. (Figure 1. Overview of Intervention Schedule)  Feedback on Behavior: " To support establishment of a working  alliance, user engagement, and motivation, SELMA addresses  participants’ accountability by referring to earlier tasks and  activities (eg, “Welcome back to the coaching! Were you able  to relax yesterday?”); she supports the completion of activities  and tasks (eg, “Hi [$nickname]. How is it going with practicing  your exercises?”), and motivates participants to repeat them  (eg, “How did you manage the exercise, perhaps you can repeat  it before the next time we meet?”)”  Instruction on how to perform a behavior : "The intervention was structured in modules, including psychoeducation, mindfulness, relaxation exercises, and cognitive reframing techniques.", "Participants were instructed to apply pain diary.”; “module mindfulness, a coping strategy module, briefly  explains the concept of mindfulness and provides users with a  mindfulness exercise. Specifically, SELMA instructs participants  on how to integrate mindfulness into their daily routine and  provides users with a relaxation exercise”  Prompts/cues (e.g., reminders): " . These notifications are sticky, meaning that they are displayed in the notification dashboard and thus act as reminders. If the app is already opened, then no additional notification is triggered..”  Reduce negative emotions: " For example, the module about  dysfunctional behavior covers avoidance of activity. It explains  the link between avoiding activity and pain, and SELMA  motivates participants to start physical activity and reminds  them to keep their own level of proficiency in mind.”; “6 modules that either address dysfunctional  cognitions, behavior, and emotions (eg, stress, fear of pain,  anxiety) or various coping strategies (eg, activity, resources,  mindfulness, acceptance).” |
| Huang et al., 2023 | **Self-monitoring of Behavior; Feedback on Behavior; Instruction on how to perform a behavior; Prompts/cues** | Inferred from text | Self-monitoring of Behavior; Feedback on Behavior, Instruction on how to perform a behavior & Prompts/cues (e.g., reminders): "Chatbot sends notifications to patients regarding the patients’ conditions. The patients then respond to the chatbot based on the symptoms they have, and the chatbot will send prearranged suggestions about how to relieve these symptoms directly to the patients.”; “natural language generation to user responses based on pre-defined templates of health instructions.” |
| Krishnakumar et al., 2021 | **Self-monitoring of Behavior; Feedback on Behavior; Instruction on how to perform a behavior; Education or information provision;** Social support**; Prompts/cues; Problem-solving, Rewards (outcome)** | Inferred from text | Self-monitoring of Behavior; Feedback on Behavior; Instruction on how to perform a behavior; Education or information provision; Social support; Prompts/cues (e.g., reminders); Problem-solving & Rewards (outcome):  "System coached participants along 7 tracks that covered eating healthy, becoming more active, improving self-monitoring, improving medication adherence, problem solving, reducing risk, and healthy coping"; "Feedback delivered in real time through a conversation experience by an AI-powered chatbot that provided educational, behavioral, and motivational messaging specific to the data entered and in the context of the patient’s previous clinical, lifestyle, and behavioral data"; "Health coaches were virtual diabetes educators who regularly reviewed patient data, providing personalized feedback during each interaction, and also responded to patient queries" |
| Magnani et al., 2017 | **Self-monitoring of Behavior;** Goal-setting (behavioral)**; Feedback on Behavior; Instruction on how to perform a behavior** | Inferred from text | self-monitoring:  “Symptoms consisted of assessment of frequency and severity of shortness of breath, chest pain or discomfort, fatigue, and palpitations or the sensation of a racing or irregular heartbeat. Adherence content focused on common challenges to medication adherence, such as forgetfulness, affordability, access to medications, transportation, and the patient-physician relationship, along with general strategies to address these obstacles.” ; Kardia mobile heart rhythm monitor (AliveCor, Inc, Mountain  View, CA) or Kardia for simplicity, allows individuals to  monitor heart rate and rhythm with the help of a smartphone  with results being uploaded for centralized review.”  Feedback on behavior: “The relational agent application and the Kardia monitor transmitted their data to a central server for monitoring, data collection, and analysis.”; Kardia mobile heart rhythm monitor (AliveCor, Inc, Mountain View, CA) or Kardia for simplicity, allows individuals to monitor heart rate and rhythm with the help of a smartphone with results being uploaded for centralized review.”  Goal-setting: “Activation content entailed articulating goals of care”  Instruction on how to perform a behavior,  “Adherence content  focused on common challenges to medication adherence, such  as forgetfulness, affordability, access to medications,  transportation, and the patient-physician relationship, along  with general strategies to address these obstacles.”  Education or information provision (5.1): “Education spanned the causes of AF and its associated risk factors, treatments, and adverse events. |
| Qiu et al., 2021 | Self-monitoring of Behavior; Instruction on how to perform a behavior; Instruction on how to perform a behavior; **Prompts/cues** | Inferred from text | Self-monitoring of behavior & Feedback on behavior: “Nurse AMIE collects daily symptoms (sleep, distress, fatigue, pain) and status (number of steps) data from participants. It then uses the collected data to recommend a self-management intervention strategy.”  Instruction on how to perform a behavior: exercise videos demonstrating physical activities; “Movements (exercise videos), Coping with Symptoms (educational videos), Guided Relaxation (meditation audio), Soothing Music, Nutrition (daily nutrition tips and recipes)”  Prompts/cues: daily check-in reminders and intervention suggestions (“Received today’s intervention”?). |
| Roca et al., 2021 | **Self-monitoring of Behavior; Feedback on Behavior; Prompts/cues** | Inferred from text | Prompts/cues: "Patients can add medication reminders; Medication reminders are sent a maximum of three times per programmed intake."  Feedback on behavior: "The virtual assistant gives a summary of the adherence every week."  Self-monitoring: " The two main functionalities included are “medication option”, where patients can add medication and medication reminders, and “appointment option”, where patients  can add an appointment with their healthcare professionals and conﬁgure appointment notiﬁcations." |
| Sakane et al., 2023 | **Self-monitoring of Behavior;** Goal-setting (behavioral)**; Feedback on Behavior; Instruction on how to perform a behavior** | Explicitly reported: self-monitoring 2.3, goal-setting 1.1.  Inferred from text: feedback 2.2., education/ information provision 5.1, instruction on how to perform a behavior 4.1 | Figure 1. Screenshot of KENPO-system  Self-monitoring of behavior, Feedback on behavior, instruction on how to perform a behavior: “chatbot-supported feedback and information provision combined with a self-monitoring tool (weight, steps, and blood pressure).” “Chatbot-supported feedback of app data and sign of weight regain”  Goal-setting (behavioral): Participants were encouraged to set personalized goals related to weight loss and healthy behaviors based on their initial health checkup and personality traits. (e.g., Weight loss goal, daily step goal in Figure 1), Textbox 1 (“Setting weight loss goal”)  Instruction on how to perform a behavior: The app delivered daily health quizzes, including guidance on healthy eating habits (e.g., eating vegetables before rice) and physical activity strategies, Texbox 1 (“Quiz on Health”) |
| Schläpfer et al., 2024 | **Goal-setting: (behavioral & outcome), problem-solving, action planning, review behavioral goals, discrepancy between current behavior and goal, positive reframing, normalize difficulty, emphasize autonomy** | Explicitly reported | 39 BCTs, specified in Appendix as part of the “coaching sessions”. |
| Schlieter et al., 2017 | **Self-monitoring of Behavior; Feedback on Behavior; Instruction on how to perform a behavior; Prompts/cues** | Explicitly reported, though implementation inferred from quotes | Explicitly reported: "the behavior change techniques of  self-monitoring, feedback, prompts, and goal-setting were  most frequently used and associated with positive effect"  Self-monitoring of behavior (MR2.2); Feedback on behavior (MR2.3)  : "Self-monitoring of vital signs and activities and feedback by the coach”.  Instruction on how to perform a behavior (MR2.4): "E-learning content is provided to foster healthy behaviors."  Prompts/cues (MR2.3): "Social and dialog support for structuring activities using reminders”. |
| Shamekhi et al., 2017 | **Self-monitoring of Behavior; Goal-setting (behavioral); Instruction on how to perform a behavior; adding objects to the environment** | Inferred from text | Goal-setting: “final coordinated session is allocated to behavioral goal setting, wherein patients can set new goals, review their goals or check their goals”.  Self-monitoring: “They are then asked… how their practices are going”.  Instruction on how to perform a behavior: “Gabby… walks patients through practice sessions… guides users through several meditation practices and a yoga session.”;“Gabby also reviews educational information on nutrition, physical activity, pain, stress, sleep, and depression.”, “Patients… have the option of completing practice activities… meditation, self-massage, acupressure, and yoga.”  Adding objects to the environment: “Calming music was also added… Gabby selects a random picture for each meditation session from a set of 15 pictures.” |
| Ter Stal et al., 2021 | Self-monitoring of Behavior; **Feedback on Behavior**; Instruction on how to perform a behavior; Education or information provision; **Prompts/cues** | Explicitly reported (as dialogue features, not BCTs per se though) | Self-monitoring of behavior: daily symptom diary, weight monitoring. (Textbox 1)  Feedback on behavior: Inhaler feedback on Inhalation adherence, technique. (Textbox 1)  Prompts/cues: action reminders, weighing, questionnaires. (Textbox 1)  Instruction on how to perform a behavior: …set of breathing, relaxation, and physical exercises, accompanied by videos and explanation in text. (Textbox 1) |
| Sweidan et al., 2023 | Self-monitoring of Behavior**; Instruction on how to perform a behavior**; Social support; Prompts/cues | Inferred from text | Prompts/Cues: Reminders for medications and daily tasks via smartphone app notifications ("...reminding the patient of medication doses and times.")  Instruction on how to perform a behavior: General knowledge about Alzheimer’s disease delivered through a chatbot ("...an informative chatbot that responds to the most common questions any patient may have.")  Social support: Interaction with caregivers and doctors through messages and chat feature ("...allows patients to communicate with caregivers and doctors through messages.")  Self-monitoring: Patients keep track of daily life tasks and medications via app interface ("keeping track of daily life tasks.") |
| Wonggom et al., 2020 | **Instruction on how to perform a behavior; Prompts/cues** | Inferred from text | Instruction on how to perform a behavior: “educational quizzes, content of the avatar app includes understanding heart failure, looking after yourself, things to do every day, emergency action plans.”  Prompts: “app acting as a reminder..” |
| Zisis et al., 2021 | **Self-monitoring of Behavior; Feedback on Behavior; Instruction on how to perform a behavior; Prompts/cues** | Inferred from text | Instruction on how to perform a behavior, Prompts and cues, feedback & self-monitoring: “Program engages daily with the patients in order to provide education about HF and prompts related to HF management. It reminds them to weigh themselves daily and to record the result, with recommendations to notify their nurse or doctor if >_2 kg is gained within 2 days." |

*BCTs delivered by virtual agent component of the intervention marked in **bold**
