# Supplementary material for: Mapping behavior change techniques and health data combinations in virtual agents for chronic condition management: A systematic scoping review
Source: PLOS Digit Health. 2026 Jul 28;5(7):e0001604. doi: 10.1371/journal.pdig.0001604 (PMC13411939; doi:10.1371/journal.pdig.0001604)
Supplement: Attachment — Submitted filename: Response to Reviewers.docx [file pdig.0001604.s006.docx]

**Rebuttal Letter**

1. **Response to the Editor**

Thank you for submitting your manuscript, which addresses an important and timely topic with strong potential to advance the design and reporting of virtual agent interventions for chronic condition management. To strengthen its clarity, rigour, and practical value, please clarify the conceptual focus on personalisation and consider adding a simple model illustrating relationships among key elements; enhance methodological transparency by detailing the search strategy, justifying the exclusion of grey literature, and aligning BCT coding strictly with BCTTv1 taxonomy while explaining coder training and reliability checks; reduce redundancy between overlapping sections and clarify how thematic and structured analyses relate; position the work more explicitly against prior reviews (e.g., Martinengo et al., Jiang et al.) and temper novelty claims; and offer more concrete guidance in the conclusion, such as structured mapping and reporting standards. Minor revisions include consistent terminology, defining abbreviations, and clarifying scope regarding chronic conditions. Addressing these points will significantly improve the manuscript’s impact and usability.

We thank the editor and reviewers for their constructive, detailed and encouraging feedback! We have revised the manuscript in response, to increase clarity and scientific rigor. Major changes are outlined below. Detailed point-by-point responses to both reviewers are provided in the two tables below as following:

- **Table 1** on page 3-16 – Paula Voorheis
- **Table 2** on page 17-24 – Graziella Zangger

Major changes include:

- A clarification of the conceptual focus of the review in the introduction by explicitly framing virtual agent-delivered interventions as systems composed of three interacting components: health data, behavior change techniques (BCTs), and delivery channels.

We further clarified that personalization is not considered a separate variable but operationalized within this framework as the integration and use of health data inputs.

- A conceptual diagram (**Figure 1**) has been added to illustrate the relationships between the three components to make the analytical logic more explicit.
- The description of the search strategy has been expanded to clarify that searches were conducted from database inception until September 10^th^, 2024, and additional details on keyword selection were provided.
- We clarified that gray literature was not included, as this review focused on empirically evaluated virtual agent-delivered interventions requiring sufficient methodological detail for structured extraction of health data, BCTs and delivery channels. This has also been acknowledged as a limitation.
- The BCT coding procedure has been refined and described in more detail. We ensured alignment with the BCTTv1 taxonomy, removed non-taxonomy constructs, and clarified how BCTs were inferred. We also added information on coder expertise, training, and coding procedures.
- We added a study-level overview of BCT coding with supporting quotes from the included papers (**Supplementary Table 5**), detailing how each BCT was identified from intervention descriptions. This enhances transparency and allows readers to trace how BCTs were inferred and mapped to BCTTv1 categories.
- We clarified the relationship between the qualitative content analysis of design rationales and the structured extraction of intervention components in the Discussion, highlighting their complementary roles.
- Redundancy between the sections on “Use of combinations” (RQ2) and “Patterns and contexts” (RQ3) was reduced by streamlining descriptive text.
- The manuscript has been more explicitly positioned in relation to prior work of Martinengo et al. (2022) and Jiang et al. (2024). We clarified how this review extends prior research by focusing specifically on chronic physical conditions and by systematically mapping intervention architecture (BCT-health data-delivery channel combinations).
- Statements regarding trends (e.g., increasing complexity) were tempered and more clearly grounded in the findings.
- The conclusion has been strengthened to provide more concrete guidance, including recommendations for:
  - Clearer reporting of BCTs using BCTTv1 terminology
  - Structured mapping of BCT-health data-delivery relationships
  - Improved reporting of virtual agent intervention architectures
- Minor revisions have been made by making terminology consistent throughout the manuscript, defining abbreviations upon first use and clarifying the scope regarding “chronic conditions” early on.

**Response to Reviewer 1 – Paula Voorheis**

| **#** | **Line** | **Reviewer Comment** | **Response** | **Manuscript Changes** |
| --- | --- | --- | --- | --- |
| Abstract | | | | |
| 1. | 35 | Define virtual agents briefly if you can. | We agree a brief definition improves clarity for readers who may be less familiar with the term. We have added the definition. | (35) Digital health technologies, particularly virtual agents, which are computer-generated characters simulating human-like interactions through verbal and nonverbal cues, offer new ways to provide personalized, scalable, and continuous support. |
| 2. | 36-37 | Do you mean the ways in which distinct components within chronic care interventions or within virtual agent interventions that support chronic care…? Be precise here. | Thank you for pointing out this ambiguity. We agree that the phrasing was too broad. We intended to refer specifically to components within virtual agent-delivered interventions that support chronic condition management, rather than chronic care interventions more broadly. We have revised the sentence to clarify this distinction. | (37) However, the ways in which distinct components within such digital health technologies, including those used in chronic care interventions, are chosen and combined remain underreported. |
| 3. | 41 | Comprehensive search can probably just be search. Can a search every truly be comprehensive? Also, what about gray literature? | Thank you for this thoughtful comment. We agree that the term “comprehensive” may overstate the scope of any database search and have thus revised the wording to simply “search”. Regarding the gray literature, our objective was to map empirically validated virtual agent-delivered interventions that reported longitudinal testing in real-world settings. We therefore restricted inclusion to peer-reviewed empirical studies to ensure sufficient methodological detail, intervention transparency, and outcome reporting. We have clarified this rationale in the methods section and acknowledged this as a limitation in the discussion section. | (43 & 184) Replaced “comprehensive” search with “search”.  (203) Given the focus on empirically evaluated virtual agent–delivered interventions with longitudinal or real-world testing, gray literature was excluded to ensure sufficient methodological detail and transparency for the structured extraction of intervention components.  (562) Finally, the search was restricted to peer-reviewed publications and did not include gray literature, which may have excluded developments reported in industry reports or non-academic sources. |
| 4. | 43 | You say the first step, but no other steps are listed. | Thank you for pointing this out. We agree that referring to a first step implies additional steps that were not described as such in the abstract. We have therefore revised the wording to remove this sequencing language for clarity. | (45) Our analysis examined the rationales reported by authors (…) |
| 5. | 44 | By intervention design do you mean virtual agent design? | Thank you for this important clarification request. While all included studies involved virtual agent-delivered interventions, our use of the term “intervention design” refers to the overall design of the intervention in which the virtual agent was embedded (including behavior change strategies, data integration, and delivery channels), rather than solely the technical design of the virtual agent itself. We have revised the wording to make this distinction clearer. | (45) (…)rationales reported by authors underlying the design of the virtual agent-delivered intervention as a whole. |
| Text | | | | |
| 1. | 76 | Provide reference for “which is essential for behaviour change” | Thank you for this suggestion. We agree that this statement requires supporting evidence and have added an appropriate reference to substantiate the importance of sustained, personalized feedback and coaching for behavior change. | (78) A reference (Mair et al., 2023) has been added to support this claim. |
| 2. | 81-82 | People who aren’t experts won’t know what these different types mean or how they are different from each other. | Thank you for this helpful observation. We agree that the distinctions between terms required clarification. Based on existing literature, we have revised the paragraph to reflect the hierarchical relationship between these terms: conversational agents (CAs) as the broader category, embodied conversational agents (ECAs) as a subset of CAs characterized by visual embodiment, and “virtual coaches” as a functional role often implemented using CAs or ECAs in health behavior interventions. This revision improves conceptual clarity while maintaining conciseness. | (83) In lifestyle-based interventions, virtual agents commonly take the form of conversational agents (CAs), which are text- or voice-based dialogue systems, or embodied conversational agents (ECAs), which are animated, visually represented agents capable of simulating face-to-face interaction. In health contexts, such agents are often designed to function as virtual coaches, providing structured guidance and support for behavior change. They are used to support a range of health behaviors such as dietary intake, physical activity, stress, sleep, and dementia care. |
| 3. | 105-107 | Are there other reviews on personalization to reference? | Thank you for this suggestion. Although to the best of our knowledge no prior reviews have specifically addressed personalization in CAs, we have now added a reference to a broader review on personalization in health behavior change applications, which highlights AI assistants and chatbots as promising technologies for advancing personalization delivery. We retain the cancer-focused examples to illustrate applied implementation and clarify how the present review builds on this work by examining how personalization is operationalized within virtual agent-delivered interventions. | (112)(…) though gaps remain, particularly in understanding how different personalization strategies work individually and in combination. Still, prior reviews have highlighted emerging AI assistants and chatbots as promising technologies for advancing personalization delivery (Kankanhalli et al., 2021). |
| 4. | 110 | it’s not clear what you mean by “this” in “… examples how THIS is applied in virtual agent delivered interventions”… Can you be more precise?; | Thank you for pointing out these related observations of ambiguity (comment 4. & 5.). We have revised the sentence to explicitly define how we conceptualize personalization in the context of virtual agent-delivered interventions. Specifically, we clarify that in this review, personalization is not considered a separate variable but conceptualized as the integration of health data inputs, which are commonly operationalized by the inclusion of structured strategies like BCTs. | (116) Building on this, this review conceptualizes personalization in virtual agent-delivered chronic care interventions as the integration of health data inputs. This can involve information such as medical history, symptoms, biometric indicators, treatment records, and lifestyle factors, either directly provided by the patient or inferred from their input. |
| 5. | 111-124 | It seems like, at this point in the introduction, the point of this review is to understand personalization approaches are applied to virtual agent delivered approaches…. | Addressed above in comment 4. | Addressed above in comment 4. |
| 6. | 110-125 | Maybe these paragraphs could be a table? | Thank you for this suggestion. We agree that structured categorization is helpful. A detailed operationalization of the different health data types is provided in **Table 1** (line 246) in the Methods section, and we now explicitly refer to this table in the Introduction when describing the health data. In the Introduction, we retain a brief narrative description to support conceptual flow and reader orientation before outlining the study’s analytical framework. | (120) For the purpose of the review, a distinction is made between different types of health data (see **Table 1** in the Methods section for a detailed overview). (…) |
| 7. | 132-135 | So far these lines seem to communicate the central focus of the paper: how (a) BCTs and personalized health data are (b) delivered through different delivery channels, within (c) virtual agents, to (d) impact behaviour change outcomes… I would make this crystal clear and use the same language in the abstract and all of the text. I think you reframe your objective in the review several times in slightly different ways, which is confusing. In the abstract your language is: to map how (a) BCTs, health data, and delivery channels are (b) rationalized and (b) integrated into (d) virtual agent delivered interventions for chronic condition management. Just be consistent throughout. | Thank you for this helpful observation. To improve clarity and consistency, we have aligned the wording throughout the manuscript. Specifically, we now consistently state that the review maps how behavior change techniques (BCTs), health data types, and delivery channels are rationalized, combined, and applied in virtual agent-delivered interventions for chronic condition management. The abstract, introduction, methods, and discussion have been revised accordingly. | (39) Abstract: We conducted a systematic scoping review to map how behavior change techniques (BCTs), health data types, and delivery channels are rationalized, combined, and applied in virtual agent-delivered interventions for chronic condition management.  (162) Introduction RQs:   (1) What theoretical or practice-based rationales do authors provide for the use and combination of BCTs, health data types, and delivery channels in virtual agent-delivered interventions for chronic condition management? (2) What BCTs are used in combination with specific types of health data and delivery channels in virtual agent-delivered interventions for chronic condition management? (3) How prevalent are different combinations of BCTs, health data types and delivery channels in virtual agent-delivered interventions for chronic conditions and in what contexts are they applied (e.g., study population, intervention aim, setting)?   (251) Methods (Data Synthesis)  In line with the review objective, said analysis aimed to map how BCTs, health data types, and delivery channels are rationalized, combined, and applied in virtual agent-delivered interventions for chronic condition management.  (425) Discussion  This systematic scoping review mapped how BCTs, health data types, and delivery channels were rationalized, combined, and applied in virtual agent-delivered interventions for chronic condition management across 21 studies. |
| 8. | 141 | RQ1 - I feel like when you introduce RQ1 about justification/rationale it comes as a bit of a surprise as you didn’t talk about the issue of rationale and justification in the introduction very much. Is there previous research that has tried to map rationale and how rationales are categorized? | Thank you for this important observation. We agree that the motivation for examining rationale and justification required clearer framing in the introduction. We have now added a paragraph summarizing prior findings that demonstrate inconsistent reporting of theoretical foundations in conversational agent and digital health interventions in the chronic care context, as well as evidence suggesting that stronger theoretical grounding is associated with improved intervention outcomes (e.g., in other contexts such as eating disorder interventions). In addition, we clarified that there is limited prior work systematically categorizing design rationales in this context, which motivated our exploratory approach to mapping these rationales. This provides clearer justification for RQ1. | (143) In addition, the theoretical and practical rationales underlying these design choices are often insufficiently articulated. Reviews of conversational agents in chronic healthcare have found that a substantial proportion of studies are not explicitly informed by behavior change theory (Martinengo et al. 2022; Salas-Groves et al., 2023). At the same time, evidence from other domains suggests that stronger theoretical grounding may enhance intervention effectiveness (Webb et al., 2010). For example, a meta-analysis of digital eating disorder interventions found that interventions with higher theory coding scheme scores demonstrated significantly greater improvements in outcomes compared to less theory-informed interventions (Thomas et al., 2024). Together, these findings highlight the importance of examining how design decisions in virtual agent-delivered chronic care interventions are justified. However, limited prior work has systematically categorized how such rationales are articulated in virtual agent-delivered interventions, indicating a need for exploratory mapping approaches. |
| 9. | 144 | RQ2 – Based on this question it seems like you are looking at BCTs by their (a) association with certain types of health data, and (b) with certain types of delivery channels? I feel like this organization didn’t come across in your introduction. Here it makes it seem like BCTs are the foundational focus and you will describe them by these 2 different ways. As I said above, just be consistent. This also makes me think some sort of visual diagram might be helpful to outline the logic of this review? | Thank you for this suggestion. We have clarified the analytical logic of the review in the introduction by explicitly describing virtual agent-delivered interventions as systems composed of three interacting components: health data inputs, BCTs, and delivery channels. In addition, we added a conceptual diagram (Figure 1) to visually illustrate how these components relate to one another within virtual agent interventions. | (154) In this systematic scoping review, virtual agent-delivered interventions are conceptualized as systems composed of three interacting components: health data inputs, BCTs, and delivery channels. **Figure 1** illustrates how virtual-agent delivered interventions can be conceptualized as the integration of these three interacting components. |
| 10. | 176 | Eligibility Criteria. For “Utilize BCTs”, I think this might require more explanation. How would you decide on an inferred BCT? Also, if they just referenced behavioural theory in the intro but didn’t use a BCT, how would this be treated? Did you have an expert in behavioural science? Also I think in the introduction you mentioned behaviour change outcomes only, not health outcomes and user experience outcomes. Consider outlining somewhere in your methods what the authorship expertise is and how you brought your own experiences into the eventual coding of things… | Thank you for these helpful comments (also comment 11). We have clarified the procedure used to identify behavior change techniques (BCTs) when they were not explicitly reported. Specifically, we added a description in the methods explaining that intervention descriptions were mapped to the behavior change technique taxonomy v1 (BCTTv1), and that only techniques that could be clearly matched to taxonomy definitions were coded. Interventions that only referenced behavioral theory without describing identifiable BCT components were not included. We also added a brief description of the research team’s expertise relevant to this coding process, and how the main extraction categories were defined. | (206) **Textbox 1: Eligibility Criteria**  Intervention types:  - must incorporate behavior change techniques (BCTs), either explicitly reported or identifiable based on the intervention description using the BCT Taxonomy v1.  (234) BCT identification and coding were performed by the first author, who has a background and experience with behavior change intervention design using the BCT Taxonomy and the CeHRes Roadmap. Coding decisions were reviewed and discussed with a third co-author (SK), with expertise in eHealth intervention design to ensure consistency and validity. Any uncertainties in classification were resolved through discussion and consensus. The main extraction categories (e.g., BCTs, health data, delivery channels) were defined a priori based on the study objectives and existing frameworks, while subcategories were refined iteratively during the extraction process (e.g., differentiation between health data input and output). |
| 11. | 202 | Data extraction items. With BCTs, how did you infer BCTs from intervention descriptions? As stated above, this usually requires behavioural science expertise on the authorship team. For other categories, can you explain if the categories were decided on a priori or as extraction was happening? (e.g., health data integration). | Addressed in comment 10 above. | Addressed in comment 10 above. |
| 12. | 207 | Make sure your introduction text aligns with this table. | Thank you for pointing this out! We reviewed the introduction text and the health data table to ensure consistent terminology. Specifically, we revised the introduction to use the term “adherence data” instead of “adherence patterns” to match the table. In addition, we clarified the definition of subjective health data in the table so that it aligns with the wording used in the introduction. | (128)(…) user preference data [24], and adherence data [25], can further inform how virtual agents tailor support.  (245) Table 1. Health data types  Subjective health data description: Self-reported data regarding a person’s health status, perceived symptoms, or behaviors. |
| 13. | 217 | If you are referencing the narrative thematic analysis method, you will need to reference this with a methodological paper and explain your application of the referenced method with a bit more detail. | Thank you for this helpful comment. We clarified the methodological description of the analysis used to categorize intervention rationales. Specifically, we now explicitly describe this step (a qualitative content analysis) and added the methodological reference used (Elo & Kyngäs, 2008). The methods section was expanded to explain how intervention rationales were coded and grouped into categories. | (248) Data synthesis followed the same methodological frameworks (Arksey and O’Malley, JBI, PRISMA-ScR) and combined conducting a descriptive and qualitative content analysis of the extracted data. (…)   (256) A qualitative content analysis was conducted to examine how authors justified intervention design choices, following the approach described by Elo and Kyngäs (2008). Descriptions of the rationales for intervention design choices reported in the included studies were reviewed and coded by the first reviewer. Codes capturing different types of justification were iteratively grouped into broader categories through comparison across studies. To ensure consistency in interpretation, each study’s rationale was categorized using a structured coding scheme developed inductively from the data (…). |
| 14. | 220 | This coding scheme is very interesting! Do you mean the study’s rationale for building the virtual agent? If so, its hard for me to imagine what some of these rationale categories would look like… For instance, what do you mean by practice-driven rationale… Can you be a bit more clear with your examples for each categorization? Hard for me to picture. | Thank you for these helpful comments on further clarification on our side (also addressing comment 16.). We clarified the meaning and scope of the “intervention design rationale” coding scheme. Specifically, we added a definition of “rationale” in the Methods section (data synthesis). We also expanded the description and examples of the rationale categories, particularly for the practice-driven category, to better illustrate what types of justifications were included. | (220) (…) Here, “rationale” refers to explanations provided by study authors for selecting intervention components, or broader design approaches (e.g., theoretical grounding or prior empirical evidence). (…) Five rationale categories were identified: *theory-driven* (e.g., intervention design grounded in behavioral theories such as Self-Determination Theory or COM-B), *practice-driven* (e.g., design decisions informed by usability considerations, patient feedback, or clinical implementation needs), *empirically-driven* (based on findings from prior empirical studies without explicit theoretical grounding), *mixed* (combining multiple of the former categories), and *not explicitly stated*. |
| 15. | 268-274 | This is really interesting and consistent with other reviews I have seen. I would love you to discuss in the discussion section the implications of rationale only provided at the intervention level and not for specific choices made about BCTs, health data, and delivery channels. (You do this! So ignore). |  |  |
| 16. | 280 | Intervention Design Rationale Table. As I stated earlier, it would be nice to have some more details about these categories (what they include and exclude). | Addressed in comment 14. | Addressed in comment 14. |
| 17. | 280 | It also appears some papers might have discussed rationales at a more specific level than the intervention as a whole, as you describe, for example, that Roca et al had “functionalities developed iteratively with nurses”. For papers that did go into more specific rationales beyond the broad intervention level, did you categorize these? …. I see later in the text you say “the rationale for use of individual intervention components like health data types and delivery channels was often implied through functionality or usability features, but not systematically articulated.” I think I see where you are going but it’s not entirely clear the distinctions here. Maybe just a bit more clarity would help. | Thank you for this helpful observation. We revisited the extracted rationale statements and clarified this point in the Results section. While some studies described aspects of the intervention development process (e.g., Roca et al.), these descriptions generally did not constitute explicit rationales for individual components such as specific BCTs, health data inputs, or delivery channels. Instead, rationales were typically reported at the level of the broader intervention design, and were categorized accordingly in our analysis. | (325) (…) Although some studies described aspects of the intervention development process (e.g., “functionalities iteratively developed with nurses”), these descriptions generally did not constitute explicit rationales for individual components. (…) |
| 18. | 281-291 | I am not sure how much more detail these paragraphs are providing beyond what is already in the tables? I talk about this later, but if you are worried about word count, try to be less repetitive in your text when its already in the tables. | Thank you for this suggestion. To reduce repetition between the text and **Table 3**, we removed the paragraph that listed multiple study-specific examples of rationale categories again. Instead, the Results section now focuses on interpreting the patterns shown in the table, while the table itself provides the detailed study-level information. The subsequent paragraph was retained as it highlights an additional finding regarding the limited reporting of interconnections between BCTs, health data, and delivery channels. | deleted (paragraph before 332) |
| 19. | 292 | I thought you were reporting connections in another section/ addressing another RQ besides RQ1. This paragraph seems misplaced? Or maybe it just needs to be more obvious you were reviewing rationales for connections? | Thank you for this observation. We clarified that this paragraph refers specifically to *rationales for interconnections* between BCTs, health data, and delivery channels rather than to the mapping of these components themselves. To avoid confusion with the subsequent analysis addressing another research question, we revised the opening sentence of the paragraph accordingly. | (332) Only a small subset of studies (n=3, 14.3 %) provided an explicit rationale discussing interconnections between BCTs, health data, and delivery channels (Schläpfer et al., Gomez et al., and Gong et al.). |
| 20. | 303 | Can you put the references for the 3 studies that explicitly reported BCTs? | Thank you for this helpful comment. We have added the references to the 3 studies for clarity. | (345) (Sakane et al., 2023; Schläpfer et al., 2024 & Schlieter et al., 2024) |
| 21. | 311-312 | I think this distinction is important and could be make clearer throughout the entire paper, as I am assuming many of the interventions had virtual agent components and non-virtual agent components. It makes me think that your section above on intervention rationale should either (a) just focus on the rationale for virtual agents or (b) be very clear that it was the entire intervention which included some parts that included a virtual agent. | Thank you for this helpful suggestion. We clarified for research question 1, that reported design rationales typically referred to the overall intervention design, which in most cases included a virtual agent as one component of a broader digital intervention. | (268) While research question 1 aimed to examine the rationale authors provided for the use or combinations of BCTs, health data, and delivery channels, most studies framed their justification at the level of the overall intervention design and purpose, which typically included a virtual agent as one component of a broader digital intervention. |
| 22. | 313 | Table 4 – For BCTs used, do you have a supplementary appendix you could add that outlines the inferring of these BCTs and what/how you coded as “self-monitoring” ,“feedback” etc. I think this would be helpful for readers to be transparent about how different features were coded as specific BCTs. | Thank you for your helpful suggestion. To increase transparency of the BCT coding process, we have added a supplementary table (**S5 BCT coding evidence**) providing evidence on how intervention descriptions were mapped to the BCTTv1 categories. The supplementary table S5 provides evidence on how intervention descriptions were mapped to the BCTTv1 categories. The table includes the coded BCTs per study, whether they were explicitly reported in text or inferred by the authors, and excerpts from the original study descriptions that informed the coding decisions. | [added: supplementary file **S5 BCT coding evidence**]  (353) Examples of how intervention features were mapped to BCTTv1 categories are provided in **S5 BCT coding evidence**. |
| 23. | 316-356 | These results are very interesting and answered some initial concerns I had about outlining how you categorized features of the virtual agents into BCT categories. I wonder if it would be easier for the reader if this section had a table that outlined the (1) the BCT, (2) the operationalization of the BCTs in the virtual agent (and out), (3) the delivery channels of the operationalizations of the BCT… or something like this... I feel like this written section has a lot of useful synthesis and just providing a table at the study level doesn’t do enough. | Thank you for this suggestion. We added an additional synthesis table (**Table 5**) summarizing how commonly used BCTs were operationalized within the virtual agent interventions, including the associated health data inputs and delivery channels and mechanisms. This table complements the study-level overview presented in **Table 5** and helps readers more easily interpret the cross-study patterns described in text. | (357) To facilitate interpretation of the cross-study patterns described below, a synthesis summary of virtual agent-delivered intervention features is provided in **Table 5**. |
| 24. | 358 | Just to confirm these combination types and frequencies are just for the virtual agents or the interventions as a whole? I think I keep getting confused what level the results are referring to in the written text. | Thank you for this comment, we understand the confusion. We clarified in the manuscript that the combinations reported in this section refer to BCTs, health data, and delivery channels delivered by the virtual agent components identified in Objective 2. The section titles for both objective 2 and 3 have been revised to make the level of analysis more explicit. | (342) Section title Objective 2:  Use of virtual agent-delivered BCT, health data & delivery channel combinations  (402) Patterns & contexts of virtual agent-delivered BCT, health data, and delivery channel combinations |
| 25. | 358-403 | I am a bit confused on the counting in these clusters in terms of studies that could be double counted. For example, for the 10 studies that included self-monitoring, feedback, and instruction, where these 10 also all included in the count of studies with self-monitoring and feedback? Or are these clusters distinct? | Thank you for this helpful observation. The clusters described in this section are not mutually exclusive because many interventions implemented multiple BCTs simultaneously. As a result, individual studies may appear in more than one cluster. We clarified this in the manuscript by adding a sentence at the beginning of the section to explain that the reported combinations represent overlapping patterns. | (406) (…) Because all interventions implemented multiple BCTs simultaneously, the clusters described below are not mutually exclusive, and individual studies may appear in more than one cluster. |
| 26. | 404 Table 6 | I think including prevalence more obviously in this table would help, as this was within RQ3s main objective. | Thank you for this helpful suggestion. To make the prevalence of the identified patterns clear, we added a “prevalence (n, %) column to Table 5, to allow for a more direct overview for the reader. | (**Table 6**) Added “prevalence” column next to common contexts. |
| 27. | 404 | Overall, I feel like there is a lot of dense text which is often very duplicative of the tables and maybe adds more confusion than clarity. It’s hard to know when I am reading new, important information in the text that is not in the tables or when it is just easily repeated in the tables. This is a theme throughout your results sections. | Thank you for this observation. To reduce redundancy between the text and tables, we shortened the RQ3 results section text and focused the narrative on the key patterns and prevalence of the identified BCT-health data-delivery channel combinations. Detailed descriptions of the operationalization of these combinations are now presented primarily in Table 6, while the text highlights the overarching patterns and contextual interpretation. | (404) Building on the agent-delivered BCT, health data, and delivery channel combinations identified in Objective 2 (see **Table 4 and 5**), this section explores the contexts in which these combinations were applied, including chronic condition focus, intervention aim and setting. The operationalization of these combinations, including the types of health data used and delivery mechanisms, is summarized in **Table 6.**  A closer look at the health data suggests tentative condition-specific patterns across the interventions. Cancer or chronic pain studies (n=8, 38.1 %) relied more heavily on subjective user-centred data (e.g., symptoms, side-effects, mood), with objective data (e.g., step counts) adherence data (e.g., exercise completion) and/or user preference data (e.g., preferred timing of reminders) as additional inputs layered on top. By contrast, cardiovascular and metabolic studies more often combined subjective data on self-management behaviors and objective physiological measures (e.g., blood pressure, weight, glucose etc.), supplemented with adherence data (e.g., measurement completion). Beyond this distinction, no consistent pattern emerged linking specific BCT-data-delivery combinations to particular conditions or system types. |
| 28. | 434-426 | This is a key finding, great! | We thank the reviewer for this encouraging comment! |  |
| 29. | 451-454 | Very important! I actually think you are understating an important implication of your work by referencing Eaton who observed that more BCTs were better… Your study is starting to get at this idea that not just more is better, but certain combinations of cohesive intervention strategies that can be delivered together in a meaningful tandem might make the difference… where the whole is greater (?) than the sum of the individual parts and something new might be created… Obviously, you can’t prove this in the review, but I wonder if there is any more literature not only on “more BCTs = better”, but “BCT combinations = better”… I am not up to date on this literature, but I am curious… | Thank you for this insightful suggestion. We agree that our findings point toward the importance of cohesive combinations of intervention components, rather than simply the number of BCTs used. We therefore slightly expanded this section of the Discussion accordingly. At the same time, to our knowledge, there is currently limited empirical literature that systematically examines (or has found evidence on) the effectiveness of specific BCT combinations. | (455) However, our findings suggest that not only the number of BCTs, but also the way they are combined and embedded within system interactions may matter. In many of the reviewed studies, BCTs such as feedback, instruction on how to perform a behavior and prompts/cues were integrated within a single interaction flow, indicating that these elements may operate synergistically rather than as isolated components. |
| 30. | 460 | So BCTs can BE system functionalities themselves. I think the conversation in this paragraph makes me think about whether its actually a useful practice to take an intervention and separate it out into individual BCTs when the reality of the human experience of an intervention is really multiple of these simultaneously, creating something new altogether. Our brains aren’t separately receiving these BCTs anymore… (I wonder if there are any cognitive studies on this…) What implications does this result have for mechanisms of action being leveraged and the field of applied behavioural science more generally I wonder? | Thank you for this insightful reflection. We agree that the integration of behavioral strategies within interactive digital systems raises important conceptual questions for applied behavioral science. Although we cannot draw direct conclusions on this based on the current review, we do agree this implies the integration of multiple BCTs within a single interaction flow may shape mechanisms of action in ways that are not fully captured when techniques are treated as distinct components. In response, we added a sentence in this part of the Discussion, highlighting that BCTs in virtual agent interventions may function less as discrete components and more as integrated interaction processes within digital systems. | (461) This raises questions about whether BCTs should be conceptualized as discrete intervention components or as integrated interaction processes within digital systems, where multiple behavioral strategies may be experienced simultaneously by users. |
| 31. | 473-475 | Hm, I wonder if this conclusion is correct… Because if we did magically figure out recipes for BCT, data, channel combinations for different chronic conditions, this MIGHT enable evidence-based virtual agent development, but it also wouldn’t leave room for creativity and design thinking related “evidence” to be included in future intervention design. We would end up with a phone book or rule book for design which doesn’t really encompass realities of human experiences and constant political, cultural, or social changes. I don’t know if a static rulebook could really ever be made… | Thank you for this thoughtful reflection. We agree that identifying rigid “recipes” for intervention design would indeed not capture the dynamic and context-dependent nature of digital health interventions. In response, we revised the sentence to emphasize the need for research that examines how combinations of BCTs, health data, and delivery channels function across contexts, rather than suggesting fixed design rules at this point. | (506). This suggests that beyond improving reporting, the field should move toward systematically examining how combinations of BCTs, health data, and delivery channels function across different chronic care contexts. Such work would help inform more transparent and evidence-based virtual agent intervention design, while still allowing room for contextual adaptation and design innovation. Rather than identifying fixed design “recipes”, this may support the development of mechanism-level insights that can be adapted and refined to specific target groups and settings. |
| 32. | 476-477 | Interesting! Would love to know your thoughts on this re: next steps and future directions… | Thank you for this comment, we agree that this has important implications for future research. We therefore expanded the future directions section to highlight the need for approaches that examine how behavioral strategies interact with system functionalities and health data flows within digital health systems. | (525) To support such developments, future work should adopt standardized taxonomies like the BCTTv1 and provide clear links between intervention components. At the same time, future research may benefit from approaches that examine how behavioral strategies interact with system functionalities and health data flows within digital health systems, rather than treating individual techniques as isolated intervention elements. Developing visual or modular mapping frameworks may further support cross-study comparison and methodological clarity. |
| 33. | 490 onwards | Great links to other work | Thank you for this positive feedback! |  |
| 34. | 520 | Okay, this kind of addresses a comment I had earlier about agent vs broader intervention. Maybe bring this up earlier in your methods? | Thank you for this helpful suggestion. We agree that this clarification should appear earlier in the manuscript. We therefore added a sentence explaining this in the Methods section (data synthesis). | (269) Intervention components were also coded according to whether they were delivered directly by the virtual agent or by other elements of the broader digital intervention system. In some studies – particularly older or hybrid intervention designs – the reporting of the system architecture was limited, which made this distinction less explicit. The found combinations were then charted to identify frequently occurring patterns across intervention contexts (e.g., diabetes vs. cancer management). |

**Response to Reviewer 2 – Graziella Zangger**

| # | Line | Reviewer Comment | Response | Manuscript changes |
| --- | --- | --- | --- | --- |
| Major Comments | |  | | |
| 1. |  | The introduction highlights personalisation as a key promise and gap, yet the research questions primarily focus on describing combinations and prevalence of BCTs, health data types, and delivery channels. Clarifying whether the review aims mainly to describe intervention architectures or to examine how personalisation is operationalised could help strengthen coherence between the rationale and the research questions. | Thank you for this helpful observation. To strengthen coherence between the introduction and research questions, we clarified the aim of the review shortly before introducing the research questions. | (157) Thus, this systematic scoping review aims to shed light on the intervention architectures underlying virtual agent-delivered chronic care interventions and to explore their patterns by addressing the following questions: |
| 2. |  | The manuscript tends to treat personalisation, BCTs, virtual agents, and delivery channels as tightly coupled elements. A clearer conceptual description of this would strengthen the theoretical clarity and interpretability. The authors might consider briefly illustrating how they see the relationship between these elements, for example, through a simple conceptual model showing how personalisation, BCTs, virtual agents, and delivery channels interact. This would make the analytical logic more explicit for the reader, as they might view these concepts as more distinct layers. | Thank you for this helpful suggestion. In response to this, as well as a similar comment from reviewer 1 (see their comment 4. & 5.), we clarified the analytical logic of the review in the introduction by explicitly describing virtual agent-delivered interventions as systems composed of three interacting components: health data, behavior change techniques (BCTs), and delivery channels. In addition, we introduced a conceptual figure (**Figure 1**) that visually illustrates how these components relate to each other within the architecture of virtual agent-delivered interventions. We hope this added explanation and figure help make the analytical logic of the review more explicit. | (154) In this systematic scoping review, virtual agent-delivered interventions are conceptualized as systems composed of three interacting components: health data inputs, BCTs, and delivery channels through which these strategies are implemented. **Figure 1** illustrates how virtual-agent delivered interventions can be conceptualized as the integration of these three interacting components. |
| 3. |  | The review is described as a “systematic scoping review”, yet no grey literature search is reported. While grey literature is not mandatory, it is often recommended for scoping reviews to support comprehensiveness. The authors may wish to justify its exclusion or reconsider the use of the term “systematic” and add this as a limitation of the paper. | Thank you for this helpful suggestion. Regarding the gray literature, our objective was to map empirically validated virtual agent-delivered interventions that reported longitudinal testing in real-world settings. We therefore restricted inclusion to peer-reviewed empirical studies to ensure sufficient methodological detail, intervention transparency, and outcome reporting. We have clarified this rationale in the methods section and acknowledged this as a limitation in the discussion section. | (202) Given the focus on empirically evaluated virtual agent–delivered interventions with longitudinal or real-world testing, gray literature was excluded to ensure sufficient methodological detail and transparency for the structured extraction of intervention components.  (561) Finally, the search was restricted to peer-reviewed publications and did not include gray literature, which may have excluded developments reported in industry reports or non-academic sources. |
| 4. |  | Further clarification regarding the search process would be helpful, including whether the search was conducted from database inception or within a specific time frame, and whether reference lists of included studies were screened to identify additional relevant papers. | Thank you for these helpful remarks (addressing comment 4 and 5 below). We have clarified several aspects of the search strategy in the Methods section by specifying that no date restrictions were applied and that studies were considered from the earliest records available in each database up to the search date. We also clarified that the listed chronic conditions in the search string represent examples used to increase search sensitivity, while broader terms such as “chronic disease” and “chronic condition” ensured coverage across a wide range of conditions. | (190) Chronic conditions (e.g., “diabetes”, “heart disease, “cancer”, “COPD”), next to broader terms such as “chronic disease” and “chronic condition” to ensure coverage across a wide range of chronic conditions. The final selection of keywords was informed by prior reviews in the field of conversational agents in healthcare (e.g., Martinengo et al., , Tudor Car et al. 2020; Jiang et al.; 2024) and consultation of an information specialist and disciplinary experts. (195) The reference lists of included studies were also screened to identify additional relevant publications. (199) No date restrictions were applied, meaning studies were considered from the earliest records available in each database up to the search date (10^th^ September 2024). Only English-language, peer-reviewed studies available in full-text were included. |
| 5. |  | While the PCC framework is appropriate, the operationalization of search terms appears somewhat inconsistent. The selection of chronic conditions seems selective and does not clearly reflect the most common chronic diseases, and predefined context terms (e.g., telehealth, digital health) may unintentionally restrict exploration of diverse contexts. Providing a clearer rationale for these choices would strengthen the transparency of the search strategy. | Addressed in comment 4 above. | Addressed in comment 4 above. |
| 6. |  | Although BCTTv1 is referenced, the coding procedure would benefit from greater transparency. The extracted BCT categories currently mix formal taxonomy labels with broader constructs (e.g., motivation, self-regulation), which are not discrete BCT categories. Aligning extracted techniques more closely with official BCTTv1 terminology would improve conceptual precision. | Thank you for this helpful observation. Upon revisiting the tables and terms used, we noticed that the label “motivation/reinforcement” appeared due to a wording error during table preparation. This construct was not used as a coded BCT category in the analysis. The BCTs were coded using the BCT Taxonomy v1 (BCTTv1) using those categories (e.g., self-monitoring, feedback on behavior, instruction on how to perform a behavior, prompts/cues). We have corrected the table accordingly to ensure alignment with official BCTTv1 terminology. | (280) See Hauser-Ulrich et al. in **Table 4**. |
| 7. |  | To further strengthen rigor and reproducibility, the authors may also consider clarifying whether coders had formal training or prior experience with BCTTv1, and briefly describing how coding consistency and any disagreements were handled. And argue how coding may have affected the results. | Thank you for this suggestion. As also highlighted in response to Reviewer 1 (comment 10), we have clarified the coding procedure and authorship expertise in the Methods section. We also added a clarification sentence in the strengths & limitations section of the discussion, acknowledging how coding may have influenced the results. | (234) BCT identification and coding were performed by the first author, who has a background and experience with behavior change intervention design using the BCT Taxonomy and the CeHRes Roadmap. Coding decisions were reviewed and discussed with the third co-author (SK), with expertise in eHealth intervention design to ensure consistency and validity. Any uncertainties in classification were resolved through discussion and consensus. The main extraction categories (e.g., BCTs, health data, delivery channels) were defined a priori based on the study objectives and existing frameworks, while subcategories were refined iteratively during the extraction process (e.g., differentiation between health data input and output).  (554) Because many studies did not explicitly label BCTs, coding relied on mapping intervention descriptions to BCTTv1 definitions, which may have influenced the frequency with which certain techniques were identified. |
| 8. |  | Presenting a study-level overview of which BCTs were coded in each intervention could substantially enhance transparency and practical usefulness, particularly for readers seeking to apply these findings in future research or design work. | Thank you for this helpful suggestion. To improve transparency, we added a study-level overview table detailing the BCTs identified in each intervention.  The table includes the coded BCTs per study, whether they were explicitly reported in text or inferred by the authors, and excerpts from the original study descriptions that informed the coding decisions. | [added: supplementary file **S5 BCT coding evidence**]  (353) Examples of how intervention features were mapped to BCTTv1 categories are provided in **S5 BCT coding evidence**. |
| 9. |  | There is noticeable overlap between the sections on “Use of BCT, health data & delivery channel combinations” and “Patterns & contexts of use”. While they are intended to address different objectives, both sections repeatedly describe similar features, which blur the analytical distinction. Clarifying the specific focus of each section and reducing repeated descriptive content would improve readability and narrative flow. | Thank you for this helpful observation. We agree that the distinction between these sections should be clear. In response, we reduced repeated descriptive content in the “Patterns & contexts of use” section and clarified its focus. Specifically, this section now emphasizes the contexts in which the previously identified combinations are applied (e.g., chronic condition focus, intervention aims, and settings), rather than reiterating the combinations themselves. | Removed 281-291 (before paragraph 332) |
| 10. |  | The manuscript would benefit from clearer positioning in relation to previous work, particularly Martinengo et al. (2022) and Jiang et al (2024). More explicitly outlining how this review extends or differs from existing scoping reviews on conversational agents and BCTs would strengthen the contribution. | Thank you for this helpful suggestion. We added a short paragraph at the beginning of the Discussion to more clearly position this review relative to previous work of Martinengo et al. (2022) and Jiang et al. (2024). In addition to this, we also added a paragraph to the introduction (in response to comment 8 by reviewer 1) summarizing prior findings that demonstrate inconsistent reporting of theoretical foundations in conversational agent and digital health interventions in the chronic care context, as well as evidence suggesting that stronger theoretical grounding is associated with improved intervention outcomes (e.g., in other contexts such as eating disorder interventions). In addition, we clarified that there is limited prior work systematically categorizing design rationales in this context, which motivated our exploratory approach to mapping these rationales. This provides clearer justification for RQ1, and underlines how this review differs from existing reviews more clearly. | (439) Previous reviews have examined conversational agents in healthcare and their use of behavior change techniques. For example, Martinengo et al. (2024) reviewed conversational agent interventions across a broad range of health domains and identified commonly used BCTs, while Jiang et al. (2022) focused on the development and evaluation characteristics of ECAs for chronic diseases. In contrast, the present review focuses specifically on virtual agent interventions for physical chronic conditions and extends prior work by systematically examining how health data inputs are combined with BCTs and delivery channels within intervention architectures.  (143)(…) In addition, the theoretical and practical rationales underlying these design choices are often insufficiently articulated. Reviews of conversational agents in chronic healthcare have found that a substantial proportion of studies are not explicitly informed by behavior change theory (Martinengo et al. 2022; Salas-Groves et al., 2023). At the same time, evidence from other domains suggests that stronger theoretical grounding may enhance intervention effectiveness (Webb et al., 2010). For example, a meta-analysis of digital eating disorder interventions found that interventions with higher theory coding scheme scores demonstrated significantly greater improvements in outcomes compared to less theory-informed interventions (Thomas et al., 2024). Together, these findings highlight the importance of examining how design decisions in virtual agent-delivered chronic care interventions are justified. However, limited prior work has systematically categorized how such rationales are articulated in virtual agent-delivered interventions, indicating a need for exploratory mapping approaches. |
| 11. |  | The conclusion points in an important direction but remains relatively broad. Given the detailed analysis presented, the authors are well positioned to offer more concrete guidance, for example by suggesting clearer reporting of BCTs using BCTTv1 terminology, structured mapping of BCT–health data–delivery pathways, or guidance for reporting virtual agent architectures. Such specificity would increase the practical value of the review for both researchers and designers. The authors may also want to point to the limiting papers within each chronic condition. | Thank you for this helpful observation. We strengthened the conclusion by adding more concrete recommendations for reporting and design of virtual agent-delivered interventions. To clarify the scope of the evidence base, we also added a sentence noting that due to the limited number of studies per condition, condition-specific patterns should be interpreted cautiously. We also slightly expanded a prior section of the Discussion to further elaborate on how BCTs may operate synergistically with integrated system interactions. | (559) Because the number of studies per chronic condition was limited, the patterns identified in this review should be interpreted cautiously.  (575) To support transparency and reproducibility in future research, we recommend that intervention studies explicitly report BCTs using standardized taxonomies such as the BCTTv1, clearly describe how health data are used to tailor virtual agent interactions, and provide structured descriptions of how BCTs, health data inputs, and delivery channels are integrated within the virtual agent architecture. Such reporting would facilitate comparison across studies and support the development of more evidence-informed design strategies for different chronic conditions.  (482) However, our findings suggest that not only the number of BCTs, but also the way they are combined and embedded within system interactions may matter. In many of the reviewed studies, BCTs such as feedback, instruction on how to perform a behavior and prompts/cues were integrated within a single interaction flow, indicating that these elements may operate synergistically rather than as isolated components. |
| 12. |  | Statements suggesting increasing complexity of interventions over time would benefit from clearer empirical justification or explicit trend analysis to support these claims. | Thank you for this important comment. We agree that statements suggesting temporal trends (e.g., increasing intervention complexity) require explicit trend analyses, which were not conducted in this review. We have therefore revised the manuscript to soften such phrasing and instead describe these observations as patterns or variations across studies we included in our review. | (571) Foundational BCTs like self-monitoring, feedback, instruction on how to perform a behavior and prompts and cues were most commonly observed across interventions, while more complex techniques and adaptive architectures appeared in a subset of studies, particularly in more adaptive or AI-enhanced systems. |
| 13. |  | The relationship between the narrative thematic analysis of design rationale and the structured extraction of intervention components is not fully articulated. Clarifying how these two analytic layers inform each other could strengthen methodological coherence. | Thank you for this helpful suggestion. We agree and clarified the relationship between the two analyses in the opening sentence of the Discussion. | (425) This systematic scoping review mapped how BCTs, health data, and delivery channels were used in virtual agent interventions for chronic condition management across 21 studies. While the analysis of design rationales provided contextual insight into how authors justified intervention design choices, the structured extraction of BCTs, health data, and delivery channels allowed a complementary mapping of how these components were operationalized within the interventions. |
| 14. |  | While theory is discussed narratively, it is not extracted as a structured variable. Systematically documenting which theories underpin which interventions may allow clearer comparison and enhance the analytical depth of the review. | Thank you for this helpful suggestion. References to behavioral theories were indeed captured as part of the intervention design rationale analysis. However, theories were not extracted as a separate structured variable later, because their reporting was highly inconsistent across studies and often limited to brief mentions. In addition, the analytical focus of this review was on the interaction between BCTs, health data, and delivery channels as core components of intervention architectures. Expanding the extraction to include detailed categorization of underlying theories would have extended beyond this scope and reduced clarity of the primary analytical focus. |  |
| Minor Comments | |  | | |
| 15. |  | Given that mental health conditions are excluded as primary diagnoses, the use of the broad term “chronic conditions” may be slightly misleading, as mental health disorders are commonly considered chronic. The authors may wish to clarify this by specifying “somatic” or “physical” chronic conditions, or briefly justify the scope in the title or introduction. | Thank you for this helpful comment. We now clarified the scope of the review in the Introduction by specifying that it focuses on physical chronic conditions. This ensures consistency between the terminology used and the inclusion criteria. | (70) In this review, the focus is on virtual agent-delivered interventions targeting physical chronic conditions, such as cardiovascular diseases, diabetes, cancer, and chronic pain. |
| 16. |  | There is noticeable variation in terminology (e.g., virtual agents, chatbots, AI agents, conversational systems, but also for the terms chronic condition, disease, chronic care populations). Establishing clear definitions early and using consistent terminology throughout would improve coherence. | Thank you for these thoughtful suggestions (also addressing comments 17. & 18.). We have improved clarity and consistency throughout the manuscript by defining key terms in the Introduction and standardizing terminology (e.g., using “virtual agent-delivered interventions” and “chronic conditions” consistently). In the results tables, terminology such as “chatbot” was retained where it directly reflected the terminology used in the original studies. This was done to preserve the accuracy of data extraction, while overarching terminology (e.g., “virtual agent-delivered interventions”) was now applied consistently in the narrative text. In addition we added footnotes in all tables to define abbreviations and improve accessibility for an interdisciplinary audience. | Examples throughout text:  (63) virtual agent interventions -> virtual agent-delivered interventions  (95) chronic disease management -> chronic condition management  (Table 2 Footnote): Abbreviations: CA = conversational agent; ECA = embodied conversational agent; AI = artificial intelligence; IoT = Internet of Things; NLP = natural language processing; NLU = natural language understanding; PDA = personal digital assistant; T1D = type 1 diabetes; T2D = type 2 diabetes; RCT = randomized controlled trial. |
| 17. |  | Consider whether “virtual agent-delivered interventions” might provide clearer precision. | Addressed in comment 16 above. | Addressed in comment 16 above. |
| 18. |  | Several tables contain abbreviations that are not defined. Adding legends or footnotes explaining all abbreviations would improve accessibility, particularly for an interdisciplinary audience. | Addressed in comment 16 above. | Addressed in comment 16 above. |
| 19. |  | Claims of being the first comprehensive synthesis may benefit from slight tempering, given limitations in search breadth, transparency of BCT coding, and prior papers on agents and BCTs. | Thank you for this helpful comment. We have tempered claims regarding novelty to more accurately position our contribution in relation to existing literature. | (539) To our knowledge, this review is among the first to systematically map the use of behavior change techniques, health data, and delivery channels in virtual agent-delivered interventions specifically targeting chronic condition management |
