# Supplementary material for: Mapping behavior change techniques and health data combinations in virtual agents for chronic condition management: A systematic scoping review
Source: PLOS Digit Health. 2026 Jul 28;5(7):e0001604. doi: 10.1371/journal.pdig.0001604 (PMC13411939; doi:10.1371/journal.pdig.0001604)
Supplement: Peer review history

# Peer review: Decision Letter 0

**DOI:** 10.1371/journal.pdig.0001604.r001

**Authors:** Saif Khairat, Zhao Ni

22 Jan 2026

PDIG-D-25-00689

Mapping behavior change techniques and health data combinations in virtual agents for chronic condition management: a systematic scoping review

PLOS Digital Health

Dear Dr. Kreuzberg,

Thank you for submitting your manuscript to PLOS Digital Health. After careful consideration, we feel that it has merit but does not fully meet PLOS Digital Health's publication criteria as it currently stands. Therefore, we invite you to submit a revised version of the manuscript that addresses the points raised during the review process.

Please submit your revised manuscript by Mar 23 2026 11:59PM. If you will need more time than this to complete your revisions, please reply to this message or contact the journal office at digitalhealth@plos.org. When you're ready to submit your revision, log on to https://www.editorialmanager.com/pdig/ and select the 'Submissions Needing Revision' folder to locate your manuscript file.

Please include the following items when submitting your revised manuscript:

* A rebuttal letter that responds to each point raised by the editor and reviewer(s). You should upload this letter as a separate file labeled 'Response to Reviewers'. This file does not need to include responses to any formatting updates and technical items listed in the 'Journal Requirements' section below.

* A marked-up copy of your manuscript that highlights changes made to the original version. You should upload this as a separate file labeled 'Revised Manuscript with Track Changes'.

* An unmarked version of your revised paper without tracked changes. You should upload this as a separate file labeled 'Manuscript'.

If you would like to make changes to your financial disclosure, competing interests statement, or data availability statement, please make these updates within the submission form at the time of resubmission. Guidelines for resubmitting your figure files are available below the reviewer comments at the end of this letter.

We look forward to receiving your revised manuscript.

Kind regards,

Saif Khairat

Academic Editor

PLOS Digital Health

Saif Khairat

Academic Editor

PLOS Digital Health

Leo Anthony Celi

Editor-in-Chief

PLOS Digital Health

orcid.org/0000-0001-6712-6626

**Journal Requirements:**

If the reviewer comments include a recommendation to cite specific previously published works, please review and evaluate these publications to determine whether they are relevant and should be cited. There is no requirement to cite these works unless the editor has indicated otherwise.

**Additional Editor Comments (if provided):**

Thank you for submitting your manuscript, which addresses an important and timely topic with strong potential to advance the design and reporting of virtual agent interventions for chronic condition management. To strengthen its clarity, rigour, and practical value, please clarify the conceptual focus on personalisation and consider adding a simple model illustrating relationships among key elements; enhance methodological transparency by detailing the search strategy, justifying the exclusion of grey literature, and aligning BCT coding strictly with BCTTv1 taxonomy while explaining coder training and reliability checks; reduce redundancy between overlapping sections and clarify how thematic and structured analyses relate; position the work more explicitly against prior reviews (e.g., Martinengo et al., Jiang et al.) and temper novelty claims; and offer more concrete guidance in the conclusion, such as structured mapping and reporting standards. Minor revisions include consistent terminology, defining abbreviations, and clarifying scope regarding chronic conditions. Addressing these points will significantly improve the manuscript’s impact and usability.

[Note: HTML markup is below. Please do not edit.]

**Reviewers' Comments:**

Reviewer's Responses to Questions

**Comments to the Author**

1. Does this manuscript meet PLOS Digital Health’s publication criteria? Is the manuscript technically sound, and do the data support the conclusions? The manuscript must describe methodologically and ethically rigorous research with conclusions that are appropriately drawn based on the data presented.

Reviewer #1: Yes

Reviewer #2: Yes

**********

2. Has the statistical analysis been performed appropriately and rigorously?

Reviewer #1: Yes

Reviewer #2: N/A

**********

3. Have the authors made all data underlying the findings in their manuscript fully available (please refer to the Data Availability Statement at the start of the manuscript PDF file)?

The PLOS Data policy requires authors to make all data underlying the findings described in their manuscript fully available without restriction, with rare exception. The data should be provided as part of the manuscript or its supporting information, or deposited to a public repository. For example, in addition to summary statistics, the data points behind means, medians and variance measures should be available. If there are restrictions on publicly sharing data—e.g. participant privacy or use of data from a third party—those must be specified.

Reviewer #1: Yes

Reviewer #2: Yes

**********

4. Is the manuscript presented in an intelligible fashion and written in standard English?

PLOS Digital Health does not copyedit accepted manuscripts, so the language in submitted articles must be clear, correct, and unambiguous. Any typographical or grammatical errors should be corrected at revision, so please note any specific errors here.

Reviewer #1: Yes

Reviewer #2: Yes

**********

5. Review Comments to the Author

Please use the space provided to explain your answers to the questions above. You may also include additional comments for the author, including concerns about dual publication, research ethics, or publication ethics. (Please upload your review as an attachment if it exceeds 20,000 characters)

Reviewer #1: Overall comments

- Wow, this is a huge and highly impressive undertaking! Congrats. I have lots of comments below, but please take them as a sign that I really enjoyed this paper and want it to be published in the best form it can be. Because you have several complex objectives with this scoping review, clarity is key. I found myself lost in the results section sometimes. I have tried my best to outline ways you can make it as clear as possible, so it’s easier to digest for a first-time reader. Good luck with the edits! Looking forward to seeing the final product. Please see section- and line-based comments below.

Abstract

1. 35- Define virtual agents briefly if you can.

2. 36-37 - Do you mean the ways in which distinct components within chronic care interventions or within virtual agent interventions that support chronic care…? Be precise here.

3. 41-Comprehensive search can probably just be search. Can a search every truly be comprehensive? Also, what about gray literature?

4. 43- You say the first step, but no other steps are listed.

5. 44 - By intervention design do you mean virtual agent design?

Text

1. Line 76 - Provide reference for “which is essential for behaviour change”

2. Line 81-82 - People who aren’t experts won’t know what these different types mean or how they are different from each other.

3. Lines 105-107 – Are there other reviews on personalization to reference?

4. Lines 110 – it’s not clear what you mean by “this” in “… examples how THIS is applied in virtual agent delivered interventions”… Can you be more precise?

5. Lines 111-124 – It seems like, at this point in the introduction, the point of this review is to understand personalization approaches are applied to virtual agent delivered approaches….

6. Lines 110-125 - Maybe these paragraphs could be a table?

7. Lines 132-135 – So far these lines seem to communicate the central focus of the paper: how (a) BCTs and personalized health data are (b) delivered through different delivery channels, within (c) virtual agents, to (d) impact behaviour change outcomes… I would make this crystal clear and use the same language in the abstract and all of the text. I think you reframe your objective in the review several times in slightly different ways, which is confusing. In the abstract your language is: to map how (a) BCTs, health data, and delivery channels are (b) rationalized and (b) integrated into (d) virtual agent delivered interventions for chronic condition management. Just be consistent throughout.

8. RQ1 - I feel like when you introduce RQ1 about justification/rationale it comes as a bit of a surprise as you didn’t talk about the issue of rationale and justification in the introduction very much. Is there previous research that has tried to map rationale and how rationales are categorized?

9. RQ2 – Based on this question it seems like you are looking at BCTs by their (a) association with certain types of health data, and (b) with certain types of delivery channels? I feel like this organization didn’t come across in your introduction. Here it makes it seem like BCTs are the foundational focus and you will describe them by these 2 different ways. As I said above, just be consistent. This also makes me think some sort of visual diagram might be helpful to outline the logic of this review?

10. Lines 176 – Eligibility Criteria. For “Utilize BCTs”, I think this might require more explanation. How would you decide on an inferred BCT? Also, if they just referenced behavioural theory in the intro but didn’t use a BCT, how would this be treated? Did you have an expert in behavioural science? Also I think in the introduction you mentioned behaviour change outcomes only, not health outcomes and user experience outcomes. Consider outlining somewhere in your methods what the authorship expertise is and how you brought your own experiences into the eventual coding of things…

11. Lines 202- Data extraction items. With BCTs, how did you infer BCTs from intervention descriptions? As stated above, this usually requires behavioural science expertise on the authorship team. For other categories, can you explain if the categories were decided on a priori or as extraction was happening? (e.g., health data integration).

12. Line 207 – Make sure your introduction text aligns with this table.

13. Line 217 – If you are referencing the narrative thematic analysis method, you will need to reference this with a methodological paper and explain your application of the referenced method with a bit more detail.

14. Lines 220 – This coding scheme is very interesting! Do you mean the study’s rationale for building the virtual agent? If so, its hard for me to imagine what some of these rationale categories would look like… For instance, what do you mean by practice-driven rationale… Can you be a bit more clear with your examples for each categorization? Hard for me to picture.

15. Line 268-274 – This is really interesting and consistent with other reviews I have seen. I would love you to discuss in the discussion section the implications of rationale only provided at the intervention level and not for specific choices made about BCTs, health data, and delivery channels. (You do this! So ignore).

16. Line 280 – Intervention Design Rationale Table. As I stated earlier, it would be nice to have some more details about these categories (what they include and exclude).

17. Line 280 - It also appears some papers might have discussed rationales at a more specific level than the intervention as a whole, as you describe, for example, that Roca et al had “functionalities developed iteratively with nurses”. For papers that did go into more specific rationales beyond the broad intervention level, did you categorize these? …. I see later in the text you say “the rationale for use of individual intervention components like health data types and delivery channels was often implied through functionality or usability features, but not systematically articulated.” I think I see where you are going but it’s not entirely clear the distinctions here. Maybe just a bit more clarity would help.

18. Lines 281-291 - I am not sure how much more detail these paragraphs are providing beyond what is already in the tables? I talk about this later, but if you are worried about word count, try to be less repetitive in your text when its already in the tables.

19. Line 292- I thought you were reporting connections in another section/ addressing another RQ besides RQ1. This paragraph seems misplaced? Or maybe it just needs to be more obvious you were reviewing rationales for connections?

20. Line 303 – Can you put the references for the 3 studies that explicitly reported BCTs?

21. Line 311 -312 – I think this distinction is important and could be make clearer throughout the entire paper, as I am assuming many of the interventions had virtual agent components and non-virtual agent components. It makes me think that your section above on intervention rationale should either (a) just focus on the rationale for virtual agents or (b) be very clear that it was the entire intervention which included some parts that included a virtual agent.

22. 313 - Table 4 – For BCTs used, do you have a supplementary appendix you could add that outlines the inferring of these BCTs and what/how you coded as “self-monitoring” ,“feedback” etc. I think this would be helpful for readers to be transparent about how different features were coded as specific BCTs.

23. 316-356 – These results are very interesting and answered some initial concerns I had about outlining how you categorized features of the virtual agents into BCT categories. I wonder if it would be easier for the reader if this section had a table that outlined the (1) the BCT, (2) the operationalization of the BCTs in the virtual agent (and out), (3) the delivery channels of the operationalizations of the BCT… or something like this... I feel like this written section has a lot of useful synthesis and just providing a table at the study level doesn’t do enough.

24. 358 – Just to confirm these combination types and frequencies are just for the virtual agents or the interventions as a whole? I think I keep getting confused what level the results are referring to in the written text.

25. 358- 403 – I am a bit confused on the counting in these clusters in terms of studies that could be double counted. For example, for the 10 studies that included self-monitoring, feedback, and instruction, where these 10 also all included in the count of studies with self-monitoring and feedback? Or are these clusters distinct?

26. 404 Table 5 – I think including prevalence more obviously in this table would help, as this was within RQ3s main objective.

27. 404 - Overall, I feel like there is a lot of dense text which is often very duplicative of the tables and maybe adds more confusion than clarity. It’s hard to know when I am reading new, important information in the text that is not in the tables or when it is just easily repeated in the tables. This is a theme throughout your results sections.

28. Line 434-426 – This is a key finding, great!

29. Line 451-454 – Very important! I actually think you are understating an important implication of your work by referencing Eaton who observed that more BCTs were better… Your study is starting to get at this idea that not just more is better, but certain combinations of cohesive intervention strategies that can be delivered together in a meaningful tandem might make the difference… where the whole is greater (?) than the sum of the individual parts and something new might be created… Obviously, you can’t prove this in the review, but I wonder if there is any more literature not only on “more BCTs = better”, but “BCT combinations = better”… I am not up to date on this literature, but I am curious…

30. 460 – So BCTs can BE system functionalities themselves. I think the conversation in this paragraph makes me think about whether its actually a useful practice to take an intervention and separate it out into individual BCTs when the reality of the human experience of an intervention is really multiple of these simultaneously, creating something new altogether. Our brains aren’t separately receiving these BCTs anymore… (I wonder if there are any cognitive studies on this…) What implications does this result have for mechanisms of action being leveraged and the field of applied behavioural science more generally I wonder?

31. Lines 473-475 – Hm, I wonder if this conclusion is correct… Because if we did magically figure out recipes for BCT, data, channel combinations for different chronic conditions, this MIGHT enable evidence-based virtual agent development, but it also wouldn’t leave room for creativity and design thinking related “evidence” to be included in future intervention design. We would end up with a phone book or rule book for design which doesn’t really encompass realities of human experiences and constant political, cultural, or social changes. I don’t know if a static rulebook could really ever be made…

32. Lines 476-477 – Interesting! Would love to know your thoughts on this re: next steps and future directions…

33. 490 onwards – Great links to other work

34. 520 – Okay, this kind of addresses a comment I had earlier about agent vs broader intervention. Maybe bring this up earlier in your methods?

Reviewer #2: Overall impression

The manuscript, Mapping behavior change techniques and health data combinations in virtual agents for chronic condition management: a systematic scoping review, addresses a timely and relevant topic and explores how virtual agent-delivered interventions combine behaviour change techniques, health data, and delivery channels. The move beyond single-component descriptions towards examining combinations is a strong and welcome approach, and the manuscript reflects careful work and a clear ambition to improve transparency and structure in this field.

Overall, this is a strong and relevant piece of work with clear potential to influence how virtual agent interventions in chronic care are designed and reported. With some refinement, particularly around conceptual clarity and methodological transparency, the manuscript could make a meaningful and lasting contribution. The comments below are intended to support clarity, methodological rigor, and practical usefulness, and to help further strengthen the paper.

Major comments

1. The introduction highlights personalisation as a key promise and gap, yet the research questions primarily focus on describing combinations and prevalence of BCTs, health data types, and delivery channels. Clarifying whether the review aims mainly to describe intervention architectures or to examine how personalisation is operationalised could help strengthen coherence between the rationale and the research questions.

2. The manuscript tends to treat personalisation, BCTs, virtual agents, and delivery channels as tightly coupled elements. A clearer conceptual description of this would strengthen the theoretical clarity and interpretability. The authors might consider briefly illustrating how they see the relationship between these elements, for example, through a simple conceptual model showing how personalisation, BCTs, virtual agents, and delivery channels interact. This would make the analytical logic more explicit for the reader, as they might view these concepts as more distinct layers.

3. The review is described as a “systematic scoping review”, yet no grey literature search is reported. While grey literature is not mandatory, it is often recommended for scoping reviews to support comprehensiveness. The authors may wish to justify its exclusion or reconsider the use of the term “systematic” and add this as a limitation of the paper.

4. Further clarification regarding the search process would be helpful, including whether the search was conducted from database inception or within a specific time frame, and whether reference lists of included studies were screened to identify additional relevant papers.

5. While the PCC framework is appropriate, the operationalisation of search terms appears somewhat inconsistent. The selection of chronic conditions seems selective and does not clearly reflect the most common chronic diseases, and predefined context terms (e.g., telehealth, digital health) may unintentionally restrict exploration of diverse contexts. Providing a clearer rationale for these choices would strengthen the transparency of the search strategy.

6. Although BCTTv1 is referenced, the coding procedure would benefit from greater transparency. The extracted BCT categories currently mix formal taxonomy labels with broader constructs (e.g., motivation, self-regulation), which are not discrete BCT categories. Aligning extracted techniques more closely with official BCTTv1 terminology would improve conceptual precision.

7. To further strengthen rigor and reproducibility, the authors may also consider clarifying whether coders had formal training or prior experience with BCTTv1, and briefly describing how coding consistency and any disagreements were handled. And argue how coding may have affected the results.

8. Presenting a study-level overview of which BCTs were coded in each intervention could substantially enhance transparency and practical usefulness, particularly for readers seeking to apply these findings in future research or design work.

9. There is noticeable overlap between the sections on “Use of BCT, health data & delivery channel combinations” and “Patterns & contexts of use”. While they are intended to address different objectives, both sections repeatedly describe similar features, which blur the analytical distinction. Clarifying the specific focus of each section and reducing repeated descriptive content would improve readability and narrative flow.

10. The manuscript would benefit from clearer positioning in relation to previous work, particularly Martinengo et al. (2022) and Jiang et al (2024). More explicitly outlining how this review extends or differs from existing scoping reviews on conversational agents and BCTs would strengthen the contribution.

11. The conclusion points in an important direction but remains relatively broad. Given the detailed analysis presented, the authors are well positioned to offer more concrete guidance, for example by suggesting clearer reporting of BCTs using BCTTv1 terminology, structured mapping of BCT–health data–delivery pathways, or guidance for reporting virtual agent architectures. Such specificity would increase the practical value of the review for both researchers and designers. The authors may also want to point to the limiting papers within each chronic condition.

12. Statements suggesting increasing complexity of interventions over time would benefit from clearer empirical justification or explicit trend analysis to support these claims.

13. The relationship between the narrative thematic analysis of design rationale and the structured extraction of intervention components is not fully articulated. Clarifying how these two analytic layers inform each other could strengthen methodological coherence.

14. While theory is discussed narratively, it is not extracted as a structured variable. Systematically documenting which theories underpin which interventions may allow clearer comparison and enhance the analytical depth of the review.

Minor comments

15. Given that mental health conditions are excluded as primary diagnoses, the use of the broad term “chronic conditions” may be slightly misleading, as mental health disorders are commonly considered chronic. The authors may wish to clarify this by specifying “somatic” or “physical” chronic conditions, or briefly justify the scope in the title or introduction.

16. There is noticeable variation in terminology (e.g., virtual agents, chatbots, AI agents, conversational systems, but also for the terms chronic condition, disease, chronic care populations). Establishing clear definitions early and using consistent terminology throughout would improve coherence.

17. Consider whether “virtual agent-delivered interventions” might provide clearer precision.

18. Several tables contain abbreviations that are not defined. Adding legends or footnotes explaining all abbreviations would improve accessibility, particularly for an interdisciplinary audience.

19. Claims of being the first comprehensive synthesis may benefit from slight tempering, given limitations in search breadth, transparency of BCT coding, and prior papers on agents and BCTs.

**********

6. PLOS authors have the option to publish the peer review history of their article (what does this mean?). If published, this will include your full peer review and any attached files.

**Do you want your identity to be public for this peer review?**  If you choose “no”, your identity will remain anonymous but your review may still be made public.

For information about this choice, including consent withdrawal, please see our Privacy Policy.

Reviewer #1: **Yes:**  Paula Voorheis

Reviewer #2: **Yes:**  Graziella Zangger

**********

[NOTE: If reviewer comments were submitted as an attachment file, they will be attached to this email and accessible via the submission site. Please log into your account, locate the manuscript record, and check for the action link "View Attachments". If this link does not appear, there are no attachment files.]

**Figure resubmission:**

While revising your submission, we strongly recommend that you use PLOS’s NAAS tool (https://ngplosjournals.pagemajik.ai/artanalysis) to test your figure files. NAAS can convert your figure files to the TIFF file type and meet basic requirements (such as print size, resolution), or provide you with a report on issues that do not meet our requirements and that NAAS cannot fix.

After uploading your figures to PLOS’s NAAS tool - https://ngplosjournals.pagemajik.ai/artanalysis, NAAS will process the files provided and display the results in the "Uploaded Files" section of the page as the processing is complete. If the uploaded figures meet our requirements (or NAAS is able to fix the files to meet our requirements), the figure will be marked as "fixed" above. If NAAS is unable to fix the files, a red "failed" label will appear above. When NAAS has confirmed that the figure files meet our requirements, please download the file via the download option, and include these NAAS processed figure files when submitting your revised manuscript.

**Reproducibility:**

To enhance the reproducibility of your results, we recommend that authors of applicable studies deposit laboratory protocols in protocols.io, where a protocol can be assigned its own identifier (DOI) such that it can be cited independently in the future. Additionally, PLOS ONE offers an option to publish peer-reviewed clinical study protocols. Read more information on sharing protocols at https://plos.org/protocols?utm_medium=editorial-email&utm_source=authorletters&utm_campaign=protocols

# Author comment: Author response to Decision Letter 1

**DOI:** 10.1371/journal.pdig.0001604.r002

24 Mar 2026

Attachment Submitted filename: Response to Reviewers.docx

# Peer review: Decision Letter 1

**DOI:** 10.1371/journal.pdig.0001604.r003

**Authors:** Zhao Ni

7 Jul 2026

Mapping behavior change techniques and health data combinations in virtual agents for chronic condition management: a systematic scoping review

PDIG-D-25-00689R1

Dear Kreuzberg,

We are pleased to inform you that your manuscript 'Mapping behavior change techniques and health data combinations in virtual agents for chronic condition management: a systematic scoping review' has been provisionally accepted for publication in PLOS Digital Health.

Before your manuscript can be formally accepted you will need to complete some formatting changes, which you will receive in a follow-up email from a member of our team.

Please note that your manuscript will not be scheduled for publication until you have made the required changes, so a swift response is appreciated.

IMPORTANT: The editorial review process is now complete. PLOS will only permit corrections to spelling, formatting or significant scientific errors from this point onwards. Requests for major changes, or any which affect the scientific understanding of your work, will cause delays to the publication date of your manuscript.

If your institution or institutions have a press office, please notify them about your upcoming paper to help maximize its impact. If they'll be preparing press materials, please inform our press team as soon as possible -- no later than 48 hours after receiving the formal acceptance. Your manuscript will remain under strict press embargo until 2 pm Eastern Time on the date of publication. For more information, please contact digitalhealth@plos.org.

Thank you again for supporting Open Access publishing; we are looking forward to publishing your work in PLOS Digital Health.

Best regards,

Zhao Ni, PhD, BMed, RN, FAAN, FAHA

Section Editor

PLOS Digital Health

***********************************************************

**Additional Editor Comments (if provided):**

**Reviewer Comments (if any, and for reference):**

Reviewer's Responses to Questions

**Comments to the Author**

1. If the authors have adequately addressed your comments raised in a previous round of review and you feel that this manuscript is now acceptable for publication, you may indicate that here to bypass the “Comments to the Author” section, enter your conflict of interest statement in the “Confidential to Editor” section, and submit your "Accept" recommendation.

Reviewer #2: (No Response)

**********

2. Does this manuscript meet PLOS Digital Health’s publication criteria? Is the manuscript technically sound, and do the data support the conclusions? The manuscript must describe methodologically and ethically rigorous research with conclusions that are appropriately drawn based on the data presented.

Reviewer #2: Yes

**********

3. Has the statistical analysis been performed appropriately and rigorously?

Reviewer #2: Yes

**********

4. Have the authors made all data underlying the findings in their manuscript fully available (please refer to the Data Availability Statement at the start of the manuscript PDF file)?

The PLOS Data policy requires authors to make all data underlying the findings described in their manuscript fully available without restriction, with rare exception. The data should be provided as part of the manuscript or its supporting information, or deposited to a public repository. For example, in addition to summary statistics, the data points behind means, medians and variance measures should be available. If there are restrictions on publicly sharing data—e.g. participant privacy or use of data from a third party—those must be specified.

Reviewer #2: Yes

**********

5. Is the manuscript presented in an intelligible fashion and written in standard English?

PLOS Digital Health does not copyedit accepted manuscripts, so the language in submitted articles must be clear, correct, and unambiguous. Any typographical or grammatical errors should be corrected at revision, so please note any specific errors here.

Reviewer #2: Yes

**********

6. Review Comments to the Author

Please use the space provided to explain your answers to the questions above. You may also include additional comments for the author, including concerns about dual publication, research ethics, or publication ethics. (Please upload your review as an attachment if it exceeds 20,000 characters)

Reviewer #2: Thank you for the detailed response and for the substantial revisions made to the manuscript. I believe the paper has improved considerably, particularly with respect to the conceptual framing, methodological transparency, and the reporting of the BCT coding procedures. The authors have addressed the majority of the comments thoughtfully and have provided clear justifications for their decisions where changes were not made.

Overall, I believe the manuscript makes a valuable contribution to the literature and I can see it being suitable for publication.

I have only a few minor comments remaining for the authors' consideration:

1. Although the search strategy is now described more clearly, I encourage the authors to provide the complete database-specific search strings in the supplementary material. This would further strengthen transparency and reproducibility.

2. Table 3 remains somewhat difficult to navigate in its current format. Consider restructuring the table around the five design rationale categories (theory-driven, practice-driven, empirically-driven, mixed, and not explicitly stated), with studies grouped under each category. This would make the categories the primary synthesis and improve readability while preserving the informative rationale descriptions.

3. The rationale for not extracting theories as a structured variable is understandable. However, given the prominence of theory-driven design rationales in the objectives and in the results, the authors may wish to briefly acknowledge that a more systematic mapping of theories could be a valuable direction for future reviews.

4. Page 8, line 175: it appears that the author name or guideline name associated with reference 34 may be missing from the sentence.

These are relatively minor points and do not substantially affect my overall positive assessment of the manuscript.

**********

7. PLOS authors have the option to publish the peer review history of their article (what does this mean?). If published, this will include your full peer review and any attached files.

**Do you want your identity to be public for this peer review?**  If you choose “no”, your identity will remain anonymous but your review may still be made public.

For information about this choice, including consent withdrawal, please see our Privacy Policy.

Reviewer #2: **Yes:**  Graziella Zangger

**********
